# Low-Dose Aspirin and the Risk of Stroke and Intracerebral Bleeding in Healthy Older People: Secondary Analysis of a Randomized Clinical Trial

**DOI:** 10.1001/jamanetworkopen.2023.25803

**Published:** 2023-07-26

**Authors:** Geoffrey C. Cloud, Jeff D. Williamson, Le Thi Phuong Thao, Cammie Tran, Charles B. Eaton, Rory Wolfe, Mark R. Nelson, Christopher M. Reid, Anne B. Newman, Jessica Lockery, Sharyn M. Fitzgerald, Anne M. Murray, Raj C. Shah, Robyn L. Woods, Geoffrey A. Donnan, John J. McNeil

**Affiliations:** 1 Department of Neuroscience, Central Clinical School, Monash University, Melbourne, Australia; 2 Sticht Center for Healthy Aging and Alzheimer’s Prevention, Department of Internal Medicine, Section on Geriatric Medicine, Wake Forest School of Medicine, Winston-Salem, North Carolina; 3 School of Public Health and Preventive Medicine, Monash University, Melbourne, Australia; 4 Center for Primary Care and Prevention, Brown University School of Public Health, Pawtucket, Rhode Island; 5 Menzies Institute for Medical Research, University of Tasmania, Hobart, Australia; 6 School of Public Health, Curtin University, Perth, Western Australia, Australia; 7 Department of Epidemiology Graduate School of Public Health, University of Pittsburgh, Pittsburgh, Pennsylvania; 8 Department of Medicine, Geriatrics Division, Hennepin HealthCare and Berman Center for Clinical Research, Hennepin HealthCare Research Institute, Minneapolis, Minnesota; 9 Rush Alzheimer’s Disease Center, Rush University Medical Center, Chicago, Illinois; 10 Melbourne Brain Centre, University of Melbourne, Royal Melbourne Hospital, Melbourne, Australia

## Abstract

**Importance:**

Low-dose aspirin has been widely used for primary and secondary prevention of stroke. The balance between potential reduction of ischemic stroke events and increased intracranial bleeding has not been established in older individuals.

**Objective:**

To establish the risks of ischemic stroke and intracranial bleeding among healthy older people receiving daily low-dose aspirin.

**Design, Setting, and Participants:**

This secondary analysis of the Aspirin in Reducing Events in the Elderly (ASPREE) randomized, double-blind, placebo-controlled trial of daily low-dose aspirin was conducted among community-dwelling people living in Australia or the US. Participants were older adults free of symptomatic cardiovascular disease. Recruitment took place between 2010 and 2014, and participants were followed up for a median (IQR) of 4.7 (3.6-5.7) years. This analysis was completed from August 2021 to March 2023.

**Interventions:**

Daily 100-mg enteric-coated aspirin or matching placebo.

**Main Outcomes and Measures:**

Stroke and stroke etiology were predetermined secondary outcomes and are presented with a focus on prevention of initial stroke or intracranial bleeding event. Outcomes were assessed by review of medical records.

**Results:**

Among 19 114 older adults (10 782 females [56.4%]; median [IQR] age, 74 [71.6-77.7] years), 9525 individuals received aspirin and 9589 individuals received placebo. Aspirin did not produce a statistically significant reduction in the incidence of ischemic stroke (hazard ratio [HR], 0.89; 95% CI, 0.71-1.11). However, a statistically significant increase in intracranial bleeding was observed among individuals assigned to aspirin (108 individuals [1.1%]) compared with those receiving placebo (79 individuals [0.8%]; HR, 1.38; 95% CI, 1.03-1.84). This occurred by an increase in a combination of subdural, extradural, and subarachnoid bleeding with aspirin compared with placebo (59 individuals [0.6%] vs 41 individuals [0.4%]; HR, 1.45; 95% CI, 0.98-2.16). Hemorrhagic stroke was recorded in 49 individuals (0.5%) assigned to aspirin compared with 37 individuals (0.4%) in the placebo group (HR, 1.33; 95% CI, 0.87-2.04).

**Conclusions and Relevance:**

This study found a significant increase in intracranial bleeding with daily low-dose aspirin but no significant reduction of ischemic stroke. These findings may have particular relevance to older individuals prone to developing intracranial bleeding after head trauma.

**Trial Registration:**

ISRCTN.org Identifier: ISRCTN83772183

## Key Points

**Question:**

In a primary prevention setting, does long-term, daily low-dose aspirin treatment affect the incidence of stroke or intracerebral bleeding?

**Findings:**

This secondary analysis of a randomized clinical trial including 19 114 older adults found a statistically significant 38% increase in intracranial bleeding resulting from a combination of hemorrhagic stroke and other causes of intracerebral hemorrhage among individuals randomized to aspirin. The difference in incidence of ischemic stroke was not statistically significant.

**Meaning:**

These findings suggest that low-dose aspirin may have no role for the primary prevention of stroke and that caution should be taken with use of aspirin in older persons prone to head trauma (eg, from falls).

## Teaser

The secondary analysis of a randomized clinical trial investigates the effect of low-dose aspirin on incidence of ischemic stroke and intracranial bleeding among healthy older people.

## Introduction

Aspirin is an antiplatelet agent that has been used in low doses (75-100 mg/d) for the prevention of cardiovascular events. Despite some recent unfavorable findings, it continues to be widely used for primary and secondary prevention of stroke.^1,2,3^ Its major adverse effect is an increased bleeding tendency.^4,5,6,7^

Information about the efficacy of low-dose aspirin in the primary prevention of stroke is derived from meta-analyses and results of recent major trials, most of which have been conducted in populations with mean ages younger than 70 years.^7,8,9,10^ Despite some inconsistency, these reports suggest a trend toward reduced ischemic stroke counterbalanced by increased intracerebral and other intracranial hemorrhage, with little overall impact on total stroke incidence.

Clinical characteristics of older individuals include an increased inherent susceptibility to hemorrhage, which may be associated with increased fragility of small blood vessels.^11,12^ In addition, older individuals experience an increased susceptibility to major and minor trauma as a result of falls and other accidents.^13,14^ Together, these outcomes may alter the balance of risks and benefits of an antiplatelet drug, especially if given to individuals at low risk in a primary prevention setting. This concern is relevant given the high stroke risk in older individuals, worldwide increases in populations of older individuals, and the importance of evaluating preventive strategies in this age group.^15,16^

The Aspirin in Reducing Events in the Elderly (ASPREE) trial is the largest randomized controlled trial of low-dose aspirin focused on investigating the balance of risks and benefits of this therapy in an older age group.^6^ In addition to its large size, the ASPREE study design included independent adjudication of stroke and hemorrhagic events by independent expert panels.^17,18^ It was therefore ideally positioned to evaluate the balance of risks and benefits of low-dose aspirin in a primary prevention setting. In this study, we provide a comprehensive report on the incidence of first stroke and bleeding events occurring during the median 4.7 years of follow-up of the trial.

## Methods

The ASPREE study was approved by local ethics committees and registered on ISRCTN.org (ISRCTN83772183). Participants provided written informed consent. This secondary analysis was reported according to the Consolidated Standards of Reporting Trials (CONSORT) reporting guideline.

### Study Design

**Figure 1.**
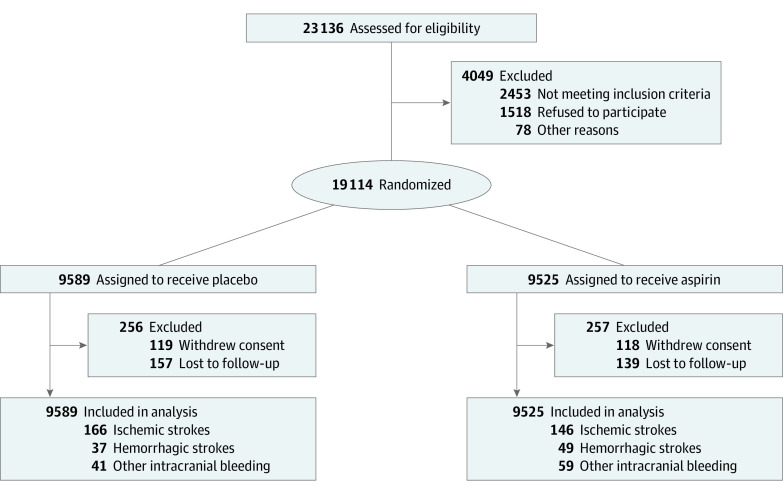
Study Flowchart

The ASPREE study was an investigator-led, prospective, randomized, placebo-controlled trial of daily low-dose aspirin in community-dwelling older adults free of overt cardiovascular disease.^6^ Recruitment to the ASPREE trial took place in Australia and the US between March 2010 and December 2014.^19^ The ASPREE consort flow diagram is shown in Figure 1. Details of the protocol are described in Supplement 1, and the statistical analysis plan was described previously.^18^

### Participants

People aged 70 years or older (≥65 years for US individuals who self-reported their race or ethnicity as African American or Hispanic) without a history of atrial fibrillation, stroke, transient ischemic attack, or myocardial infarction were included. Race and ethnicity were queried in separate questions and combined by ignoring race responses for those responding yes to Hispanic or Latino. Available responses for ethnicity were Hispanic or Latino vs not Hispanic or Latino. Available race categories were Aboriginal or Torres Strait Islander; American Indian; Asian, Black or African American; Native Hawaiian, Other Pacific Islander, or Maori; White; more than 1 race; and unknown or not reported. Aboriginal or Torres Strait Islander; American Indian; Native Hawaiian, Other Pacific Islander, or Maori; more than 1 race or ethnicity; and those who were not Hispanic but reported as other or not reported whose minority status could not be determined were combined as other owing to small population sizes. US members of racial or ethnic minority groups aged 65 to 70 years were included owing to their higher risk of cardiovascular diseases. Other eligibility criteria included systolic blood pressure below 180 mm Hg at entry. All participants were free from dementia and physical disability at enrollment.

### Procedures

Participants were randomized to daily 100-mg enteric-coated aspirin or matching placebo. The ASPREE study design and primary outcome results have been previously described.^6,20,21^

### Outcomes

The primary outcome of ASPREE was disability-free survival (defined as survival free of physical disability and dementia), and this was not different between aspirin and placebo groups. This has been reported, along with overall mortality and cardiovascular outcomes.^6,20,21^

Stroke and hemorrhagic events were predetermined secondary end points. Suspected events within these categories were independently adjudicated by at least 2 members of an expert committee (G.C.C., J.D.W., C.B.E., and G.A.D.). Adjudications were supported by copies of patient routine hospital care records; these included results of key investigations, such as reports from brain and vascular imaging studies and cardiological testing. Disagreements were adjudicated by a third member and consensus reached. Further details of the adjudication processes were described previously.^18^

Using modified Trial of Org 10172 in Acute Stroke Treatment (TOAST) criteria, 4 subgroups of ischemic stroke were reported.^22^ These were cardioembolic (ischemic stroke with a clear cardiac cause, typically atrial fibrillation), large-vessel atherosclerosis (ipsilateral ischemic stroke with a ≥50% extra or intracranial stenosis seen on vascular imaging), small-vessel occlusion (ischemic stroke with a clinical lacunar stroke syndrome and evidence on imaging of a lacunar infarction [subcortical infarct of <15-mm maximal diameter]), and undetermined etiology (reported when ≥2 causes were identified, the evaluation was complete and no cause found, or etiological investigations were incomplete).

Intracranial hemorrhage was reported according to the location identified on imaging (ie, basal ganglionic, lobar, brainstem, or other) or as subarachnoid, subdural, or extradural. Such bleeding was required to have led to hospitalization, prolonged hospitalization, surgery, or death.^23^

### Statistical Analysis

For this analysis, version 3 of the ASPREE data set (released December 2019) was used. This contains more extensive data fields and minor corrections to data in the original version used previously.^6^

Descriptive statistics are presented as frequency and percentage. Cox proportional hazards regression models were used in intention-to-treat analyses to evaluate the effect of aspirin on time to the first ischemic stroke, intracranial bleeding, and intracranial bleeding subtypes.

Cause-specific hazards and cumulative incidence estimates were calculated to account for competing risks (eg, all-cause death and outcomes of the same type when analyzing subtypes for an outcome of interest). With ischemic stroke and first intracranial bleed, we assessed the consistency of treatment effect across several subgroups, including age (65-74, 75-84, and ≥85 years), sex (male and female), smoking status (current and former or never), diabetes, hypertension, dyslipidemia, frailty category, and country (Australia and US). Number needed to treat was calculated based on the restricted mean survival time among individuals assigned to aspirin or placebo.^24^ Analyses were performed using R statistical software version 4.0 (R Project for Statistical Computing) with the following companion packages: survival, prodlim, nnt, and tidyverse. No adjustment was made for multiple statistical tests. A 2 sided *P* value < .05 was used as a cutoff for statistical significance. This analysis was completed from August 2021 to March 2023.

## Results

Of 19 114 participants (10 782 females [56.4%]; median [IQR] age, 74 [71.6-77.7] years; 901 African American [4.7%], 164 Asian [0.9%], 488 Hispanic [2.6%], 17 450 White [91.3%], and 111 other race or ethnicity [0.6%]) recruited between 2010 and 2014 (median [IQR] follow-up, 4.7 [3.6-5.7] years), 16 703 participants were recruited from Australia and 2411 participants from the US (eTable in Supplement 2). A total of 9525 participants were allocated to aspirin and 9589 participants to placebo. At baseline, randomized treatment groups were well matched in demographic and risk factors for stroke.^21^

During follow-up, the rate of intracranial events including stroke was low, at 5.8 per 1000 person-years of follow-up A first stroke was experienced by 398 individuals (4.7%), including 203 individuals (4.7%) receiving placebo and 195 individuals (4.6%) receiving aspirin (hazard ratio [HR], 0.97; 95% CI, 0.79-1.18) (Table). Of these events, 53 strokes were fatal, including 22 strokes (0.5%) among individuals receiving placebo and 31 strokes (0.7%) among those receiving aspirin (HR, 1.42; 95% CI, 0.82-2.45). For comparison, there were 91 deaths from cardiovascular disease among individuals assigned to aspirin and 112 deaths among those assigned to placebo.

**Table.** Comparison of Event Rates Between Treatment Groups

Outcome	Events, No. (rate/1000 person-years)			Comparison, HR (95% CI)	*P* for treatment effect
	Overall (N = 19 114)	Placebo (n = 9589)	Aspirin (n = 9525)		
All stroke	398 (4.7)	203 (4.7)	195 (4.6)	0.97 (0.79-1.18)	.74
Nonfatal	345 (4.0)	181 (4.2)	164 (3.9)	0.91 (0.74-1.13)	
Fatal^a^	53 (0.6)	22 (0.5)	31 (0.7)	1.42 (0.82-2.45)	
Ischemic stroke	312 (3.7)	166 (3.9)	146 (3.4)	0.89 (0.71-1.11)	.28
Fatal	24 (0.3)	10 (0.2)	14 (0.3)	1.41 (0.63-3.17)	
Subtype					
Cardioembolism	59 (0.7)	34 (0.8)	25 (0.6)	0.74 (0.44-1.24)	
Large artery atherosclerosis	76 (0.9)	37 (0.9)	39 (0.9)	1.06 (0.68-1.66)	
Small vessel occlusion	81 (0.9)	46 (1.1)	35 (0.8)	0.77 (0.49-1.19)	
Other determined etiology	5 (0.1)	1 (0.0)	4 (0.1)	-	
Undetermined etiology	91 (1.1)	48 (1.1)	43 (1.0)	0.90 (0.60-1.36)	
Hemorrhagic stroke	86 (1.0)	37 (0.9)	49 (1.2)	1.33 (0.87-2.04)	.19
Fatal	29 (0.3)	12 (0.3)	17 (0.4)	1.42 (0.68-2.98)	
Subtype					
Basal ganglionic	20 (0.2)	6 (0.1)	14 (0.3)	2.35 (0.90-6.12)	
Brain stem	4 (0.0)	2 (0.0)	2 (0.0)	1.01 (0.14-7.14)	
Lobar	40 (0.5)	18 (0.4)	22 (0.5)	1.23 (0.66-2.29)	
Subarachnoid	13 (0.2)	6 (0.1)	7 (0.2)	1.17 (0.39-3.49)	
Other	9 (0.1)	5 (0.1)	4 (0.1)	0.80 (0.22-2.98)	
Nonstroke intracranial bleeding	100 (1.2)	41 (1.0)	59 (1.4)	1.45 (0.98-2.16)	.07
Fatal	22 (0.3)	10 (0.2)	12 (0.3)	1.21 (0.52-2.80)	
Subtype					
Trauma	24 (0.3)	13 (0.3)	11 (0.3)	0.86 (0.38-1.91)	
Extradural hemorrhage (traumatic)	1 (0.0)	0 (0.0)	1 (0.0)	-	
Subarachnoid hemorrhage (traumatic)	15 (0.2)	6 (0.1)	9 (0.2)	1.51 (0.54-4.25)	
Subdural hemorrhage (nontraumatic)	14 (0.2)	5 (0.1)	9 (0.2)	1.82 (0.61-5.44)	
Subdural hemorrhage (traumatic)	46 (0.5)	17 (0.4)	29 (0.7)	1.72 (0.95-3.13)	
All intracranial bleeding, including hemorrhagic stroke	187 (2.2)	79 (1.8)	108 (2.5)	1.38 (1.03-1.84)	.03
Fatal	52 (0.6)	23 (0.5)	29 (0.7)	1.27 (0.74-2.20)	

Abbreviation: HR, hazard ratio.

^a^ Fatal events were defined as events followed by death within 30 days.

### Ischemic Stroke

There were 312 participants with a first ischemic stroke (78.4% of all strokes), of which 24 strokes were fatal (10 individuals with placebo and 14 individuals with aspirin). Among participants receiving aspirin, 146 individuals (1.5%) sustained an ischemic stroke, compared with 166 individuals (1.7%) assigned to placebo. The corresponding event rate was 3.9 events per 1000 person-years with placebo and 3.4 events per 1000 person-years with aspirin, for a nonsignificant decrease of 0.5 events per 1000 person-years. Overall, aspirin did not result in a statistically significant reduction in the risk of ischemic stroke (HR, 0.89; 95% CI, 0.71-1.11).

In subgroup analysis (eFigure in Supplement 2), we found no evidence of a differential effect of aspirin across subgroups of age, sex, smoking, diabetes, dyslipidemia, frailty category, hypertension, or country (Australia or USA). The suggestive beneficial effect of aspirin in the subgroup aged 85 years and older was discounted because of small numbers (14 individuals with aspirin and 24 individuals with placebo) and a negative statistical test for heterogeneity (HR, 0.51; 95% CI, 0.25-0.99; *P* = .15).

The Table shows the frequency of ischemic stroke subtypes by treatment group. Among individuals whose subtype could be determined, small vessel occlusion was the most common, followed by large artery atherosclerosis and cardioembolism. Of 91 individuals with an undetermined etiology, this was due to incomplete evaluation, as is common in this age group, among 66 individuals (72.5%). There were no associations between aspirin and ischemic stroke subtype (Table).

### Hemorrhagic Stroke

Results for hemorrhagic stroke and intracranial bleeding based on anatomical location are displayed in the Table. Overall, there were 86 hemorrhagic strokes (21.6% of all strokes). Strokes resulting from intracerebral hemorrhage were less common than ischemic strokes, and the rate of hemorrhagic stroke recorded with aspirin (49 individuals [0.5%]) was not statistically significantly greater than that with placebo (37 individuals [0.4%]; HR, 1.33; 95% CI, 0.87-2.04; *P* = .19). There were more lobar hemorrhagic strokes (40 strokes) than other subtypes. However, the biggest differences in anatomical location of hemorrhagic stroke between individuals assigned to aspirin or placebo was within the deep perforator artery territory of the basal ganglia, although this difference was not statistically significant.

### Other Intracranial Bleeding

The difference in rates of other intracranial bleeding (Table) between individuals assigned to aspirin or placebo was not statistically significant (59 individuals [0.6%] v. 41 individuals [0.4%]; HR, 1.45; 95% CI, 0.98-2.16; *P* = .07). Subdural hematoma and subarachnoid hemorrhage, whether occurring spontaneously or as a result of trauma, were more common among individuals receiving aspirin, but the difference was not statistically significant. Overall, there were more of these subtypes of intracranial bleeding events than hemorrhagic stroke episodes (100 vs 86 events).

### Total Intracranial Bleeding

**Figure 2.**
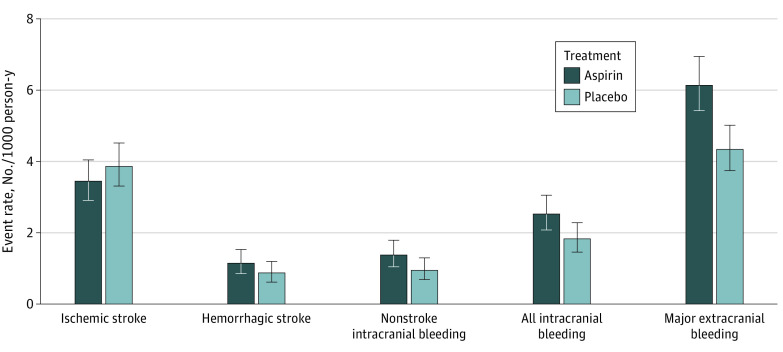
Overall Incidence Rates of Major Events Related to First Stroke and Major Bleeding Error bars indicate 95% CIs.

**Figure 3.**
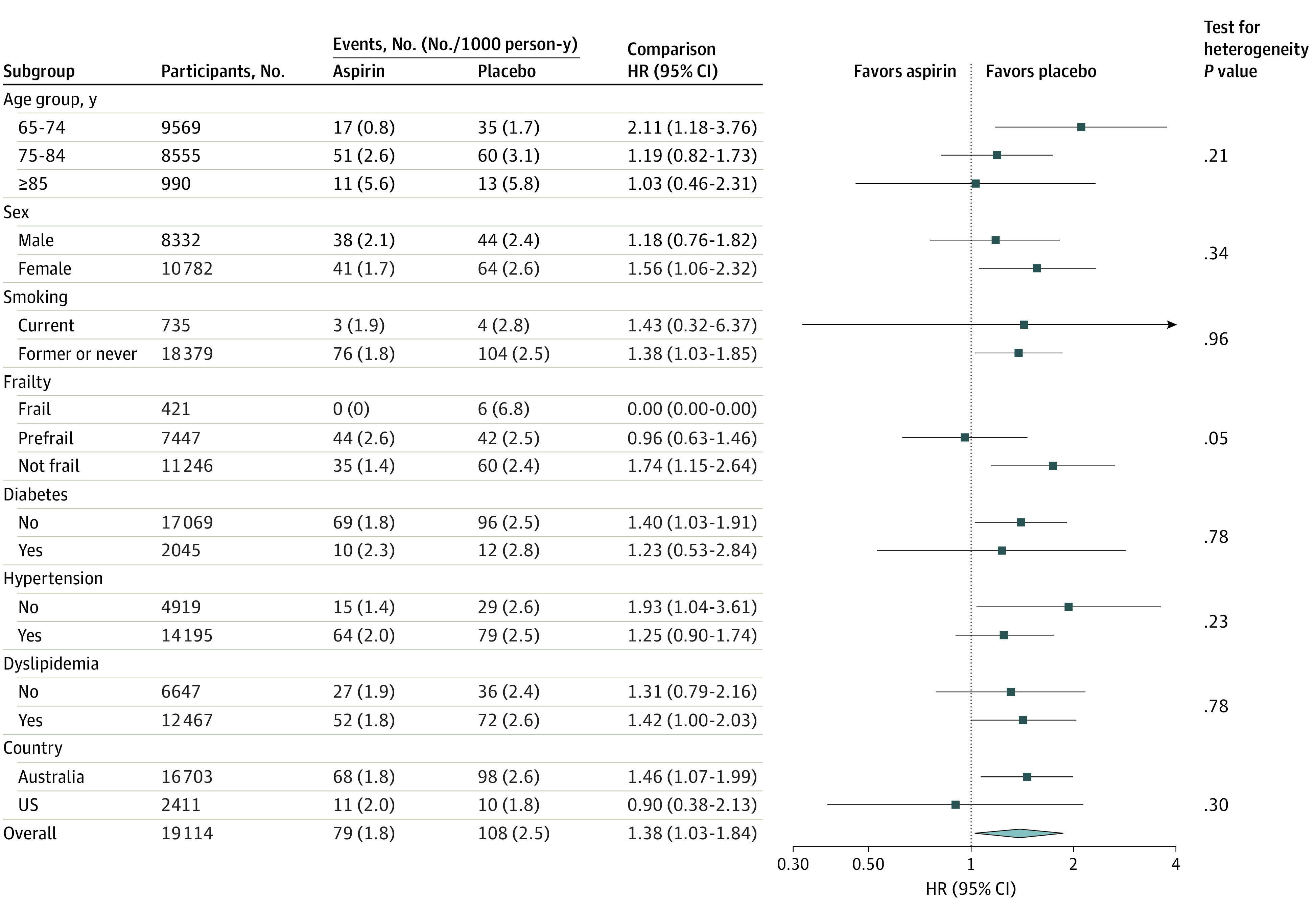
Forest Plot of First Intracranial Bleed by Subgroup HR indicates hazard ratio.

The totals of stroke and other categories of intracranial bleeding were significantly greater among individuals treated with aspirin (108 individuals [1.1%]) compared with those receiving placebo (79 individuals [0.8%]; HR, 1.38; 95% CI, 1.03-1.84; *P* = .03) (Table, Figure 2). In absolute terms, this resulted from an excess of 29 first intracranial bleeding events among individuals assigned to aspirin, which exceeded the decrease of 20 fewer ischemic strokes. As with ischemic stroke, we found no evidence of a differential effect of aspirin on the risk of having a first intracranial bleeding event across subgroups, including frailty category (Figure 3).

### Overall Stroke Prevention

**Figure 4.**
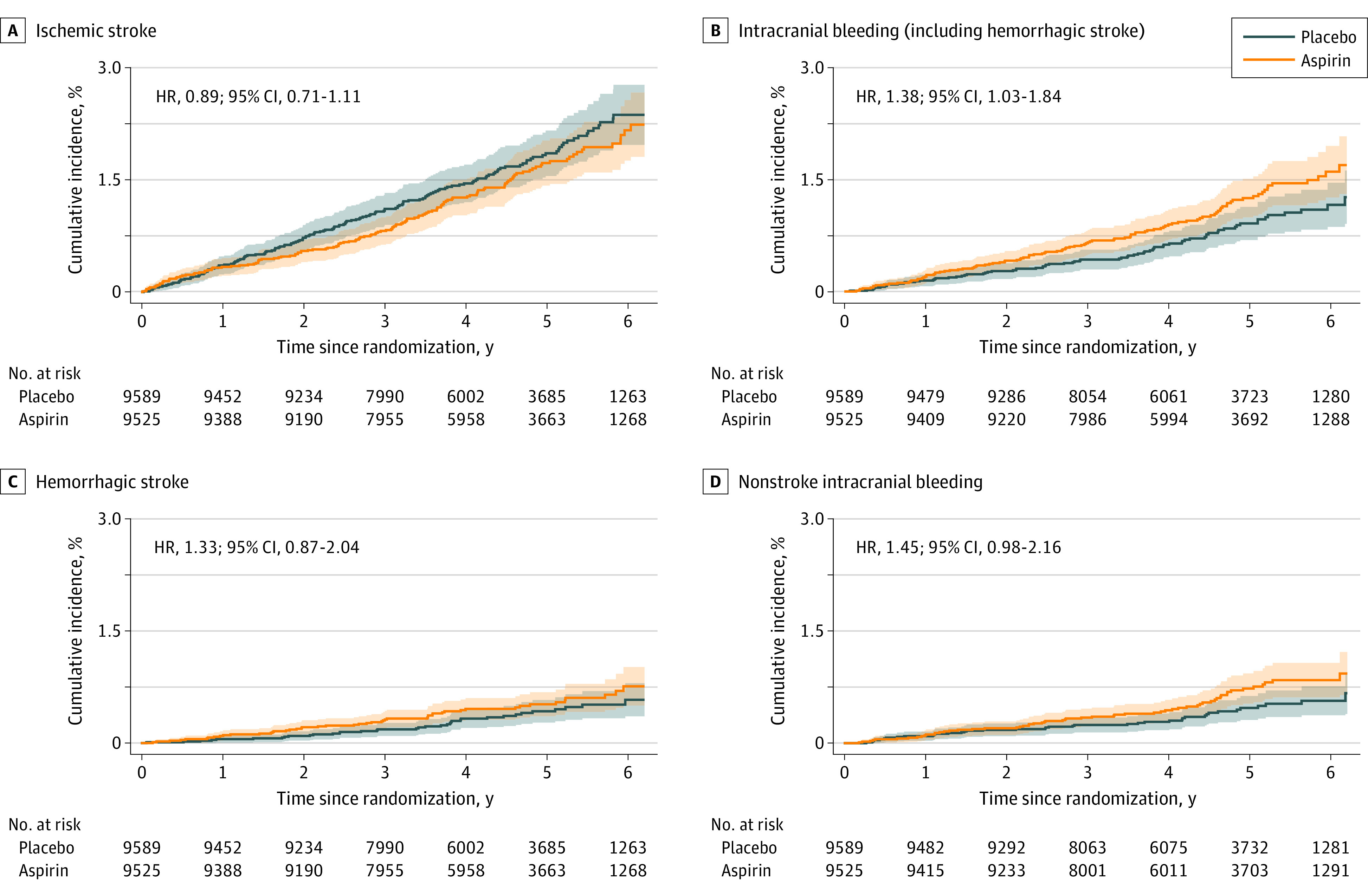
Incidence of Major Events Related to First Occurrence of Stroke and Major Bleeding HR indicates hazard ratio.

Figure 4 shows the unadjusted model of ischemic stroke and hemorrhagic stroke subtypes during the course of the study. For first ischemic stroke, the model yielded an HR of 0.89 (95% CI, 0.71-1.11) for the aspirin treatment effect. For intracranial bleeding, the corresponding HR was 1.38 (95% CI, 1.03-1.84). There was a suggestion of divergence as the follow-up continued, largely due to other nonstroke categories of intracranial bleeding (HR, 1.45; 95% CI, 0.98-2.16).

Absolute numbers of hemorrhagic and nonhemorrhagic events were small. Among 1000 individuals taking 100 mg/d of low-dose aspirin over 5 years, there were 2.5 fewer ischemic strokes at the expense of 3.5 cases of intracranial hemorrhage, a finding that did not meet the threshold for statistical significance. No difference would be expected for overall stroke incidence or stroke mortality. Major extracranial hemorrhage (Figure 2) was driven by the increased risk of upper gastrointestinal bleeding with aspirin compared with placebo, as previously found (HR, 1.87; 95% CI, 1.32-2.66).^6^

## Discussion

To our knowledge, the ASPREE randomized clinical trial is the first large-scale trial to study the risks and benefits of aspirin in an exclusively older primary prevention population, in which an increased tendency to bleeding may alter the balance of risks and benefits of aspirin. This is particularly relevant to intracerebral events because intracranial hemorrhage is typically less treatable than ischemic events and more frequently fatal or disabling.^25,26^ With previous aspirin trials in mostly younger participants, an excess of intracerebral hemorrhagic events was frequently noted among individuals receiving active treatment, although numbers were small and in most cases did not approach statistical significance.^8^

The principal finding of this secondary analysis of a randomized clinical trial was an increase in intracerebral hemorrhagic events, which in absolute terms outweighed a smaller and nonsignificant reduction in ischemic strokes. Despite the older age of the cohort, incidences of both types of events were low, with an overall rate of 5.8 per 1000 person-years of follow-up. The incidence of ischemic stroke was 0.5 incidents per 1000 person-years of follow-up lower, which was not statistically significant, while that of intracranial hemorrhage was 0.7 incidents higher, which was statistically significant.

These data extend previously reported findings on stroke outcomes in ASPREE by focusing on first stroke and bleeding events and by directly comparing the potential benefit of aspirin on ischemic stroke prevention with risks of intracranial bleeding.^6^ Findings relate to a relatively healthy older population with extensive levels of blood pressure and lipid management and without preexisting cardiovascular or cerebrovascular disease. No subgroups were identified in which the effect of aspirin was substantially different from the mean.

The lack of an effect of aspirin in reducing ischemic stroke was notable given the higher age-related risk in this population and reported efficacy of aspirin in secondary stroke prevention.^9,27,28^ Of strokes occurring in the trial, 78.4% were adjudicated as ischemic in origin, and among etiological subtypes, the largest differences between individuals assigned to aspirin or placebo were in strokes caused by small vessel occlusion (11 cases fewer) and those of presumed cardioembolic origin (9 cases fewer). However, there was little difference in ischemic strokes of large-vessel origin in which an antiplatelet like aspirin may be anticipated to be most effective. The lack of benefit in this subgroup may be due to chance with small numbers. However, the finding is in keeping with the results of an early randomized placebo-controlled trial of aspirin (325 mg/d) in 372 individuals with a 50% or greater asymptomatic internal carotid artery stenosis, which showed no evidence of benefit over 2 years of follow-up.^29^

Among individuals randomized to aspirin, there were additional cases of intracerebral, subdural, and extradural hemorrhage, some occurring after trauma and others occurring spontaneously. No subgroup was identified in which the risk of hemorrhage was substantially different from the mean. Head injury, typically resulting from falls, is common in older individuals, and additional cases of bleeding after such occurrences are an important component of the risk-benefit equation of any antiplatelet agent in older adults. Most additional cases of intracerebral hemorrhage (8/12 events) occurred in the basal ganglia, where hypertensive arteriopathy is considered to be the dominant pathology.^30,31^ Surprisingly, fewer additional cases occurred in the lobar regions, where cerebral amyloid angiopathy is thought to be the predominant underlying pathology and is common in this age group.^32,33^

Poor clinical outcomes after intracranial hemorrhagic events were reflected in higher mortality rates. Although intracerebral hemorrhage constituted 21.6% of strokes, one-third of these (29/86 strokes) were fatal compared with 7.7% of ischemic strokes (24/312 strokes). The fatality rate after subarachnoid hemorrhage and subdural hematomas was similar among individuals randomized to aspirin or placebo treatment (Table).

Overall, results are in keeping with meta-analyses reported by the Antithrombotic Trialists’ Collaboration in 2009,^9^ US Preventive Services Task Force (USPSTF) in 2016,^10^ and a 2020 meta-analysis by Judge et al^8^ summarizing results of 11 primary prevention trials, including 3 major trials published in 2018.^8^ Most patients included in these reports were younger than those in ASPREE.^8^ Consistent findings across these studies were a small reduction of ischemic events accompanied by an increase in hemorrhagic events. Unlike in our study, the absolute decrease in ischemic strokes substantially outweighed the increase in hemorrhagic events, and this has probably resulted in a discounting of intracerebral bleeding as an important component of the risk-benefit trade-off in younger populations.

The lack of benefit and potential risks from aspirin in primary stroke prevention provide further evidence in support of the recently published draft recommendation of the USPSTF against the routine prescribing of low-dose aspirin as a primary prevention measure, especially in older persons.^34^ Clinicians should be aware that among older individuals prone to falls, risks of intracerebral bleeding with aspirin may be greater than was apparent in this trial. Our results are also cautionary with regard to the inclusion of aspirin in a polypill to prevent cardiovascular disease in healthy older adults.^35,36^ Studies of newer antiplatelet therapies, such as clopidogrel, ticagrelor, or prasugrel, have not been undertaken in a primary prevention setting and should not yet be considered as alternatives to aspirin for this indication.

### Strengths and Limitations

Strengths of this data derive from its size coupled with virtually complete follow-up and systematic adjudication of stroke events by specialist clinicians. The study also has several limitations, including fewer than expected stroke and bleeding events occurring during follow-up and the lack of detailed investigation of stroke occurring in some older participants. Results are mainly generalizable to a White population (participation by individuals in racial and ethnic minority groups was largely limited to the US, where Black and Hispanic people aged ≥65 years were purposively recruited) with routine access to optimal blood pressure and lipid control. The balance of risks and benefits found in this study is not applicable to the use of aspirin for secondary prevention and may not be applicable to certain subgroups at substantially higher risk of ischemic stroke.

## Conclusions

In this secondary analysis of a randomized clinical trial of older adults, there was no statistically significant benefit from aspirin in preventing stroke or any conventional stroke etiological subtype. However, aspirin significantly increased the overall risk of intracranial bleeding. These data support the recommendation of the USPSTF that low-dose aspirin should not be prescribed for primary prevention in healthy older adults.

## Notes

Supplement 1.
Trial Protocol

Supplement 2. **eFigure.** Forest Plot for First Ischaemic Stroke by Subgroup **eTable.** Baseline Characteristics of Participants Randomized to Aspirin or Placebo

Supplement 3.
Data Sharing Statement
