# Incorrect Data in Results and Figures

**DOI:** 10.1001/jamanetworkopen.2023.40464

**Published:** 2023-10-16

The Original Investigation titled “Low-Dose Aspirin and the Risk of Stroke and Intracerebral Bleeding in Healthy Older People: Secondary Analysis of a Randomized Clinical Trial,”^1^ published July 26, 2023, contained errors in population numbers in Figure 1, data units in the Results, and column labels in Figure 3. In Figure 1, the total population assessed for eligibility should have read 23 163, and 276 participants were excluded from the placebo group after randomization. In the second paragraph of the Results, the units should have been per 1000 person-years. In Figure 3, the headers “Aspirin” and “Placebo” were switched. This article has been corrected. The corrections do not alter the results or conclusions of the study.^1^
