# Supplementary material for: Low-Dose Aspirin and the Risk of Stroke and Intracerebral Bleeding in Healthy Older People: Secondary Analysis of a Randomized Clinical Trial
Source: JAMA Netw Open. 2023 Jul 26;6(7):e2325803. doi: 10.1001/jamanetworkopen.2023.25803 (PMC10372701; doi:10.1001/jamanetworkopen.2023.25803)
Supplement: Supplement 1. — Trial Protocol [file jamanetwopen-e2325803-s001.pdf]

ASPREE

ASPirin in Reducing Events in the Elderly

Protocol

1 INTRODUCTION 5

1.1 Summary 5
1.2 Background 5
1.2.1 Major adverse cardiovascular events 6
1.2.2 Cardiovascular benefit versus risk in the elderly 7
1.2.3 All-cause dementia 8
1.2.4 Physical disability 9
1.2.5 Cancer 10
1.2.6 Depression 11
1.2.7 Serious hemorrhagic events 11
1.2.8 Study Population – Australia and the U.S.: gender, age and minorities 13
1.2.9 Significance for medical practice 14
1.2.10 Summary of rationale and significance 15

2 STUDY DESIGN 16

3 OBJECTIVES 16

3.1 Primary Objective 16
3.2 Secondary Objectives 16
3.3 Other Measures 16

4 HYPOTHESIS 17

5 ENDPOINTS 17

5.1 Primary Endpoint 17
5.2 Secondary Endpoints 17
5.3 Other Measures 18
5.4 Endpoint Ascertainment 18
5.5 Endpoint Adjudication Process 19

6 STUDY POPULATION 20

6.1 Inclusion criteria 20
6.2 Exclusion criteria 20
6.3 Participant Recruitment 21
6.3.1 General Practice recruitment (in Australia) 21
6.3.2 Community-based recruitment (in the U.S.) 22
6.4 Participant discontinuation 23
6.5 Contingency plans for participant well-being 24
6.5.1 Follow up of participants 'off protocol' 24
6.5.2 Anemia diagnosed in a study participant 24
6.5.3 Major surgery 25
6.5.4 Major bleed 25
6.5.5 Abdominal discomfort/dyspepsia 25
6.5.6 Commencement of regular non-steroidal medication (NSAID) 26
6.5.7 Development of an indication for aspirin 26
6.5.8 New diagnosis of diabetes 26
6.5.9 New diagnosis of atrial fibrillation 26
6.5.10 Commencement of warfarin or other antiplatelet drugs 26
6.5.11 Commencement of aspirin on own volition 27

7 MEASUREMENTS, CRFS AND ANALYTICAL METHODS 27

7.1 Schedule of study visits 27
7.1.1 Lifestyle Profile and Screening visit (Visit 1) 27

7.1.2 Post screening visit study activity (Run-in phase between Visits 1 & 2) *29*
7.1.3 Determinations at Eligibility and Assessments visit (Visit 2) *30*
7.1.4 Randomization Procedure *31*
7.1.5 Annual Visits *31*
7.1.6 Participant Retention Plan *32*
7.1.7 Telephone Contact *32*
7.1.8 Other Actions *33*
7.2 Case Report Forms (CRFs) 33
7.3 Data Analysis 33
7.3.1 Interim analysis *34*
7.3.2 Final analysis *34*
7.3.3 Further details for statistical analyses *34*
7.3.4 Pre-specified sub-group analyses *35*
7.4 Dissemination and implementation of results 36
7.4.1 Objectives *36*
7.4.2 Approach and methods *36*

8 STUDY MEDICATION AND SUPPLIES 37

8.1 Study treatments 37
8.2 Drug packaging 37
8.3 Administration of study medication 38
8.4 Dispensing and randomization 38
8.5 Drug storage 39
8.6 Drug accountability and dispensing logs 39
8.7 Drug disposal 40
8.8 Emergency code breaking 40
8.9 Overdose of trial medication 40

9 CONCOMITANT THERAPY 41

9.1 Prescription medications 41

10 SAMPLE SIZE AND POWER CALCULATIONS 41

11 ASSESSMENT AND REPORTING OF ADVERSE EVENTS 42

11.1 Adverse Events 42
11.2 Serious Adverse Events (SAE) 42

12 ADHERENCE TO ETHICAL, REGULATORY AND ADMINISTRATIVE CONSIDERATIONS 43

12.1 Ethical Considerations 43
12.1.1 General *43*
12.1.2 Information for Participants *44*
12.1.3 Informed Consent *44*
12.2 Regulatory Considerations 44
12.2.1 Financing *44*
12.2.2 Trial registration *45*
12.2.3 Disclosure of Conflict of Interest *45*
12.3 Administrative Organization 45
12.3.1 Individual and Committee responsibilities *45*
12.3.2 Data Safety and Monitoring Plan *47*
12.3.3 Publication policy *47*
12.3.4 Ancillary studies *48*

13 DATA MANAGEMENT 48

13.1 DATA HANDLING AND RECORD KEEPING 48
13.2 QUALITY CONTROL 49
14 REFERENCES 51
APPENDIX 1. ENDPOINTS AND OTHER MEASURES 59
APPENDIX 2. SAMPLE SIZE & POWER CALCULATIONS 71
APPENDIX 3 CONFLICT OF INTEREST 76
APPENDIX 4. DSMB CHARTER 78
APPENDIX 5. ACES (ASPREE CANCER ENDPOINTS STUDY) TUMOR TISSUE BANKING.....

1 INTRODUCTION

1.1 Summary

ASPREE is a double-blind, randomized, placebo-controlled primary prevention trial designed to assess whether daily active treatment of 100 mg enteric-coated aspirin will extend the duration of disability-free life in healthy participants aged 70 years and above except for Hispanic and African American minority groups in the U.S. where the minimum age of entry is 65 years. The study will examine whether the potential benefits of low dose aspirin (particularly the prevention of heart disease, stroke, certain cancers and dementia) outweigh the risks (particularly severe gastrointestinal bleeding and hemorrhagic stroke) in this age group.

Participants will be eligible for the trial if they do not have a current clinical indication for (i.e. overt cardiovascular disease) or contraindication to (i.e. allergy or increased risk of bleeding) aspirin, do not have dementia, disability, low hemoglobin levels, or have a condition that is likely to be fatal during the 5 years of the trial and are capable of providing informed consent. Nineteen thousand participants will be required to provide 90% power of a true relative risk benefit of 0.90 for the primary endpoint (a composite of all-cause mortality, incident dementia and persistent physical disability) in an intention-to-treat analysis with an average follow-up of 5 years. The trial has received financial support from the U.S. National Institute on Aging (part of the National Institutes of Health), the National Health and Medical Research Council of Australia, the National Heart Foundation of Australia and the Victorian Cancer Agency. Bayer Pharma AG provides in kind support through the provision of low dose aspirin and matching placebo.

The study will be carried out in community settings in the U.S. and Australia. In the U.S., 2,500 participants will be recruited through clinical trials networks in regional hub settings. In Australia, recruitment of 16,500 participants will take place through general practices with the participant's usual treating General Practitioner (GP) as co-investigator.

1.2 Background

Low dose aspirin therapy has been shown to reduce the risk of vascular events in a wide range of primary and secondary care settings, largely in middle-aged people.[1-3] There is also some evidence of its potential to reduce the rate of intellectual decline and certain malignancies in older participants.[4] Part of the benefit of aspirin may be offset by adverse effects, principally those related to its potential to cause bleeding. The balance of risks and benefits of low dose aspirin has not been established in older persons. Three recent editorials have identified the

need for the ASPREE study to be conducted. [5 6 7] Each highlighted the need for prospective data to resolve the current uncertainty regarding the effects of aspirin amongst older persons.

Previous studies on the effects of aspirin in primary prevention have mainly focused on cardiovascular outcomes. In the elderly, these alone may not be the most appropriate measure of benefit associated with aspirin treatment. Prolongation of life free of functional disability in a healthy aging population would be the most desirable benefit of a pharmacological strategy focusing on prevention. Underlying causes of death and disability in an aging population include major cardiovascular events (such as stroke, myocardial infarction [MI] and heart failure), dementia and cancer. An altered incidence of any (or all) of these might contribute to the overall balance of risks and benefits of aspirin therapy in this age group.

1.2.1 Major adverse cardiovascular events

Cardiovascular and cerebrovascular events are the primary cause of death in older adults but they are also major determinants of mental [8] and chronic physical disability. [9] The effects of low-dose aspirin on cardiovascular outcomes have been tested in primary and secondary prevention. Meta-analyses of these studies, undertaken by the U.S. Preventive Services Task Force and the Anti-Thrombotic Trialists' (ATT) Collaboration, have shown that in secondary prevention aspirin therapy reduces the subsequent incidence of cardiovascular death, non-fatal MI and non-fatal stroke by approximately 25%. [10 11] The relative risk reduction was similar amongst subgroups based on age, sex, diabetes or underlying disease (i.e. angina, MI, transient ischemic attack (TIA), stroke or peripheral arterial disease (PAD)). Similar risk reduction was observed within a dose range spanning from 75 to 1500 mg/day although doses less than 75 mg appeared less effective.

In primary prevention, the available data has been drawn from six major morbidity/mortality Randomized Controlled Trials (RCTs), whose participants were predominantly middle-aged (in the 50-70 years age group) and at a low cardiovascular risk.[12-17] Meta-analysis of five of these studies, undertaken primarily in men (>80%), has shown a significant reduction in vascular events, resulting principally from a one third reduction in first MI.[18] There was no significant reduction in stroke, although with the relatively young age of the participants, relatively few cerebrovascular events were recorded.

Five of these six major primary prevention trials were undertaken amongst low risk individuals (10 year cardiovascular risks were 2.5%, 3.6%, 4.8%, 8.9% and 12.4%, respectively). Modeling of the results suggests that within the age range of 50-70 years, and at this level of risk, the

'gains and losses' are finely balanced. For example, Hayden has estimated that for 1000 patients with a 5% cardiovascular risk over 5 years, treatment throughout this period would prevent 6-20 cases of MI, at a cost of 0-2 hemorrhagic strokes and 4 major gastrointestinal events.[10] For patients with a 1% cardiovascular 5-year risk (similar to the Women's Health Study – WHS [16]) aspirin would prevent 1-4 cases of MI but would again cause 0-2 hemorrhagic strokes and 2-4 major bleeds.

In 2005 more detailed information became available about the value of aspirin for primary prevention in women. The WHS involved 39,876 apparently healthy women over 45 years of age who were randomized to 100mg aspirin on alternate days and followed for 10 years.[16] Results were a 17% reduction in the risk of stroke (RR 0.83, 95% CI: 0.69-0.99) but no effect on MI (RR 1.02, 95% CI: 0.84-1.25), i.e. the opposite pattern to that seen in men. These findings have since been substantiated by a gender sub-group analysis by Berger [19] who reported that the benefit of aspirin for the primary prevention of major adverse cardiovascular events was driven by acute MI risk reduction in men and thromboembolic stroke risk reduction in women.

The WHS [16] also included a subgroup analysis by age and reported that: *"An interesting finding in our subgroup analyses was that the most consistent benefit of aspirin was observed among women 65 years of age or older. This group of 4097 women composed 10 percent of the study population yet had almost one third of the cardiovascular events. In this group, aspirin use, as compared with placebo use, led to 44 fewer myocardial infarctions, strokes, or deaths from cardiovascular causes (P=0.008) but to 16 more gastrointestinal hemorrhages requiring transfusion (P=0.05), emphasizing, as with any agent, the importance of balancing benefits and risks."*

1.2.2 Cardiovascular benefit versus risk in the elderly

A meta-analysis by the ATT [20] of all six primary prevention trials by age demonstrates that the effect of aspirin on all-cause mortality has been highly variable ranging from a 3% increase to a 19% decrease.

If the proportional benefit and rate of adverse events in older people were similar to those seen in middle-aged people, the modeling might be expected to demonstrate a substantial advantage for aspirin treatment as a result of the higher absolute risk in this group. However, in light of the very limited trial data available for the over 70 age group, and with evidence suggesting a substantially greater rate of adverse events in that age group, such an outcome cannot be assumed. This concern is supported by epidemiological modeling that suggests the benefits of

using aspirin routinely for the primary prevention of cardiovascular disease (a reduction of incident MI and ischemic stroke) in those aged 70 years or more may be offset by increased cases of serious bleeding.[21] This uncertainty warrants the true balance of risks and benefits for these and other outcomes in older persons to be established by a RCT in sufficient participants to accurately weigh these possibilities and to investigate impacts on other diseases prevalent in older people. In further support for the need for a trial such as ASPREE, a meeting in December 2003 of the FDA Cardiovascular and Renal Drugs Committee voted 11-3 against the expansion of aspirin professional labeling to include moderate-risk individuals for the primary prevention of MI.[22] The proposed composite endpoint will be an integrated result of the risks as well as the benefits of aspirin treatment.

1.2.3 All-cause dementia

There is preliminary, albeit conflicting, evidence to suggest that aspirin may be effective in delaying the onset of vascular dementia, a major cause of cognitive decline in older people.[23] Similar evidence is accumulating to suggest that non-steroidal anti-inflammatory drugs (NSAIDs) may also be protective.[24-26] However, concerns about the long-term cardiovascular safety of these drugs support the proposal that aspirin alone amongst this group of drugs has the potential to be used as a preventive agent in this age group.[27 28] While aspirin's main clinical effect is likely to be via its anti-platelet action (preventing micro-infarcts and stroke), it is possible that it may also produce a dose-dependent anti-inflammatory effect and through this mechanism delay the onset of Alzheimer’s disease.[29 30] For a disease with no known curative or preventive treatment, the public health benefits of delaying onset could be highly cost effective. Brookmeyer *et al*. reported that the potential effects of an intervention (e.g. aspirin) that delayed the onset of Alzheimer’s dementia by 2 years would reduce the expected prevalence of the disease in the U.S. by 1.94 million cases after 4 years.[31]

Therapeutic measures to prevent the onset of dementia, as opposed to treating the progression of established dementia, may be best targeted towards the early stages of cognitive impairment. A recently published report from the Aspirin for Asymptomatic Atherosclerosis (AAA) study, which is a placebo-controlled trial of low dose aspirin in those with asymptomatic PAD with 5 years of follow-up, failed to show any benefit from daily 100 mg aspirin in reducing cognitive decline.[32] An accompanying editorial in the *British Medical Journal* outlined the reasons why this study may have failed to find an effect of aspirin and, indeed, highlighted the importance of the ASPREE study to determine whether aspirin can prevent cognitive dysfunction.[7] A meta-analysis of four observational cohort studies in healthy older people, where cognitive decline was the

endpoint, reported no apparent benefit of NSAID treatment.[33] A more recent report from the WHS cognitive cohort also found no benefit of low dose aspirin (100 mg on alternate days) *versus* placebo in preventing cognitive decline.[34] However, the low dosage used, the mean age of 72 years at the first cognitive function measurement (at the younger end of the 'elderly' over 70s) and the insensitivity of the phone-administered tests that were used to assess higher executive functions suggest the need for a further study without these limitations. An accompanying editorial called for 'better quality research into cognitive decline in later life'.[35]

ASPREE will be the first prospectively planned placebo-controlled trial of aspirin therapy undertaken amongst individuals within the age group of 75+ years where cognitive decline is more common and where an effective intervention is most likely to be recognized. It will incorporate a detailed assessment of cognitive impairment and dementia onset amongst older participants of both genders.

1.2.4 Physical disability

While prolongation of life remains an important public health goal, of even greater significance is that extended life should involve preservation of the capacity to live independently and to function well. Therefore, identification of proven interventions to prevent disability is a major public health challenge. Mobility and activities of daily living represent tasks that are necessary for the maintenance of basic independent functioning.[36 37] The inability to perform these activities marks a serious decline in functional health, conferring increased risk of institutionalization and death. Because of its public health significance, we will assess ADL (Activities of Daily Living) disability as part of the primary endpoint of this trial and also alone as a secondary endpoint. Because cognitive impairment alters self-perception of function, we will request this information from a proxy in all participants in whom the dementia evaluation is triggered.

Aspirin has the potential to decrease the risk of physical function decline by reducing the risk of sub-clinical and clinical stroke, MI and PAD. Subclinical strokes, MIs and PAD as assessed by brain MRI, echo and ECG, and by ankle:brachial index respectively, are all associated with impairment and decline in gait speed, hand grip strength and cognition.[38-40] In the Cardiovascular Health Study (CHS), the incidence of ADL difficulty by the presence or absence of brain infarct on MRI was 8.6 per 100 person-years, compared to 6.7 per 100 person-years, and gait speed was related to subclinical brain MRI changes, but independent of clinical strokes over the time period.[40]

We will also measure performance in gait speed and grip strength as tertiary outcomes to

capture more subtle effects of aspirin on early decline. These performance measures are most closely linked both to CVD [40] and to mortality [41-44] and capture most of the predictive information relative to more complex measures. [45]

1.2.5 Cancer

The possibility that aspirin may have a role in the prevention of cancer has been raised by a number of clinical trials and observational studies. In 2005, a systematic review of data from 91 observational studies showed a statistically significant exponential decline in risk with increasing NSAID dose (primarily aspirin or ibuprofen) for 7 of 10 malignancies.[46] Daily intakes of NSAIDs (325 mg aspirin or 200 mg ibuprofen) were associated with risk reductions of 63% for colon cancer, 39% for breast, 36% for lung and 39% for prostate cancer. These effects became evident after five or more years of use and increased with increasing duration of therapy. Similar conclusions were drawn from a 2008 meta-analysis regarding aspirin and NSAID use for breast cancer prevention, although the risk reduction was closer to 20%.[47] More recently, a 10 year follow-up of participants in the Iowa Women's Health Study (IWHS) found that regular aspirin use was associated with a 16% reduction in incident cancers from any site, but that other NSAIDs caused no reduction.[48] By contrast the WHS, a randomized placebo-controlled trial, showed no overall benefit for very low-dose aspirin (100mg on alternate days) over placebo for newly diagnosed invasive cancer at any site.[49]

Evidence of aspirin's action is particularly strong for colorectal cancer, the third most frequently occurring cancer in Caucasian men and women.[50] The risk of this malignancy increases rapidly with age, with the highest incidence found in people aged 85 and over.[50 51] Two RCTs have demonstrated that aspirin can reduce the incidence of new adenoma development in individuals with a history of bowel cancer or previous colon adenomas.[52 53] Investigators from the Nurses Health Study (NHS) reported a relative risk reduction in colorectal cancer incidence of 0.77 (95% CI: 0.67-0.88) for women classified as "regular" aspirin users (≥2 x 325 mg tablets / week),[54] which is within the dosage range of aspirin use for cardiovascular disease prevention.

More recently, Chan and colleagues[55] reported that regular aspirin use was associated with a 36% decrease in the incidence of colorectal cancer amongst 130,000 health professionals followed in longitudinal cohort studies in the U.S. This effect was seen only amongst the 67% of colorectal malignancies that over-expressed the COX-2 enzyme. Flossman and Rothwell found in a recent meta-analysis of two U.K. RCTs that regular use of aspirin was associated with a 26% lower risk of colorectal cancer incidence, but the effect required a combination of high dose

and a prolonged duration of treatment (5 years) and follow-up (10 years) to become statistically significant.[56] This provides a possible explanation as to why two U.S. trials (the Physician's Health Study and the WHS) failed to identify a similar effect.[49 57]

The U.S. Preventive Services Task Force recently concluded that on present evidence, the harms of aspirin use outweigh the benefits for the prevention of colorectal cancer.[49 51 54 57] However, the overall trend in data suggests that a RCT in older participants is needed to resolve the question of whether regular low-dose aspirin use is preventive for colorectal malignancy in those aged 70 and above. In this context, a sub-group analysis of women aged 65 - 74 years within the NHS cohort revealed a 22% decrease in the relative risk of (all) cancer mortality with regular aspirin use.[58]

Although the evidence for a chemoprotective role for aspirin against malignancies is currently strongest for colorectal cancer, the recent review in *Nature Reviews / Cancer* [59] emphasized that *"…epidemiological evidence is accumulating that aspirin or NSAID use is protective against esophageal and gastric cancer, and possibly also against cancers of the prostates, ovary and lung. Recently, an inverse association has been reported between aspirin use and breast cancer."* Ulrich and colleagues also point out that randomized controlled trials for primary (and secondary) prevention are needed to determine whether aspirin use reduces the risk of these malignancies. [59]

1.2.6 Depression

Depression is a common accompaniment of aging. Estimates of the prevalence of depression in the elderly range from 10-15% including major depressive disorder, minor depression and dysthymic disorder.[60 61] Depression in the elderly is associated with a wide range of adverse outcomes, including increased mortality, suicide, increased dementia and substantial psychosocial disability.[61 62]

Over recent years, elevated levels of inflammatory biomarkers have been identified in patients with clinical depression.[63] The raised biomarkers include C-reactive protein (CRP), Interleukin-6 (IL-6) and TNF-alpha, and elevated levels of these biomarkers may predict the onset of depression.[64]

Aspirin reduces levels of key inflammatory biomarkers. [65] If the incidence of new onset depression is less in the aspirin treated arm than in the placebo arm of ASPREE, this may indicate a potential role for aspirin in the prevention and/or treatment of depression in older patients.

1.2.7 *Serious hemorrhagic events*

The potential risks of aspirin therapy may be greater in older people compared with the more extensively studied middle-aged. The recent WHS reported significant increased gastrointestinal bleeding events with aspirin in the participant population overall and, of note, in the subgroup aged 65 years or more.[16] Cohort data suggests a higher absolute risk of gastrointestinal hemorrhage amongst older participants, and if so it is likely that lesser degrees of bleeding will also be more common (Figure 1).[66] This latter concern was demonstrated in both PACE [67] and ASPREE [68] pilot studies, where there was an average hemoglobin reduction of 0.2 gm% in those receiving low-dose aspirin compared to placebo. ASPREE will provide an opportunity to investigate whether such findings are limited to the first year of treatment or are an ongoing trend.

There is also residual uncertainty about the extent of the risk of intracerebral hemorrhage in older people with low-dose aspirin therapy.[69 70] The risk of intracerebral hemorrhage generally is substantially higher in older persons; the impact of this risk may therefore be magnified with regular low-dose aspirin intake.[71]

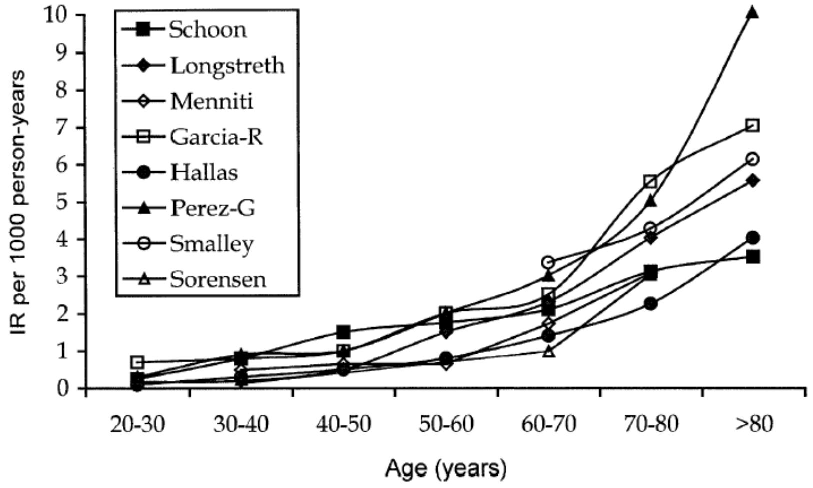

Figure 1. Incident rates (IR) of serious upper gastrointestinal complications per thousand person-years by age. Reproduced from Hernandez-Diaz and Garcia Rodriguez.[66]

Distinction between ischemic stroke and hemorrhagic stroke can not be accomplished reliably without CT scanning. In both Australia and the U.S. such imaging is undertaken in virtually all cases of suspected stroke and results will be sought for presentation to the Endpoint Adjudication Committee (EAC). This will allow ischemic and hemorrhagic stroke to be determined separately during the course of ASPREE.

1.2.8 Study Population – Australia and the U.S.: gender, age and minorities

Both the U.S. and Australian seniors' populations are multicultural but with a predominance of white skinned people of European origin. Life expectancy data from the WHO suggests similar life expectancy profiles for men and women amongst the older population of both nations (Figure 2). Age-standardized death rates also suggest similar prevalence of the major cardiovascular diseases in both genders (Table 1).

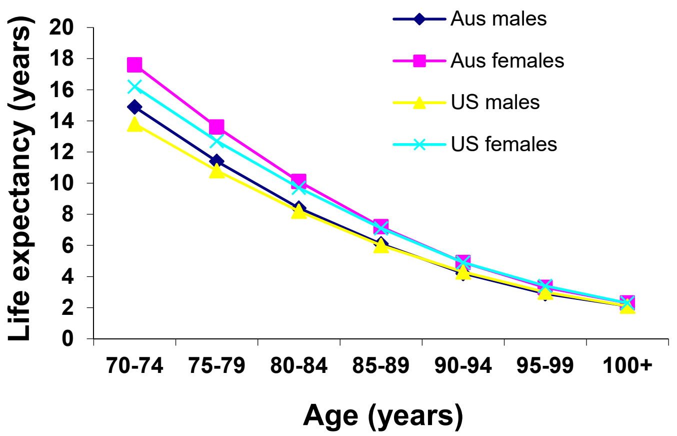

Figure 2. A comparison of US and Australian life tables 2006 (http://www.who.int/whois/database/life_tables/life_tables.cfm).

Among U.S. minorities, life expectancy at 75 years for African Americans is only slightly lower than for whites. For U.S. Hispanics, life expectancy rates from birth are intermediate between those of whites and African Americans (Table 2). In the U.S., age–adjusted all-cause death rates differ by race: for African Americans the death rate is 1016.5, for whites 796.6 and for Hispanics 785.3 per 100,000 (2005). Cardiovascular mortality rates per 100,000 exhibit a similar pattern; for African Americans it is 271.3, for whites 207.8, and for Hispanics, 157.3. [72]

In Australia, only 2.5% of the population is indigenous and of these, 25% live in remote isolated communities. Life expectancy is ~17 years less and CVD more prevalent in the aboriginal community than for the non-indigenous population. We estimate that only ~500 indigenous Australians could be eligible and available for the ASPREE study.

Condition	U.S. males (2002)		U.S. females (2002)		Aus males (2003)		Aus females (2003)	
**Age Group**	65-84	85+	65-84	85+	65-84	85+	65-84	85+
**All cause**	3832	16255	3312	14210	3487	15937	2291	13259
**CVD***	1759	7142	1180	6785	980	5746	655	5280
**Malignancies**	1152	2491	848	1391	1238	2893	715	1535

Table 1. Death rates per 100,000 population by age for all-cause mortality, major cardiovascular disease (CVD; heart and cerebrovascular) and cancer. Sources: 2002 US data [72] and 2003 Australian data [73]. * CVD rates for US males and females are higher than that identified in Australia. This is primarily due to the differences in definitions of CVD used in these surveys and ethnic differences in populations where the Australian population is primarily Caucasian.

	AA males	AA females	White males	White females	Hispanic males	Hispanic females
**Life expectancy at age 75+ yrs (2005)** [72]	10.0	12.3	10.8	12.8	NA	NA
**Life expectancy from birth (1999)** [74]	68.5	75.1	84.8	89.0	77.2	83.7

Table 2. **U.S. Minority life expectancy rates (AA = African Americans)**

1.2.9 Significance for medical practice

ASPREE will likely have a significant impact on medical care world-wide because:

(a) The aging of the U.S. and Australian populations will progressively increase the numbers for whom these results are applicable;

(b) Methods of effectively delaying the onset of chronic diseases associated with aging are a high priority for preventive medicine;

(c) Aspirin is an affordable medication whose use is unlikely to be limited by income or insurance status.

The ASPREE population sample will be sourced from the U.S. and Australian communities, providing broad representation from the aged community. Both rural and urban areas will contribute participants to the study. Participants of both genders and from different ethnic backgrounds will be included.

1.2.10 Summary of rationale and significance

- Low-dose enteric aspirin is potentially one of the most effective preventive agents for use in older people. It has the potential to extend the duration of the healthy active life of older people by delaying the onset of cardiovascular disease (CVD), stroke and possibly dementia. These are the most common causes of physical disability and hospitalization amongst the senior U.S. and Australian populations. If it proved effective, the low cost of aspirin therapy would make it an affordable preventive agent for older populations in all countries.
- Despite its potential benefits, there is limited data available about aspirin's effectiveness in older persons free of clinically overt vascular disease. This is because presently available primary prevention trials of low-dose aspirin have largely been undertaken in younger populations. Equivocal results in such trials have led to a generally low use of aspirin for primary prevention at all ages in some countries, particularly Australia. In the U.S. major guidelines have advocated low-dose aspirin therapy despite the FDA's denial of a labeling that would endorse such use.
- The higher absolute risk of vascular disease in older people may lead to a substantially more favorable benefit-to-risk ratio in older people than in the young. However, it is also possible that a higher incidence of adverse events (especially serious bleeding and the consequences of anemia) may negate this benefit. The higher burden of adverse effects in older persons has been documented but it is not clear whether these are sufficient to negate the clinical benefits.
- Concerns about the cardiac safety of non-steroidal anti-inflammatory agents (including both selective and non-selective COX inhibitors) have returned the focus of preventive and therapeutic attention to aspirin. [28] Use of aspirin is therefore likely to rise in the ASPREE age group and the imperative to provide evidence for this group is increased. A specific trial of low-dose aspirin in older participants as envisaged in this study has been advocated in several recent review articles and editorials.
- With the numbers of seniors expected to rise sharply over the coming decades, development of new, safe effective ways of delaying the onset of the major disabling chronic diseases is a high priority for medical research. Low-dose aspirin therapy is a principal candidate because of its potential to delay the most common and disabling of these conditions. Confidence in its use will require a study to demonstrate unequivocally that its benefits outweigh its risks. The history of long-term medication use in otherwise healthy individuals must be supported by adequate data on safety.

2 STUDY DESIGN

ASPREE is a randomized, double blind, placebo-controlled trial. Participants will be randomized remotely via the ASPREE Data Management Center web portal. For both systems, password protected access is required for study personnel. According to a computer-generated randomization schedule, participants will be allocated to 100 mg enteric coated aspirin or placebo in a ratio of 1:1. Randomization will be stratified for general practice in Australia, for regional site in the U.S. and for age (65-79 or ≥ 80 years).

During an initial 4 week run-in phase participants will take placebo for compliance checking. Bayer Pharma AG will supply active drug and matching placebo.

Pre-screening	Lifestyle Profile & Screening (Visit 1)	Run-in phase & GP visit*	Assess-ments & Eligibility (Visit 2)	Annual Visit (1)	Annual Visit (2)	Annual Visit (3)	Annual Visit (4)	Annual Visit (5)	Annual Visit (6)	Annual Visit (7)
	↓	↓	↓	↓	↓	↓	↓	↓	↓	↓
**Time**	**-4 weeks**		**0**	**Year 1**	**Year 2**	**Year 3**	**Year 4**	**Year 5**	**Year 6**	**Year 7**
	**There will be additional 3-month, 6-month and 9-month telephone contact (between randomization and annual visits.**									

Table 3. Timeline for ASPREE. *GP visit applies in Australia. Annual visits will be for 3-7 years, depending on year of randomization.

3 OBJECTIVES

3.1 Primary Objective

The primary objective is to determine whether low-dose aspirin prolongs life, or life free of dementia, or life free of significant, persistent physical disability in the healthy elderly.

3.2 Secondary Objectives

Secondary objectives relate to the effects of low-dose aspirin on the key outcome areas of death, cardiovascular disease, dementia and cognitive decline, cancer, physical disability, depression and major bleeding episodes – see Section 5.2.

3.3 Other Measures

Measures in addition to those included in the primary and secondary objectives will be examined. These include measures such as hemoglobin levels, urine albumin:creatinine ratios, tests of mental and physical function and hospitalizations – see Section 5.3.

4 HYPOTHESIS

Null hypothesis: Daily 100 mg enteric-coated aspirin will have no benefit over placebo in prolonging life, or life free of dementia or life free of significant physical disability in healthy participants aged 70 years and over.

Alternative hypothesis: Daily 100 mg enteric-coated aspirin prolongs life, or life free of dementia or life free of significant physical disability in healthy participants aged 70 years and over.

5 ENDPOINTS

Clinical and ancillary evidence must be supplied by the investigator to support all diagnoses. Major adverse cardiovascular, but not dementia, endpoints lead to withdrawal from 'on randomized treatment' but not from intention-to-treat follow-up. Participants who reach study endpoints for dementia or disability will be followed for the duration of the trial for fatality.

Detailed definitions of the endpoints and other measures are provided in Appendix 1: Endpoints and Other Measures.

5.1 Primary Endpoint

The primary endpoint is death from any cause or incident dementia or persistent physical disability. Dementia will be diagnosed based on DSM-IV criteria. Significant physical disability will be defined as a confirmed, and persisting for at least 6 months, self-report of 'a lot of difficulty', or 'inability to perform independently' any one of the 6 Katz basic Activities of Daily Living (ADLs).[75]

5.2 Secondary Endpoints

Secondary endpoints include:

- All-cause mortality
- Fatal and non-fatal cardiovascular events including a) coronary heart disease death, b) non-fatal MI, c) fatal and non-fatal stroke, and d) any hospitalization for heart failure
- Fatal and non-fatal cancer, excluding non-melanoma skin cancer
- Dementia
- Mild Cognitive Impairment (MCI; assessed using the Modified Mini-Mental State Examination or 3MS [76] and other cognitive function measures – see below)
- Physical disability
- Depression; assessed using the CES-D (Center for Epidemiologic Studies –

Depression)-10 [77] questionnaire, or hospitalization for depression

- Major hemorrhagic events

These secondary endpoints represent major individual components that may contribute to the primary endpoint of the ASPREE study. Each of the endpoints has objective ascertainment criteria (outlined in Appendix 1) and each will be reviewed by the Endpoint Adjudication Committee (EAC). The cardiovascular and cancer endpoints represent the major expected causes of mortality in the study population, whilst dementia, cognitive decline, physical disability and depression impact on the individuals' ability to participate in healthy active life. Hemorrhage is an important secondary endpoint contributing to the evaluation of the risk versus benefits of aspirin therapy in this population.

5.3 Other Measures

These outcomes include:

- Additional cognitive function measures (Symbol-Digit Modalities Test (SDMT) [78], Hopkins Verbal Learning Test-Revised or HVLT-R [79], Controlled Oral Word Association Test or COWAT [80])
- Physical function measures: performance-based (including gait speed and hand grip tests) and self-reported (LIFE questionnaire [81])
- Quality of life (Short Form – 12 or SF-12 [82])
- Hemoglobin levels
- Urine albumin : creatinine ratio for the detection of microalbuminurea
- Hospitalization (for reasons other than primary or secondary endpoints)

5.4 Endpoint Ascertainment

At each annual visit and 6 month telephone contact, the participant will be questioned as to the occurrence of any of the study endpoints over the previous 6 months. Notification of a potential study endpoint will trigger the collection of information for endpoint confirmation and adjudication by the EAC. All such events will be recorded in source documents and in the appropriate section of the Case Report Form (CRF). Confirmation of endpoints will be ascertained by collecting and forwarding information from the following sources within 90 days of learning the event:

- Details from medical records from the usual treating physician or practice–held medical record.

- Medical records obtained by letter/fax/email contact with other treating specialist physicians or secondary/tertiary medical care centers.
- CRFs containing standardized questionnaires for the assessment of cognitive function, physical function and quality of life.
- In Australia, hospital records/discharge summaries, pathology reports, Medicare and Pharmaceutical Benefits Scheme (PBS) records.
- Death Certification: In both Australia and the U.S., the respective National Death Index will be routinely sourced for notification of death not identified through the above processes.
- For dementia endpoint ascertainment, the Dementia Adjudication Panel will be responsible for providing the assessment according to DSM-IV criteria. An individual's 3MS score below 78, or fall over time by more than 10 points adjusted for age and level of education, will trigger additional and uniformly applied cognitive and functional testing by research staff and the collection of ancillary data (laboratory tests and a CT or MRI) for endpoint ascertainment.
- For significant disability as a primary endpoint, the loss of one of the Katz Activities of Daily Living will be defined as self or proxy report of having 'a lot of difficulty with' or 'unable to perform' one or more ADLs at the annual follow-up contact. For all deaths where disability data is missing in the year preceding death, we will conduct a proxy interview to determine whether there was disability in the year prior to death and its approximate onset. We will also conduct a follow-up phone call to determine the persistence of the reported disability by repeating the same interview items.

5.5 Endpoint Adjudication Process

Each study endpoint will be sent to the ASPREE Data Management Center (DMC) using the specific *Endpoint Notification* CRF. In addition, all supporting information derived from medical records, hospital discharge summaries and medical investigation reports will be collected, copied and sent to the ASPREE DMC. Original data will be retained at each study site as source data. Participants maintain the right to withdraw from ASPREE and also the right to decline any further contact or data collection. The possible options for phone, medical records or health registries data collection are outlined within the ASPREE Consent Form.

On receipt at the ASPREE DMC, all endpoint information will be collated. An ASPREE Data Center Manager will be responsible for the presentation of study endpoint documentation to the EAC on a pre-determined schedule. Additional information requested by the EAC will be

communicated to the site via the Data Center Manager. The EAC will meet via video or teleconference on a quarterly basis to discuss: a) the quality of endpoint information being received, b) the adjudication process and any difficulties or operational concerns, and c) the rate of endpoint accumulation. EAC will consist of individuals from the U.S. and Australia chosen by the Steering Committee. Specialist sub-committees will be appointed, such as the Dementia Adjudication Panel, to adjudicate on specific endpoints with a nominated person as member of the EAC. Case material will be reviewed by at least two members and if there is disagreement, a third member will be asked to adjudicate, or in the case of the Dementia Adjudication Panel, the whole committee will decide.

6 STUDY POPULATION

6.1 Inclusion criteria

- African American and Hispanic men and women 65 years of age and over (in the U.S.), all other men and women 70 years of age and over
- Willing and able to provide informed consent, and willing to accept the study requirements

6.2 Exclusion criteria

- A history of a diagnosed cardiovascular event defined as MI, congestive heart failure, angina pectoris (± nitrate use), stroke, transient ischemic attack, >50% carotid stenosis or previous carotid endarterectomy or stenting, coronary artery angioplasty or stenting, coronary artery bypass grafting, or abdominal aortic aneurysm.
- A clinical diagnosis of atrial fibrillation.
- A serious intercurrent illness likely to cause death within the next 5 years, such as terminal cancer or obstructive airways disease.
- A current or recurrent condition with a high risk of major bleeding, e.g. cerebral aneurysm or cerebral AV malformation, any bleeding diathesis, gastrointestinal malignancy, recent peptic ulcer, liver disease, esophageal varicosities, uremia, aortic aneurysm or any other condition known to be associated with a high risk of serious bleeding.
- Anemia, i.e. hemoglobin level below the normal value for the gender of the participant (males: <12 g/dL, females: <11 g/dL)

(Note: Hemoglobin levels within the normal range in a participant taking therapy for anemia will not be an exclusion criterion).

- Absolute contraindication or allergy to aspirin.
- Current participation in a clinical trial.
- Current continuous use of aspirin for secondary prevention.
- Current continuous use of other anti-platelet drug or anticoagulant.
- A systolic blood pressure ≥180 mmHg and / or a diastolic blood pressure ≥105 mmHg
- A history of dementia or a Modified Mini-Mental State Examination (3MS) score ≤ 77 as measured at Visit 1: *Lifestyle Profile and Screening*.
- Severe difficulty or an inability to perform any one of the 6 Katz ADLs, as determined at Visit 1: *Lifestyle Profile and Screening*.
- Pill-taking compliance below 80% during the placebo run-in phase.

People with previous or current use of aspirin for reasons other than secondary prevention may enter the trial, provided they agree to cease existing use of aspirin and understand that they may be subsequently randomly allocated to low dose aspirin or placebo. In the case where a person has been advised by their doctor to take aspirin, the person should seek advice from their doctor before participating in the study.

6.3 Participant Recruitment

Community-based recruitment for the ASPREE study will follow one of the following two strategies depending on the preferences and experience of the investigators, the characteristics of the population to be recruited, and the particular attributes of the recruitment site:

(i) family / general practice or other medical practice - based recruitment;
(ii) community-based recruitment.

6.3.1 General Practice recruitment (in Australia)

The resource established for the ANBP2 collaboration for the conduct of large-scale trials in family practice [83-86] will be used. That study involved the recruitment and follow-up of over 6000 participants in a family practice setting. The recruiting base involves collaborating Departments of General Practice in universities across Australia which help to identify and recruit regional medical co-ordinators and research staff. The clinical research staff will then approach individual general practitioners (GPs) to support the project and to explain the rationale and implications

for the physician's practice. GPs indicating a willingness to receive more information will be sent an information package further describing the study and a site visit will be made to discuss the study with the regional medical co-ordinator or a senior researcher. For group practices, each physician will be approached individually by an initial telephone call, but the site visit will be arranged with all interested physicians in the practice. A study site evaluation checklist will be completed to ensure that the study site has the required facilities and resources to participate satisfactorily in the ASPREE study. Following the site visit and confirmation of participation, the *GP Investigator Registration* form(s) and *Practice Registration* form are completed, and the clinic becomes a registered ASPREE clinical practice.

Clinic databases from each registered practice will be reviewed for participant inclusion and exclusion criteria by authorized clinical research staff. These staff members also sign confidentiality agreements to become agents of the ASPREE clinical practice in which they are working. Following the generation of a list of potential study participants, the GP then reviews the results of the search to ensure that invitees meet inclusion and exclusion criteria. Each individual who appears to meet these criteria is sent a letter from their usual GP inviting them to consider participation in the trial. The letter advises those who are interested to call a toll free 1800 number to discuss his/her participation in the study. At this initial telephone contact, willingness of the person to participate will be ascertained and information relating to exclusion criteria will be sought and recorded on the Phone Screening form and an information package will be sent to the participant by post. For those willing to participate and with no obvious exclusion criterion, an appointment will be made to attend the practice premises for the screening visit and subsequent enrolment by the GP who will re-confirm their eligibility for the study.

Minority recruitment: In Australia, 70% of indigenous persons in non-remote areas use general physician's services (where they are encouraged to self-identify). We are therefore likely to be able to identify such persons through our usual recruitment strategy.

6.3.2 Community-based recruitment (in the U.S.)

Recruitment for ASPREE in the U.S. will use two general approaches, community-wide and within participating clinic sites. We have found that recruitment is most effective with several different approaches operating in parallel. This allows for a rapid shift in emphasis on activities from one method to another. It also ensures a steady flow of participants thereby avoiding nonproductive and expensive 'down time'. Careful and frequent tracking of recruitment to randomization rates is essential. Well-established research collaboration networks exist within

each of the U.S. ASPREE regional hubs that have consistently met recruitment timelines. We recognize both the necessity and challenges of recruiting a large sample of seniors of both genders and substantial ethnic diversity. Past methods, which have repeatedly achieved or exceeded enrollment goals, include 1) mass brochure mailing, 2) use of media, and 3) referrals. Each of these methods is described below.

Minority recruitment: In the U.S, approximately 70% of the study population will be minorities. We will accomplish this by involving proven minority recruitment sites and by creating recruitment cells that will fill as participants are randomized into the trial. We have designed our strategy of site selection and minority recruitment resources to achieve 53% Hispanic and 47% Black with other minority groups represented in smaller numbers.

We have a multifaceted recruitment plan that is based on the successful prior experience of the U.S. Coordinating Center, the Hubs and local clinical centers. Our approaches will include mailings, mass media (radio, print, internet) and referrals. These methods are designed to take advantage of the uniqueness of each metropolitan area and the various strengths and past experiences of all U.S. ASPREE participating units in order to achieve a demanding minority recruitment goal. We will use multiple methods simultaneously, and will provide guidance and training as needed from the broad resources of all U.S. sites.

6.4 Participant discontinuation

As the aim of the study is to assess ultimate outcome following randomization to one of the treatment arms, attending physicians will be strongly encouraged to continue participants on their randomized treatment. However it is recognized that situations will arise that will necessitate withdrawal of a participant from the study. Such situations include a participant's desire to withdraw, or attending physician's decision to withdraw the participant. If a participant withdraws from the study altogether (i.e. from taking study medication and from attending annual visits) or is removed from the study for any reason, this will be recorded on the *Annual Visit* CRF.

When the endpoint is non-fatal, participants will be requested to continue to attend routine annual assessments for the ascertainment of further endpoints. Participants who are unwilling to continue participation will be asked for permission to allow the investigators to continue to contact them to ascertain the occurrence of clinical endpoints and to collect medical information via their medical records.

6.5 Contingency plans for participant well-being

Participants will be advised to seek advice from their usual treating physician for any medical condition arising during the course of the study. Treating physicians will be provided with information about the study to maintain the participant's involvement in the study (wherever possible).

The International Operations Committee will provide generic advice about the management of issues that may arise during the course of the study but it will be made clear to family physicians in Australia that these suggestions may be over-ridden by particular clinical circumstances. For example, in the case of iron-deficiency anemia identified during the study, family physicians will be advised to suspend trial medication while they exclude malignancy, dietary and other causes. (Hemoglobin results will be provided to the family physician on an annual basis as part of the routine follow-up of each patient.) In the U.S., the study medication will be discontinued and the clinical center physician will refer the participant to their usual source of medical care to evaluate other causes of anemia. Recommencement of aspirin therapy may be initiated if a remediable cause is determined but this will be at the discretion of the participant and their usual medical care provider. Other contingencies will be addressed with physicians at regular intervals through a study newsletter.

6.5.1 Follow up of participants 'off protocol'

"*Off protocol*" is defined as: study participants who commence aspirin therapy or cease trial medications on clinical grounds or because of a lack of preparedness to continue in the study.

Regardless of the decision to continue with the study medication, the participant will be asked to attend all scheduled follow-up visits as if they were maintaining full participation. Those who are unwilling to do this will be asked if they will agree to phone call/mail follow-up and, in addition, for approval to continue follow-up through surveillance of their clinical records (or just the latter, if they are unwilling to be contacted in any form). Those who refuse all forms of follow-up will have their outcomes assessed in the national morbidity and mortality databases. All participants will be asked to provide approval for access to this information as part of the study enrollment procedures. In all cases where a participant ceases taking their study medication, the reason for discontinuation, the date of discontinuation and date of the last dose of study medication, should be recorded.

6.5.2 Anemia diagnosed in a study participant

Hemoglobin results will be provided to the usual treating physician on an annual basis as part of

the annual follow-up report for the participant. The treating physician will be advised to follow their routine clinical practice for the management of patients with anemia. When the cause of the anemia is determined to be blood loss and when the cause of blood loss is identified (e.g. a bleeding colonic polyp), encouragement will be given to recommence the trial drug once the underlying cause is treated. Similarly, physicians may choose to advise participants to recommence their study medication if the anemia has responded to therapy such as iron replacement.

6.5.3 Major surgery

Participants will be advised to contact the research staff or research clinic when any surgery is planned. Their physician will be advised to treat the participant as if they are taking aspirin and that the participant may temporarily cease the trial drug if they consider it advisable. No un-blinding will occur. Participants will be encouraged to recommence study medication at a time determined by the participant's surgeon in consultation with their usual treating physician.

6.5.4 Major bleed

Participants will be advised to cease randomized study medication on diagnosis of a non-traumatic major hemorrhage, e.g. a bleeding peptic ulcer. The participant will be followed for the duration of the study. Such a trial medication cessation may be temporary or permanent depending on the condition and the clinical judgment of the physician or surgeon.

Gastrointestinal hemorrhages or hemorrhages at other sites that required transfusion, hospitalization, prolonged hospitalization, surgery or are fatal will be considered as clinically significant bleeding. Source information from clinical case notes and hospital medical records related to these events will be collected and sent to the ASPREE Data Management Center. A clinically significant bleed may lead to temporary or permanent trial medication cessation, according to the treating physician or surgeon's clinical judgment, but not exit from the trial. There is no standard accepted clinical trial definition of major gastrointestinal bleed in Australia. Our operating definition has been accepted by the Australian Gut Foundation.

6.5.5 Abdominal discomfort/dyspepsia

Participants will be advised to discuss abdominal discomfort / dyspepsia with their usual treating physician. Both physician and participant will be advised that the ASPREE pilot studies suggest that only 20% of such symptoms are likely to be due to the active trial drug. If symptoms are persistent the recommendation will be to cease the study medication for a trial period and then re-challenge. If symptoms recur then the physician may add other medication (e.g. a proton

pump inhibitor) or cease the trial drug. The participant will be asked to continue in the trial.

6.5.6 Commencement of regular non-steroidal medication (NSAID)

Participants will be advised to avoid continuous NSAID use. Additional advice to physicians and participants is that treatment with NSAIDs (including aspirin) should be at the lowest dose and for the shortest period possible for clinical effectiveness. Details of all NSAID use and duration will be recorded.

6.5.7 Development of an indication for aspirin

Participants who develop a recognized clinical indication for continuous use of aspirin may commence routine aspirin and will continue in the study for observation of endpoints.

6.5.8 New diagnosis of diabetes

All participants will be advised to discuss a new diagnosis of diabetes with their usual treating physician. The decision to cease the study medication and commence active aspirin will be made by the physician in consultation with their patient. The participant will be asked to continue with routine follow-up visits.

6.5.9 New diagnosis of atrial fibrillation

To aid in the detection of undiagnosed chronic atrial fibrillation (AF), during the annual visit research staff will check the participant for an irregular pulse, either by palpation or by failure of the automated blood pressure instrument to collect a reading. If the annual visit takes place in a clinic, most clinics will then run an ECG to determine if the irregular rhythm is non-pathological (e.g. due to multiple ectopics). If AF is detected (confirmed by the clinic physician), the participant will be referred to their usual primary care clinic or doctor and will cease study medication. In the event that visits do not take place in the clinical setting (mostly in Australia), or there is no access to an ECG machine, the participant will be referred directly to the primary care doctor or general practitioner for diagnosis and further investigation if required.

6.5.10 Commencement of warfarin or other antiplatelet drugs

A prescription of warfarin, aspirin or other anti-thrombotic drug will be reason for stopping study medication but the ASPREE participant will continue to be followed for the duration of the study. If warfarin or other anti-thrombotic drug is prescribed for a limited period (such as for DVT), study medication will be ceased for that period, but may be resumed after anti-thrombotic therapy has been stopped.

6.5.11 Commencement of aspirin on own volition

Participants entering the study will be provided with information about the need to avoid chronic self medicated aspirin therapy. At each annual visit, the participant will be asked whether there has been a change in their chronic drug use, including aspirin use, and, if so, will be asked to discuss this with their doctor and encouraged to avoid chronic aspirin use.

The participant will be reminded of this requirement at annual follow-up visits and through the trial newsletter and other communications. Although it will be discouraged, participants are entitled to commence aspirin if they so choose. Regardless, all randomized subjects, whether continuing with randomized treatment or not, will be followed in an identical fashion for the duration of the trial.

In Australia, the participant's usual treating physician is a co-investigator and is unlikely to recommend low dose aspirin without a recognized clinical indication. Participants will also be given cards to carry in their wallets to show other health care providers, and are educated on the forms of aspirin that are on the market.

7 MEASUREMENTS, CRFs AND ANALYTICAL METHODS

7.1 Schedule of study visits

The trial is primarily designed to assess mortality and morbidity although other measures will be determined. The ASPREE Measurement and Study Activity schedule for each of the study visits is summarized in Table 4.

7.1.1 Lifestyle Profile and Screening visit (Visit 1)

Measurements and data will be collected by the research staff after the participant has provided informed consent to participate in the study. Each study participant will be assigned a unique identification number, which will be used throughout the study. The information to be collected at this visit (and thereafter at some or all Annual Visits* - see Table 4) includes:

a) Basic demographic and lifestyle factors including living situation*, ethnicity and language, personal medical history*, smoking history*, alcohol use*, medication history* and level of education.

b) Family history of acute MI, stroke, renal disease, colorectal and other cancers, or dementia – identified by the participant and from medical case notes.

c) Blood pressure and heart rate*: measured in the seated position following 5 minutes of rest using an oscillometric device. The average of three measurements taken 1 minute apart will be recorded.

d) Cognitive function* and depression: assessed using the Modified Mini-Mental State Examination (3MS) score [76] and the Center for Epidemiologic Studies – Depression 10 (CES-D) [77] questionnaires. The 3MS will be implemented as part of the screening of participants (where the score will be used to determine eligibility for the study) and will be administered again at years 1, 3,5 and 7. The CES-D is a self-administered questionnaire used to screen for depression. It will be used in association with the 3MS, as depression is a confounder for cognitive function ascertainment, and also to ascertain new onset depression.

e) Physical disability*: assessed by the participant's self-reported ability to perform the 6 Katz Activities of Daily Living (ADLs), which form a component of the Lifestyle Interventions and Independence for Elders (LIFE) Disability questionnaire.[81]

f) Referral to a local Pathology Provider (in Australia) or direct collection of a fasting blood sample* (~12 ml, for the measurement of total cholesterol, LDL-C, HDL-C, triglycerides, glucose, creatinine and hemoglobin) and urine spot test (minimum of 20 ml, to measure the albumin : creatinine ratio and detect the presence of microalbuminurea). Where available, a point-of-care instrument will allow prior screening for low hemoglobin levels.

g) Placebo medication for the run-in phase of 4 weeks will be dispensed to enable subsequent determination of medication compliance during this period.

h) Review of inclusion / exclusion criteria.

**Measurement/ Activity**	**Lifestyle Profile & Screening (Visit 1)**	**Assess-ments & Eligibility (Visit 2)**	**Follow -up (1yr)**	**Follow -up (2yr)**	**Follow -up (3yr) ****	**Follow -up (4yr) ****	**Follow-up (5yr)****	**Follow-up (6yr)****	**Follow -up (7yr)****
**Review inclusion/ exclusion criteria**	X	X							
**Obtain informed consent**	X								
**Dispense study medication**	X (Run-In only)	X	X	X	X	X	X	X	X
**Assess medication compliance**		X	X	X	X	X	X	X	X

**Concomitant medications**		X	X	X	X	X	X	X	X
**Blood pressure & heart rate [a], height [b], weight [c] & abdominal circumference [c]**	X [a]	X [bc]	X [ac]	X [ac]	X [ac]	X [ac]	X [ac]	X [ac]	X [abc]
**Demographics, family & personal history & lifestyle factors**	X		X	X	X	X	X	X	X
**Laboratory testing:**<br>**-** Fasting blood: total cholesterol, HDL, LDL, triglyceride, glucose, creatinine & hemoglobin<br>**-** Urine: albumin:creatinine ratio and microalbuminurea	X		X	X	X	X	X	X	X
**Quality of Life**<br>**-** SF-12		X	X	X	X	X	X	X	X
**Assess cognitive function & depression**<br>- 3MS [a] , CES-D [b] SDMT [c] HVLT-R [c] & COWAT [c]	X [ab]	X [c]	X	X[b]	X	X[b]	X	X[b]	X
**Assess physical disability**<br>- KATZ ADLs	X		X	X	X	X	X	X	X
**Assess physical function**<br>- Walk test and grip strength		X		X		X		X	X
**Clinical event reporting**<br>- Questionnaire & medical record review		X	X	X	X	X	X	X	X
**Retinal Imaging^**		X			X				

Table 4. ASPREE Measurement and Study Activity Schedule
(**X** indicates that all measures are carried out except where superscripts [a,b,c] indicate those tests that are the only ones performed at the designated time point.) ** Final annual visit will take place in years 3, 4, 5, 6 or 7 depending on year of randomization. Final visit measurements will be the same as those indicated for year 7.

Telephone calls will also take place in between annual visits for purposes of retention and for clinical event reporting. A person-to-person call will occur in the U.S. 4 weeks after Visit 2, automated or person-to-person calls will occur at 3 and 9 months and direct person-to-person calls will occur at 6 months between annual face-to-face visits. At the 6 month call, participants will be asked specifically about all endpoints and will be administered the Katz ADL's.

^ Retinal Imaging will only be conducted on participants in Melbourne or other locations in Australia subject to the availability of imaging equipment.

7.1.2 Post screening visit study activity (Run-in phase between Visits 1 & 2)

Following the screening visit, blood samples will be collected by the local Pathology laboratory provider (in Australia). Laboratory pathology results will be available to the study site within the first two weeks in the run-in phase of the study. These results will be used to satisfy inclusion and exclusion criteria. In Australia, the participant will also consult his / her family physician during this run-in phase, whereupon the family physician will complete the GP

Inclusion/Exclusion Criteria Worksheet.

7.1.3 Determinations at Eligibility and Assessments visit (Visit 2)

At the end of the 4 week run-in phase, the ‘Assessments & Eligibility visit’ (Visit 2) will take place. At this visit (and, unless stated otherwise, at every Annual Visit) the following measurements and study activities will be conducted:

a) Medical morbidity – medical record review and questionnaire / participant self-reporting.

b) Short Form (SF) -12 score [82] – self-administered questionnaire for quality of life.

c) Additional cognitive function tests: the Symbol-Digit Modalities Test (SDMT) [77], the Hopkins Verbal Learning Test (HVLT-R) [79] and the Controlled Oral Word Association Test (COWAT) [80]. These tests will then be performed in years 1, 3,5 and 7 (close-out), in conjunction with the 3MS and CES-D, for confounding of depression and new onset depression – see Table 4.

d) Height – measured, without shoes standing against a wall using a calibrated stadiometer. Measured again at Year 7 (or whenever the final annual visit occurs).

e) Weight and waist circumference – measured following the removal of excess clothing and with calibrated scales. Waist circumference will be measured according to the NHANES III Protocol (http://www.ncbi.nlm.nih.gov/books/bv.fcgi?rid=obesity.box.236).

f) Physical function testing – timed gait speed test for 3m (8ft) [87] and hand grip strength measured on a grip strength dynamometer. [88] Physical function testing will be performed in years 2, 4, 6 and 7 (close-out) – see Table 4.

g) Concomitant medications, indication and year commenced – participant self-reporting, medical record review and family physician report.

h) Medication compliance – the number of pills returned at this visit to the number expected with 100% compliance. Adequate adherence to placebo during the run-in period is an inclusion criterion for the study and is defined as pill count greater than 80% of those required to be taken during this phase. If considered necessary, the placebo run-in test for compliance may be repeated.

i) Information on relevant clinical events.

j) Retinal imaging – this will only be conducted on participants in Melbourne or other locations in Australia subject to the availability of imaging equipment. Consenting participants will have standardized non-mydriatic retinal photography. After 5 minutes of dark adaptation, colour retinal photographs will be taken without pharmacological pupil dilation using Canon NMR 45 digital fundus cameras. Two retinal photographs, each

centered on the optic disc (ETDRS standard field 1) and macula (standards field 2) respectively, will be taken from both eyes. Images will be collected and archived in an image library. No analysis of the images will be carried out as part of ASPREE.

Participants' contact details will also be confirmed. In Australia, this will ensure the dispensing of study medication to the correct mailing address.

7.1.4 Randomization Procedure

Following the completion of data collection at Visit 2, the Randomization form will then be completed. If inclusion criteria are satisfied, participants will be randomized into the ASPREE study via the internet. Password protected access is required for study personnel. Staff will be required to enter their unique ASPREE Identification number along with the following key information: study site number, ASPREE participant identification number, age and gender of participant, and confirmation of inclusion / exclusion criteria. Sites will also be asked to do a repeat entry of the participant's acrostic to ensure the right person is being randomized.

Computer-generated medication numbers will be provided to trial sites through the web portal. The randomization list will be generated by an independent statistician. This arrangement will ensure that the randomization code remains inaccessible to all study staff and senior investigators. The randomization list will be generated using the STATA 'ralloc' procedure with randomization stratified for site and age (<80 yrs and ≥80 yrs). Following the completion of the randomization process by the research assistant / nurse, a study medication number will be provided. All staff remain blinded to treatment allocation through the randomization procedure. The subsequent distribution of drug is detailed in Section 8. Records of all participants who have undergone screening and randomization will be made by completing the appropriate tracking log.

7.1.5 Annual Visits

At Annual Visits, measurements undertaken at baseline (refer to sections 7.1.1 and 7.1.3) will be repeated using identical protocols, in accordance with the ASPREE Measurements and Study Activity Schedule (Table 4). Additionally, Serious Adverse Events (SAEs) and potential clinical endpoints will be collected by participant report and medical record audit (in Australia). Following notification, clinical information relating to the SAE or endpoint will be sourced through: a) medical record review and b) hospital records and discharge summaries. Specific procedures for endpoint ascertainment and adjudication have been outlined previously in sections 5.4 and 5.5, respectively, of this protocol. In order to detect new onset atrial fibrillation

during the study, research staff will measure the pulse rate at annual visits, as described above in section 6.5.9.

7.1.6 Participant Retention Plan

Based on the experience of investigators in Australia (e.g. ANBP2 [83-86]) and in the U.S. (e.g. ALLHAT [89-93]) high rates of participant retention will be achieved using multiple strategies including:

- Compliance and retention will be encouraged by 3, 6 and 9 month research staff direct phone contact interspersed with annual face-to-face visits.
- Contacts details (including alternate contacts) will be updated during face-to-face meetings. Additional contacts are used in the situation where a participant changes address without contacting the study center.
- Newsletters will be sent to family physician co-investigators and participants on a regular basis (~2-4 per year) detailing study progress.
- Anniversary Cards may be distributed to subjects on an annual basis as a reminder of their participation.
- In Australia, the study staff member and family physician co-investigator remain in contact.

If a participant is 'lost to follow-up' despite measures detailed above, the following steps will be undertaken:

- Tracking through third party contacts (details of these are updated annually). Details for up to 3 additional contacts, who do not reside at the same address as the participant, will be obtained at randomization.
- Crosschecking with morbidity and mortality registries.
- In the U.S., data linkage through Medicare numbers will provide updated medical information and clinical events in both countries.
- In Australia, the research staff member will be able to also audit practice and hospital records in the event a participant is unable to be contacted.

7.1.7 Telephone Contact

Each participant will be contacted by telephone, initially if receipt of trial drug has not been registered (in Australia) or by 4 weeks after study medication is provided (in U.S.). Three, six and nine monthly phone calls after baseline and after each annual visit will be made to encourage compliance to treatment allocation. In addition, at the 6 month phone contact, site staff will also gather information about new onset of serious adverse events or potential study

endpoints. Data to be collected during the telephone calls include:

- Compliance – the research assistant / nurse will discuss the participant's adherence to medication since the previous clinic visit.
- (6 month call) Serious adverse event and endpoint notification will be achieved by participant report (clinical information relating to these events will be sourced as for Annual Visits).
- (6 month call) Katz ADLs and Living Situation.

7.1.8 Other Actions

If the participant does not proceed to randomization, then the reason for non-randomization is to be documented and collected on the designated CRF.

Any test measurement result that is outside the normal range, at baseline or annual visits, will trigger a notification to the participant's GP in Australia and will be provided to the participant's regular treating physician in the U.S. Such measurements include: 3MS, CES-D, hemoglobin and glucose levels, blood lipids and triglyceride, urine albumin : creatinine ratio, and blood pressure levels (for details, see Appendix 1: Endpoints and Other Measures).

7.2 Case Report Forms (CRFs)

CRFs are used to record clinical study data and are an integral part of the study and subsequent reports. The CRFs, therefore, must be legible and complete. All forms must be filled in using a black ballpoint pen and errors must be crossed out but not obliterated and the corrections must be written above or beside the error on a free space. All corrections must be initialed and dated.

CRFs will be printable by the sites via the ASPREE website for each participating family practice (in Australia) or study site. At the end of a participant visit, the research staff will transfer the data directly onto electronic CRFs that are accessible via the ASPREE website, and retain the original hard-copy CRFs and other source documents, stored in the participant's file.

CRFs must be kept current to reflect the participant's course throughout the study. Participants are not to be identified on the CRF by name. Appropriately coded identification (GP, Practice and Subject Identification Numbers) and an acrostic of the participant's initials must be used.

7.3 Data Analysis

The trial will be analyzed by statisticians based at the ASPREE Data Management Center,

Department of Epidemiology and Preventive Medicine, Monash University, and the Berman Center for Outcomes Research, University of Minnesota.

7.3.1 Interim analysis

Rather than stipulate interim analysis we leave consideration of this to the independent NIH-appointed DSMB. A proposed interim analysis is described in the Data and Safety Monitoring Plan of the DSMB.

7.3.2 Final analysis

All primary and secondary outcomes will be in the form of time-to-event data and rate ratios will be calculated using univariate Cox proportional hazards regression to directly compare event rates between treatment groups. In analyses of secondary endpoints, death due to causes other than those specified by the endpoint and loss to follow-up (see below) will be considered as censoring events. Given the large sample size, we anticipate randomization will adequately balance baseline characteristics of participants in the two treatment groups. If necessary, a secondary set of analyses will be performed to adjust for baseline characteristics that are found to be imbalanced between groups to the extent of a 0.25 standard deviation difference in means (quantitative measures) or an odds ratio of 1.5 (binary measures). These analyses will be conducted using multivariate Cox proportional hazards regression models.

The primary and secondary endpoints will be analyzed according to intention-to-treat principles, i.e. according to the group to which participants were randomized and without reference to their actual compliance with assigned treatment. No statistical adjustment will be made for the multiple secondary endpoints in their analysis [94] [95] but the reporting of all secondary endpoint analyses will make clear whether the primary endpoint was statistically significant [95] and will state the number of secondary endpoints proposed *a priori* in the study protocol [94].

In the survival analyses, loss to follow-up will be considered a censoring event. This equates to an assumption that data is missing at random given the participant's treatment group and the timing of their loss to follow-up. The adequacy of this assumption will be checked in sensitivity analyses that will include both a multiple imputation by chained equations (MICE) approach and adjustment for baseline covariates predictive of propensity for dropout. [96]

7.3.3 Further details for statistical analyses

Baseline characteristics that are quantitative measurements will be checked across the range of measures as part of data cleaning prior to statistical analysis. This will ensure that perceived outliers are not data entry errors. A genuine outlier will be defined as a measurement that is

greater than three standard deviations from the mean; on a log-transformed scale if measurements are approximately log-normally distributed on their original scale. Regression diagnostics will be used to assess the influence of genuine outliers on treatment comparisons in analyses that include a quantitative baseline characteristic. Where outliers are found to exert such an influence, a secondary analysis will be performed with the outliers removed and the results of the two analyses will be reported.

7.3.4 Pre-specified sub-group analyses

Pre-specified analyses will be undertaken within sub-groups where there may be a difference in the net benefit of aspirin from the remainder of the population. These sub-group analyses will utilize appropriate interaction terms in Cox proportional hazards regression models. The p-values for these interaction terms will be used to test for heterogeneity of treatment effect of aspirin between sub-groups. These sub-groups include:

a) **Males *versus* females:** Recent data from the Women's Health Study (WHS) [16] showed a significant reduction in vascular events in females aged 65 years and over. A meta-analysis [19] suggested that amongst younger females the principal effect of low-dose aspirin is to reduce the risk of stroke with little effect on MI except in those aged 65 years and above, whereas in males the effect is a reduction of MI with minimal effect on stroke.

b) **Age below and above study median** (expected to be approximately 75 years): The balance of risks and benefits due to aspirin may differ between age groups as a result of different rates of mortality, cardiovascular risk, cognitive decline, other disability and risk of adverse effects (as defined for our secondary objectives).

c) **Country:** U.S. *versus* Australia.

d) **Ethnicity:** Whites *versus* African Americans *versus* Hispanics *versus* other (where 'other' will include Native Americans, Asians and Aboriginal Australians).

e) **Diabetes:** The presence or absence of a diagnosis of diabetes at baseline.

f) **Hypertension:** Hypertensives *versus* non-hypertensives. Hypertension is defined as those who are on treatment for high blood pressure or those with blood pressure recorded above 140/90 mmHg at study entry.

g) **Smoking:** Current *versus* Never or Former smokers.

7.4 Dissemination and implementation of results

7.4.1 Objectives

Implementation of a theoretically based dissemination intervention, similar to that of the Antihypertensive and Lipid-Lowering Heart Attack Trial (ALLHAT).

a) To increase awareness of the ASPREE trial by disseminating results via repetitive messages through scientific and professional channels.

b) To provide physicians, physician's assistants, practice nurses and nurse practitioners with cues to action via distribution of office posters, prescription cards, and other educational materials.

7.4.2 Approach and methods

The following represents various strategies to disseminate study findings and recommendations.

- *Web site.* The ASPREE web site will provide information to the professional and scientific community as well as to the public regarding study results and recommendations (in addition to providing a means by which authorized ASPREE personnel can send data, using electronic CRFs). The web site will consist of published journal articles, newsletters, presentations, a frequently asked questions (FAQs) section, links to other appropriate web sites, and downloadable information for PDAs, iPhones and other devices.
- *Publication.* Study findings and recommendations will be published in appropriate scientific journals to be made available to the scientific community.
- *Slide presentations.* PowerPoint presentations will be constructed to provide study rationale design, results, and implications. These will be available for formal presentations, office or departmental seminars, grand rounds, or local medical society meetings. Select PowerPoint files, i.e. major outcomes, will be available in other languages, including Spanish. Files will be accessible via the ASPREE web site. A set of PowerPoint presentations will also be developed for consumers.
- *Formulary systems approach.* Similar to the ALLHAT dissemination, pre-identified formulary systems will receive a summary of study findings, recommendations, relevant articles, cost-effectiveness information, and strategies for implementation to improve care.
- *Clinical guidelines.* Recommendations in guidelines will assist the facilitation of the dissemination intervention. For example, the ASPREE study results may be reinforced by

the existing US Preventive Task Force and American Heart Association guidelines for the evaluation, prevention and management of cardiovascular disease in adults.

- *Education Materials.* Development of posters, reference cards, and consumer brochures will be effective tools for disseminating study findings and recommendations. Office posters and reference cards will assist sites in management. Consumer brochures will educate the public and provide the necessary information to encourage consumers to speak to their usual health care providers regarding their management.

8 STUDY MEDICATION AND SUPPLIES

8.1 Study treatments

Participants in the study will be allocated to one of two treatments: a) Acetylsalicylic acid (ASA) 100 mg: enteric-coated un-scored white tablet or b) placebo: enteric-coated un-scored white tablet with identical appearance. Study medications are provided by Bayer Pharma AG. A 100mg dosage was selected as this is the common international dose. The enteric coating will ensure that both active and placebo medication have an identical taste.

Run-in placebo – A box of placebo medication for 4 weeks will be given to participants at the Lifestyle Profile and Screening visit (Visit 1). If for whatever reason a participant cannot attend their Assessments and Eligibility visit (Visit 2) at 4 weeks, he/she will receive another supply of placebo if it is required for compliance assessment.

Assessment and Eligibility visit (Visit 2) – Subsequent to this visit, each participant will be provided with a 12 month’s supply of study medication: either aspirin or placebo.

Annual Visits – At each Annual Visit each participant will be provided with the next 12 month supply of their allocated study medication. If a participant is unable to attend an annual visit within a month of the scheduled date, and will run out of study medication, the research staff can mail out the medication to the participant provided a new appointment has been scheduled.

8.2 Drug packaging

A bulk supply of enteric-coated active and placebo tablets has been provided free of charge by Bayer Pharma AG. The medication will be packaged and labeled according to the randomization code. Participants will be provided with a 12 month supply on each occasion. Packaging will feature the name of the study (ASPREE clinical trial), sponsor, supplier, batch number, expiry date, contents, the fact that the content is pharmacy medicine for use in clinical trials only, instructions for use, and a warning to keep out of reach of children. Each medication

container will be labeled with a pre-printed, unique medication Identification (ID) Number. Once dispensed, this number will also be recorded in the participant's file in the *Study Drug Accountability* Log.

8.3 Administration of study medication

One tablet is to be taken daily with advice to take at the same time each day, allowing 24 hr between administration.

8.4 Dispensing and randomization

Medication for the run-in phase will be dispensed to the participant by the study staff at Visit 1. Each participant enrolled in the study will be dispensed a box or bottle containing 40 placebo tablets. The Practice ID number, Subject ID number and participant's acrostic will be written on the container of run-in medication. When the participant returns for the Assessments and Eligibility visit (Visit 2), medication compliance will be checked prior to randomization.

Following the completion of the randomization process (Section 7.1.4), in the U.S. a 12 month supply of study medication will be provided in person to the participant at the conclusion of the visit. In Australia, study medication will be posted to the participant. The participant will be asked to confirm receipt of the drug by calling the IVRS automated telephone system. Where confirmation of receipt has not been made within 10 days of dispatch of the drug, research staff will contact the participant to ensure receipt. Attempts to contact the participant will continue until receipt has been confirmed. In the rare instance of missing or undelivered drug, the pharmacy will re-issue medication which will be sent for home delivery to the participant.

Subsequent allocation of study medication will be provided in person by research staff at each Annual Visit, in both the U.S. and Australia. The Medication ID number assigned to the participant will remain the same for the duration of the study. The participant will therefore receive study medication with the same Medication ID number at each visit.

One *Study Drug Accountability* Log will be provided for each study participant. These Drug Accountability Logs record the drug dispensed to the participant and drug returned from the participant.

The *Study Drug Accountability* log will record, at minimum, the following information:

- Practice ID number and name
- Subject ID Number and participant acrostic
- Visit Number

- Medication ID number
- Date dispensed (initials of dispenser)
- Date of medication return (initialed by research staff)
- Number of tablets returned
- Date of medication destruction
- Comments section to allow for explanation of discrepancy, if applicable

8.5 Drug storage

Upon receipt of the study medication from Bayer Pharma AG, it will be stored at room temperature (15-30°C).

All placebo medication for the run-in phase, as well as subsequent years' study medication supply, will be distributed to ASPREE sites and stored in a secured area with restricted access, at room temperature under the supervision of the site Principal Investigator.

The receipt, storage, dispensing, accountability and study medication collection, for both the run-in and randomization phases, are the responsibility of each site's Principal Investigator.

8.6 Drug accountability and dispensing logs

All medication containers, whether empty or containing unused tablets, must be collected to allow medication compliance to be determined (via a pill count) and to allow for proper destruction of the medication. Unused tablets are to be returned at the Annual Visit for compliance checking and a new supply of study medication will be issued. Lost medication packs will be replaced and sent to the participant by mail, with all details recorded to allow an audit trail. Tablet counts will be made of all returned medication packs.

Records will be maintained of the product's delivery to each ASPREE site, the use by each participant, and the disposition of unused study medication. These records will include dates, quantities, batch numbers, expiry dates and the unique Medication ID numbers assigned to the investigational product(s) and study participants.

Investigators will maintain records that document adequately that study participants were provided the doses specified by the protocol and reconcile all study medication received from the drug distribution center. The investigator must verify that all unused or partially used drug supplies have been returned by the study participant and that no remaining supplies are in the participant's possession. Accurate drug records will be achieved through the use of Dispensing Logs.

8.7 Drug disposal

Annually and at the end of the study all packages of medication, whether empty or containing unused drugs collected by study staff, will be disposed of according to local regulatory requirements.

8.8 Emergency code breaking

In the event of a clinical emergency, the participant will be assumed to be taking active therapy and recommended to cease treatment. The code can be broken by contacting the Monash Clinical Informatics and Data Management Unit to handle emergency un-blinding in Australia and the U.S. This procedure will ensure that no-one involved in the management or conduct of the study will have access to the randomization code. Reasons for the un-blinding will be recorded at the Unit and participants will be encouraged to resume their assigned medication if possible after their immediate condition has resolved. Details will be recorded and passed to the ASPREE DMC. The DSMB will also be informed of any emergency code breaks in tabular form. The code will be broken for an individual in the event where knowledge of the precise medication is essential for the clinical management and where this is requested by the treating physician (or the participant). Blinding will be protected by this systematic approach.

8.9 Overdose of trial medication

A procedure in case of overdose with aspirin is to be specified. The signs of acute intoxication of ASA are:

- Mild: Nausea, vomiting, sweating, thirst and tachycardia.
- Severe: Fever, CNS disturbances such as convulsions, hallucinations, coma, and respiratory failure.

Treatment: The initial action is to eliminate the swallowed tablets from the gut by gastric washout and administration of activated charcoal for mild cases (i.e. where no depression of conscious state) and transport to a specialized center. Severe cases require emergency transport.

In the event of overdose of study medication, a decision on continuing in the unblinded study will be reviewed. The safety officer may be asked to decide if the patient status is to remain unblinded.

9 CONCOMITANT THERAPY

9.1 Prescription medications

All prescription medications will be recorded on a *Concomitant Medications* Form including indication for use and start / stop dates. In Australia, family physician records will be checked for the participant's concomitant medications. In the U.S., at study entry and at each annual visit participants will be asked to bring in all of the medications that they currently use or have recently used, including over–the-counter medications (the 'brown bag' method), to verify their current medications.

In addition to prescription medications, any regular use of over-the-counter NSAIDs will also be recorded on the *Concomitant Medications* Form.

10 SAMPLE SIZE AND POWER CALCULATIONS

Participants will be approximately two-thirds from Australia and one-third from the U.S and will include 4,500 minority participants. There will be an equal allocation of participants to aspirin and placebo groups. A detailed description of sample size and power calculations for the primary and secondary endpoints is provided in Appendix 2.

In brief, 19,000 participants having 3787 events will be required to have 90% power (two-sided alpha error rate of 0.05) to detect a 10% reduction (risk ratio of 0.90) in the primary endpoint with aspirin. The rationale for this effect size is described in Appendix 2. This sample size was calculated for a univariate Cox proportional hazards regression analysis[97] using Stata (Stata Statistical Software, Release 10, StataCorp, College Station, TX, 2007) and based on the following assumptions:

a. The effect of aspirin is described by a hazard ratio of 0.90 in an intention-to-treat analysis. This assumes a slightly stronger underlying effect that will be weakened by cross-over of participants for reasons which include the development of a non-fatal, non-disabling cardiovascular or cerebrovascular events necessitating aspirin therapy or cessation (non-compliance) with aspirin therapy. We expect 5% per annum of placebo-group participants to initiate aspirin use (or *vice versa*).

b. Annual dementia incidence rates of 6 per 1000 for 70-74y age group, 11/1000 (75-79y), 20/1000 (80-84y), 37/1000 (85y+) [98 99] for Australian, U.S. Hispanic and U.S. White (non-Hispanic) participants. For U.S. African Americans the rates are doubled [99 100].

c. Incident disability for one or more Katz ADL's; annual incidence rate for males= 19.6/1000, females = 26.5/1000 (these incidence rates are from the rates observed in the subset of

ASPREE-like participants in the CHS (Newman A., personal communication, August, 2008) which showed similar rates in U.S. Whites and U.S. minorities).

d. Age-specific mortality rates in Australian and U.S. White (non-Hispanic) participants are based on the 2004 Australian population census (http://www.aihw.gov.au/mortality/data). Age-specific mortality rates in U.S. African American and Hispanic participants are based on 2005 rates specific to each minority (U.S. CDC statistics, 2005; http://www.cdc.gov/nchs).

e. To allow for the analysis to be time-to-first event, individual rates for death, ADL loss and dementia can be summed and the sum reduced by 10% to allow for the potential for different events to occur in the same individual [40].

f. Participants will be 45% male and 55% female.

g. The cohort will consist of 50%, 30%, 15%, 5% in age groups 70-74 years, 75-79 years, 80-84 years, 85 years and over, respectively.

h. The average at risk time will be 4.25 years per participant which allows for censoring due to the primary endpoint or non-completion of dementia screen or diagnosis, and non-completion of ADLs.

11 ASSESSMENT AND REPORTING OF ADVERSE EVENTS

11.1 Adverse Events

At the designated intervals for event collection, participants will be asked about specific events of interest as well as any new diseases or conditions.

AEs will be obtained by self-report and will not require supporting documentation. AEs will be reported quarterly to the DSMB and the NIA by blinded treatment group.

11.2 Serious Adverse Events (SAE)

Serious adverse events (SAE) or Serious Adverse Drug Reaction (Serious ADR) are defined as any untoward medical occurrence that:

- is fatal,
- is life-threatening (see below),
- requires in-patient hospitalization or prolongation of existing hospitalization (see below), or
- causes persistent or significant disability / incapacity, or
- is an accidental or intentional overdose.

An SAE is considered life threatening when this places the participant at immediate risk of death from the event as it occurred. A life-threatening event does not include an event that might have caused death had it occurred in a more severe form but that did not create an immediate risk of death as it actually occurred. For example, drug-induced hepatitis that resolved without evidence of hepatic failure would not be considered life threatening, even though drug-induced hepatitis of a more severe nature can be fatal.

A hospitalization is to be considered an SAE only if it is an official admission with a duration of more than 24hr or a minimum of 2 calendar days where exact time of stay is unavailable.

SAEs will be reported from the study sites directly to the DMC within 24 hours of their detection. DMC will compile the details of such events and present the report to the DSMB and the NIA as follows:

1. All final reports of deaths will be provided when available to NIA and a safety monitor.
2. All SAEs shall be reported in a summary format by the blinded treatment arm (A and B) to the DSMB and NIA monthly.

12 ADHERENCE TO ETHICAL, REGULATORY AND ADMINISTRATIVE CONSIDERATIONS

12.1 Ethical Considerations

12.1.1 General

This study will be conducted in accordance with the Declaration of Helsinki 1964 as revised in Edinburgh in 2000 and with the National Health & Medical Research Council Guidelines on Human Experimentation.

In Australia, the Royal Australasian College of General Practitioners Ethics Committee (ethics #NREEC 02/22b), the Monash University Human Research Ethics Committee (**IRB00002519**; ethics #2006/745MC), the Human Research Ethics Committee (Tasmanian) Network (ethics #H0008933), the Goulburn Valley Health Ethics & Research Committee (Shepparton, ethics #GVH-21/07),ACT Health Human Research Ethics Committee (Canberra, ethics #11/07.997) and The University of Adelaide Human Research Ethics Committee (ethics #H-250-2011) have previously approved the study. In the U.S., individual clinic sites will be responsible for obtaining IRB approval from their respective institutions prior to study initiation. Summary reports of the deliberations of the DSMB will be transmitted to all relevant IRBs and Ethics Review committees

that have approved and are monitoring the study. Study investigators will be responsible for forwarding reports to their relevant IRBs and Ethics Committees.

12.1.2 Information for Participants

Before obtaining consent from the participant they must be informed of the objectives, benefits, risks and requirements of the study, as well as the nature of the test medication. A participant consent form should be given to every participant prior to screening.

12.1.3 Informed Consent

a) All participants must give their informed consent **before** screening and randomization.
b) In the event that a proxy report of ADL or IADL is necessary, such as in the case of dementia assessment, the person previously nominated as proxy or 'surrogate' (e.g. partner, adult offspring, family physician) will be asked to provide consent before he or she completes the questionnaires.
c) Two copies of the consent form are to be made, one for the participant (or proxy) and one for the investigators to be stored in the participant's individual file.
d) Informed consent is obtained by the site investigator and/or by the research staff from the participant (or proxy). The ASPREE research staff should fully inform the participant of all pertinent aspects of the ASPREE study by reviewing the study information and consent form. All participants should be informed to the fullest extent possible about the study, in language and terms they are able to understand.
e) Prior to a participant involvement in the study, the written Informed Consent Form should be signed, name filled in and personally dated by the participant and by the person who conducted the informed consent discussion. A copy of the signed and dated written Informed Consent Form will be provided to the participant. The original consent form is to be stored in the participant's individual study file, held by the investigator. In Australia, a second copy may be filed in the participant's file at the general practice.
f) The form used for obtaining the participant's informed consent must be the current version that has been reviewed and approved by the appropriate IRB or Ethics Committee.

12.2 Regulatory Considerations

12.2.1 Financing

The feasibility trial was supported by the National Heart Foundation of Australia. Enteric coated aspirin and matching supply and packaging will be provided by Bayer Pharma AG. ASPREE

has received project grants from the National Institute of Aging (part of the National Institutes of Health), the National Health and Medical Research Council (Australia), the National Heart Foundation of Australia, the Victorian Cancer Agency and an educational grant from Bayer Pharma AG.

12.2.2 Trial registration

ASPREE is registered on the International Standard Randomized Controlled Trial Number Register (ISRCTN83772183). ASPREE will be registered with ClinicalTrials.gov.

12.2.3 Disclosure of Conflict of Interest

Full disclosure by all of the key members of the study of their, and their immediate family's, financial relationships with all pharmaceutical and biomedical companies judged to have an active or potential interest in the conduct and outcome of the study will be made annually. Details and conditions of this disclosure are included in Appendix 3.

12.3 Administrative Organization

12.3.1 Individual and Committee responsibilities

Principal Investigators (Richard Grimm and John McNeil)

The ASPREE Steering Committee will be responsible for the overall management and conduct of ASPREE including finalizing the protocol, approving the operational plan for the study, and financial management of the trial. The committee will be chaired by Richard Grimm and John McNeil, on an annual rotation as Chair. The committee will consist of the Co-Chairs, the International Operations Committee (see below), the Principal Investigator from each of the recruiting Hubs in the U.S. and Australia plus individuals with Special Content Expertise (SCE) who may be invited to join the Steering Committee. The Steering Committee will plan to meet face-to-face annually with conference calls as needed, but at least every 6 months.

International Operations Committee (IOC) is a smaller committee derived from the Steering Committee. It will consist of the Co-Chairs of the Steering Committee, two representatives from the U.S. Operations Committee, two representatives from the Australian Operations Committee and the Chair of the Data Management Committee. The IOC will meet by teleconference, as needed, or face-to-face at the annual Steering Committee meeting. This smaller committee must be prepared to meet at short notice if any urgent issues related to the study should arise.

National Operations Committees will be established in both countries. Each committee will be responsible for implementing the study, monitoring the progress of recruitment and ensuring

adherence and standardization throughout the study in that country. Each committee will also work to ensure co-ordination, consistency, transfer of data to the Data Management Center and quality control. They will ensure quality control review of laboratory data, clinical measurements and data collection, completeness and entry times of data, site monitoring and source documentation. Each committee will communicate regularly with each other (4-6 teleconferences per year) to ensure consistency of protocol implementation across countries. These Committees may consist of site Principal Investigators or Project Managers and key research personnel and will report directly to the International Steering Committee.

Recruitment, Adherence & Audit Committee: This committee will monitor the progress of recruitment against targets, and if necessary institute remedial actions. It will also review the rates of adherence to randomized treatments and the data from the quality assurance program.

Data Management Committee: This committee will meet twice a year in order to monitor the establishment and implementation of the ASPREE Data Management System, the reporting activities of the DMC and to review all quality assurance reports for the study. The DMC Chair will report to the Steering Committee of ASPREE on Data Management Issues associated with the study.

Data Safety and Monitoring Board (DSMB): This committee will be appointed by the NIA and will adhere to the DSMB Charter. It is anticipated that the DSMB will provide recommendations to the Steering Committee in relation to subject recruitment, event rate tracking, monitoring adverse events and conducting interim analyses.

The DSMB will have responsibility for monitoring quality control of the data, progress of recruitment and safety aspects of the trial. The DMC will present data for review by the DSMB by a blinded treatment arm (e.g., ‘A’ and ‘B’). The DSMB will also be responsible for responding to the interim analysis on unblinded data and will review deaths, serious adverse events and other endpoint data on a periodic basis. The DSMB reports will be passed to the NIA. Administrative support will be provided by an independent biostatistician and research assistant.

The DSMB will be provided with data every 6 months or as requested. The DSMB will meet every 6 months, alternating by teleconference or in-person to review study progress, data quality control, address policy issues and review total mortality data, adverse events and all safety data and monitor the study progress and data quality. The DSMB will provide a follow-up report and recommendations to the NIA. The DSMB has expertise related to the conduct of clinical trials *per se* and in the primary care sector, epidemiology, biostatistics, clinical pharmacology, clinicians, and cardiovascular disease. Confidentiality will be maintained during all phases of the

trial including monitoring, preparation of interim results, review, and response to monitoring recommendations.

Endpoint Adjudication Committee: This committee will evaluate individual outcomes blinded to randomized treatment. Each endpoint will be validated through examination of relevant clinical information. Specialist sub-committees will be appointed, such as the Dementia Adjudication Panel, to adjudicate on specific endpoints with a nominated person as member of the EAC.

Publications, Presentations and Ancillary Studies Committee: This committee will be responsible for implementation of the publication policy (12.3.3), coordination of topics and requests for publication, approval for abstracts and submission to conferences, and receipt of proposals for ancillary studies (12.3.4).

Dissemination Committee: One year prior to when the ASPREE trial is scheduled to end a Dissemination Committee will be constituted in both countries to begin to plan the dissemination in both the U.S. and Australia. This effort will be modeled after the pioneer and successful effort in the ALLHATT study in which Dr Grimm participated.

General Practice Advisory Committee (Australia only): To provide input into the National Operations Committee and others on aspects of the trial related to family practice in Australia.

12.3.2 Data Safety and Monitoring Plan

General monitoring of the study will be provided by the Principal Investigators (Richard Grimm in the U.S. and John McNeil in Australia) and the Data Safety Monitoring Board (for the DSMB Charter see Appendix 4). A Data and Safety Monitoring Plan (DSMP) for ASPREE has been prepared according to the DSMP Guidelines for Clinical Trials provided by the NIA and must be approved by the DSMB.

Formal stopping rules are described in detail in the DSMP. Since one of the principal goals of ASPREE is to evaluate the balance of benefits and risks of low-dose aspirin an important consideration for the DSMB will be all-cause mortality comparisons by study groups, in addition to the primary endpoint. The DSMB could decide to terminate the trial if an important difference in total mortality is observed. Another important consideration is the possibility of event rates being lower than expected requiring an assessment of study futility.

12.3.3 Publication policy

This policy covers all publications and abstracts originating from ASPREE and any sub-study. The report of the paper will follow the CONSORT (Consolidated Standards of Reporting Trials)

guidelines for reporting randomized controlled trials. [101]

Authorship. - Manuscripts and abstracts relating to the ASPREE study must include all current members of the Steering Committee using the following formula:

- All publications will be on behalf of 'the ASPREE Study Group'.
- A writing committee will be established for each publication from which a lead author will be identified and responsible for the initial draft of the manuscript
- The lead author will be the first author of the publication
- Subsequent author(s) from the writing committee will be listed according to the amount of input to the writing of the paper.
- All other contributors in last name alphabetical order.
- Members of the ASPREE Steering Committee will be named in description of the ASPREE Study Group in each manuscript
- All clinical site investigators and committee members will be listed on the ASPREE web-site and acknowledged in every publication.

Non-Steering Committee authors utilize the same formula. Disputes about authorship must be notified to the Principal Investigator(s) to be resolved at the next Steering Committee meeting.

Drafts -Initial and major upgraded manuscripts and abstracts must be circulated to all members of the Steering Committee and any other Committee within ASPREE when appropriate. Members will have a maximum of one week to send responses.

12.3.4 Ancillary studies

Ancillary studies must be submitted to and approved by the Steering Committee. They are subject to independent sources of funding being procured and must not impact adversely on the main goals and conduct of the trial. Applications should be made to the Steering Committee and submitted by a Principal Investigator with a maximum of a five page summary of the rationale and method and must include a budget and evidence of funding or a strategy for securing said. The Steering Committee must ensure that any ancillary study will not compromise the main study.

13 DATA MANAGEMENT

13.1 Data Handling and Record Keeping

All the results from evaluations conducted during the trial will be recorded on an appropriate

CRF for each participant and filed by the relevant ASPREE clinical site. The data from the CRFs will be uploaded onto the ASPREE web-based portal using the electronic CRFs provided.

Full identification of each participant will be kept by the local GP investigator / research staff (in Australia) or the local site investigator / research staff (in the U.S.) who agree to supply all details to the auditor and/or the Regulatory Authorities if required. All information will be treated in accordance with professional conduct. All corrections and alterations of data on the CRFs must be made according to the instructions provided and must be dated and initialed (see Section 7.2). The CRFs must be completed during or after each participant visit (or as soon as all data is available, e.g. once pathology results are obtained) and uploaded onto the ASPREE web-based portal.

Data Collection and Management will be centralized at the ASPREE DMC, Department of Epidemiology and Preventive Medicine, Monash University, Melbourne. Study data collected at all clinical sites will be entered on electronic CRFs with source documentation retained in the participant file. All information related to potential endpoints will be copied from the original documents sourced through clinical notes and hospital medical records and sent de-identified to the ASPREE DMC within 90 days of the event being discovered or reported. Serious adverse events must be entered within 24 hours of receipt from sites. Serious adverse event and endpoint data will be accepted as either a) photocopied clinical records or b) scanned or photographed image files of clinical records. The clinic will retain all original information related to the clinical event or endpoint in the subject files.

Electronic study records will be accessible through the internet at the ASPREE web-portal. Access to this web-site will be made available to registered study staff via password protected log-in procedures. Each DMC staff member is trained in data management and is highly experienced in data management for large-scale clinical trials. All staff involved in data collection for ASPREE will participate in a training program to enable familiarization with all procedures associated with the web-based data collection.

13.2 Quality Control

The ASPREE quality assurance plan, based on methods described in [102-105] has been designed to ensure that (a) study staff are fully trained and their performance monitored, (b) all subjects recruited to the study meet the required entry criteria, (c) there is full compliance with the study protocol and ethical requirements, (d) study participants are receiving the medication to which they have been assigned, (e) data collected is complete and accurate, and (f) other procedures (e.g. data backup and record storage) are being conducted in accordance with study

requirements.

All staff assigned to ASPREE will attend an initial training meeting covering good research practice, clinical research ethics and relevant trial procedures. These procedures will be shared in the form of Standard Operating Procedures (SOPs) that will describe in detail descriptions of all study procedures. In addition, study forms (CRF's) will be required for completion for each visit. All of these materials will be made available via the ASPREE website. Training will occur in each country and will be coordinated by each respective Coordinating Center. Trial personnel in supervisory positions will be involved with the training to ensure consistency of procedures between staff in both countries. In addition to the initial study training, ongoing competency will be monitored regarding the various cognitive testing that will be administered by site staff. A "certification" process will be in place to maintain a high level of testing quality.

The DMC will be responsible for data editing that will include checks for missing data and inconsistencies. Site staff will be responsible for completing data edits within a reasonable period of time. The DMC will track quality control measures in a quality control report that will be reviewed by the Joint Operations Committee and the International Steering Committee on a regular basis. Such measures will include, but are not limited to, number of participants with missed or late visits, portion of study participants off blinded medication, documentation for reported study events, portion of participants with labs, and deviations from the protocol.

The progress of recruitment will be monitored by plotting actual *versus* expected enrolment and this information will be provided weekly to the members of the Recruitment, Retention and Audit Committee. If the rate of recruitment falls below the required rate, responses may include an increase in the number of recruitment centers and/or an increase in promotional activities.

A random selection of participants (10% over the duration of the trial) will be selected by the DMC for review by a site monitor/auditor on an annual basis. Usual activities at site monitoring visits may include reviews of clinic staffing levels and duties, discussions of clinic flow, inspections of clinic space and records, review of study drug accountability, reviews of the various reports from the DMC, reviews of maintenance logs for important study equipment, confirmation of participant's consents, inclusion and exclusion criteria, source documentation, presence of regulatory documents, review of recruitment and adherence strategies and trouble-shooting problems. A check list for monitoring visits will be completed by each monitor/auditor for each visit. A report will then be provided to the site PI and key staff (to be retained with their regulatory documents), and the respective US/AU Coordinating Center.

14 REFERENCES

1. EUROASPIRE I and II Group. Clinical reality of coronary prevention guidelines: a comparison of EUROASPIRE I and II in nine countries. European Action on Secondary Prevention by Intervention to Reduce Events. *Lancet* 2001;357(9261):995-1001.
2. May GS, et al. Secondary prevention after myocardial infarction: a review of long-term trials. *Prog Cardiovasc Dis* 1982;24(4):331-352.
3. Troche CJ, et al. Cost-effectiveness of primary and secondary prevention in cardiovascular diseases. *Eur Heart J* 1998;19 Suppl C:C59-65.
4. Jonker C, et al. Does aspirin or other NSAIDs reduce the risk of cognitive decline in elderly persons? Results from a population-based study. *Neurobiol Aging* 2003;24(4):583-588.
5. Patrono C, et al. Low-dose aspirin for the prevention of atherothrombosis. *N Engl J Med* 2005;353(22):2373-2383.
6. Hennekens CH, et al. Sex-related differences in response to aspirin in cardiovascular disease: an untested hypothesis. *Nat Clin Pract Cardiovasc Med* 2006;3(1):4-5.
7. Eikelboom JW, O'Donnell M. Using aspirin to prevent cognitive decline. *Brit Med J* 2008;337:a958.
8. Scheinberg P. Dementia due to vascular disease - a multifactorial disorder. *Stroke* 1988;19:1291-1299.
9. Andrawes WF, et al. Prevention of cardiovascular events in elderly people. *Drugs Aging* 2005;22:859-876.
10. Hayden M, et al. Aspirin for the primary prevention of cardiovascular events: a summary of the evidence for the U.S. Preventive Services Task Force. *Ann Intern Med* 2002;136(2):161-172.
11. Antithrombotic Trialists' Collaboration. Collaborative meta-analysis of randomised trials of antiplatelet therapy for prevention of death, myocardial infarction, and stroke in high risk patients. *Brit Med J* 2002;324(7329):71-86.
12. Steering Committee of the Physicians' Health Study Research Group. Final report on the aspirin component of the ongoing Physicians' Health Study. *New Engl J Med* 1989;321(3):129-135.
13. Peto R, et al. Randomised trial of prophylactic daily aspirin in British male doctors. *Brit Med J (Clin Res Ed)* 1988;296(6618):313-316.
14. Hansson L, et al. Effects of intensive blood-pressure lowering and low-dose aspirin in patients with hypertension: principal results of the Hypertension Optimal Treatment (HOT) randomised trial. HOT Study Group. *Lancet* 1998;351(9118):1755-1762.
15. The Medical Research Council's General Practice Research Framework. Thrombosis Prevention Trial (TPT): randomised trial of low-intensity oral anticoagulation with warfarin and low-dose aspirin in the primary prevention of ischaemic heart disease in men at increased risk. *Lancet* 1998;351(9098):233-241.
16. Ridker PM, et al. A randomized trial of low-dose aspirin in the primary prevention of cardiovascular disease in women. *N Engl J Med* 2005;352(13):1293-1304.
17. Collaborative Group of the Primary Prevention Project. Low-dose aspirin and vitamin E in people at cardiovascular risk: a randomised trial in general practice. *Lancet* 2001;357(9250):89-95.
18. Baigent C. Personal communication, 2004.
19. Berger JS, et al. Aspirin for the primary prevention of cardiovascular events in women and men: a sex-specific meta-analysis of randomized controlled trials. *J Am Med Assoc* 2006;295(3):306-313.
20. Antithrombotic Trialists (ATT) Collaboration, *et al*. Aspirin in the primary and secondary prevention of cardiovascular disease: collaborative meta-analysis of individual participant

data from randomised trials. *Lancet* 2009;373:1849-1860.

21. Nelson MR, et al. Epidemiological modelling of routine use of low dose aspirin for the primary prevention of coronary heart disease and stroke in those aged > or =70. *Brit Med J* 2005;330(7503):1306-1311.

22. Fleming T, et al. Report from the 100th Cardiovascular and Renal Drugs Advisory Committee meeting: US Food and Drug Administration: December 8-9, 2003 Gaithersburg, MD. *Circulation* 2004;109(2):e9004-9005.

23. Sturmer T, et al. Aspirin use and cognitive function in the elderly. *Am J Epidemiol* 1996;143(7):683-691.

24. McGeer PL, et al. Arthritis and anti-inflammatory agents as possible protective factors for Alzheimer's disease: a review of 17 epidemiologic studies. *Neurology* 1996;47(2):425-432.

25. Broe GA, et al. Anti-inflammatory drugs protect against Alzheimer disease at low doses. *Arch Neurol* 2000;57(11):1586-1591.

26. in t' Veld BA, et al. Nonsteroidal antiinflammatory drugs and the risk of Alzheimer's disease. *N Engl J Med* 2001;345(21):1515-1521.

27. Mukherjee D, et al. Risk of cardiovascular events associated with selective COX-2 inhibitors. *J Am Med Assoc* 2001;286(8):954-959.

28. Topol EJ. Failing the public health--rofecoxib, Merck, and the FDA. *N Engl J Med* 2004;351(17):1707-1709.

29. Ross R. Atherosclerosis--an inflammatory disease. *N Engl J Med* 1999;340(2):115-126.

30. Kopp E, Ghosh S. Inhibition of NF-kappa B by sodium salicylate and aspirin. *Science* 1994;265(5174):956-959.

31. Brookmeyer R, et al. Projections of Alzheimer's disease in the United States and the public health impact of delaying disease onset. *Am J Public Health* 1998;88(9):1337-1342.

32. Price JF, *et al.* Low dose aspirin and cognitive function in middle aged to elderly adults: randomised controlled trial. *Brit Med J* 2008; 337:a1198.

33. de Craen AJM, et al. Meta-analysis of nonsteroidal antiinflammatory drug use and risk of dementia. *Am J Epidemiol* 2005;161(2):114-120.

34. Kang JH, et al. Low dose aspirin and cognitive function in the women's health study cognitive cohort. *Brit Med J* 2007;334(7601):987-994.

35. Whalley LJ, Mowat DHR. Aspirin and cognitive function. *Brit Med J* 2007;334(7601):961-962.

36. Guralnik JM, *et al*. Morbidity and disability in older persons in the years prior to death. Am J Pub Health 1991; 81:443-447.

37. Katz S, *et al*. Studies of illness in the aged. The index of ADL: a standardized measure of biological and psychosocial function. *J Am Med Assoc* 1963; 185: 94-99.

38. Newman A, et al. Association of long-distance corridor walk performance with mortality, cardiovascular disease, mobility limitation, and disability. *J Am Med Assoc* 2006;295(17):2018-2026.

39. Kuller LH, et al. Relationship between ApoE, MRI findings, and cognitive function in the Cardiovascular Health Study. *Stroke* 1998;29(2):388-398.

40. Rosano C, et al. Subclinical brain magnetic resonance imaging abnormalities predict physical functional decline in high-functioning older adults. *J Am Geriatr Soc* 2005;53(4):649-654.

41. Guralnik JM, et al. Lower-extremity function in persons over the age of 70 years as a predictor of subsequent disability. *N Engl J Med* 1995; 332(9):556-562.

42. Perera S, et al. Meaningful change and responsiveness in common physical performance measures in older adults. *J Am Geriatr Soc* 2006; 54(5):743-749.

43. Cesari M, et al. Prognostic value of usual gait speed in well-functioning older people--results from the Health, Aging and Body Composition Study. *J Am Geriatr Soc* 2005; 53(10):1675-1680.

44. Newman AB, et al. Strength, but not muscle mass is associated with mortality in the Health ABC Cohort. *J Gerontol A Biol Sci Med Sci* 2006; 61A(1):M72-M77.
45. Guralnik JM, et al. Disability as a public health outcome in the aging population. *Ann Rev Pub Health* 1996. 17:25-46.
46. Harris RE, et al. Aspirin, ibuprofen, and other non-steroidal anti-inflammatory drugs in cancer prevention: a critical review of non-selective COX-2 blockade (review). *Oncol Rep* 2005;13(4):559-583.
47. Takkouche B, et al. Breast cancer and use of nonsteroidal anti-inflammatory drugs: a meta-analysis. *J Nat Cancer Insti,* 2008; 100:1420-1423.
48. Bardia A, et al. Association of Aspirin and Nonaspirin Nonsteroidal Anti-inflammatory Drugs With Cancer Incidence and Mortality. *J Natl Cancer Inst* 2007;99(11):881-889.
49. Cook NR, et al. Low-dose aspirin in the primary prevention of cancer: the Women's Health Study: a randomized controlled trial. *J Am Med Assoc* 2005;294(1):47-55.
50. Australian Institute of Health and Welfare. *Cancer in Australia 2000.* Canberra, 2003.
51. Dube C, et al. The use of aspirin for primary prevention of colorectal cancer: a systematic review prepared for the U.S. Preventive Services Task Force. *Ann Intern Med* 2007;146(5):365-375.
52. Baron JA, et al. A randomized trial of aspirin to prevent colorectal adenomas. *N Engl J Med* 2003;348(10):891-899.
53. Sandler RS, et al. A randomized trial of aspirin to prevent colorectal adenomas in patients with previous colorectal cancer. *N Engl J Med* 2003;348(10):883-890.
54. Chan AT, et al. Long-term use of aspirin and nonsteroidal anti-inflammatory drugs and risk of colorectal cancer. *J Am Med Assoc* 2005;294(8):914-923.
55. Chan AT, et al. Aspirin and the risk of colorectal cancer in relation to the expression of COX-2. *N Engl J Med* 2007;356(21):2131-2142.
56. Flossmann E, Rothwell PM. Effect of aspirin on long-term risk of colorectal cancer: consistent evidence from randomised and observational studies. *Lancet* 2007;369(9573):1603-1613.
57. Gann PH, et al. Low-dose aspirin and incidence of colorectal tumors in a randomized trial. *J Natl Cancer Inst* 1993;85(15):1220-1224.
58. Chan AT, et al. Long-term aspirin use and mortality in women. *Arch Intern Med* 2007;167:562-572.
59. Ulrich CM, et al. Non-steroidal anti-inflammatory drugs for cancer prevention: promise, perils and pharmacogenetics. *Nature Reviews / Cancer,* 2006; 6:130-140.
60. Murray CJ, et al. Disability-adjusted life years (DALYs) for 291 diseases and injuries in 21 regions, 1990-2010: a systematic analysis for the Global Burden of Disease Study 2010. *Lancet* 2013;380(9859):2197-223.
61. Collishaw S, et al. Time trends in adolescent mental health. *J Child Psychol Psychiatry*. 2004;45(8):1350-62.
62. Twenge JM, et al. Birth cohort increases in psychopathology among young Americans, 1938-2007: A cross-temporal meta-analysis of the MMPI. *Clin Psychol Rev.* 2010;30(2):145-54.
63. Penninx BW, et al. Inflammatory markers and depressed mood in older persons: results from the Health, Aging and Body Composition study. *Biol Psychiatry.* 2003;54(5):566-72
64. Pasco JA, et al. Association of high-sensitivity C-reactive protein with de novo major depression. *Br J Psychiatry.* 2010;197(5):372-7.
65. Berk M, et al. Aspirin: A review of its neurobiological properties and therapeutic potential for mental illness. *BMC Medicine.* 2013, 11:74
66. Hernandez-Diaz S, Rodriguez LA. Incidence of serious upper gastrointestinal bleeding/perforation in the general population: review of epidemiologic studies. *J Clin Epidemiol* 2002;55(2):157-163.

67. Silagy CA, et al. The PACE pilot study: 12-month results and implications for future primary prevention trials in the elderly. (Prevention with low-dose Aspirin of Cardiovascular disease in the Elderly). *J Am Geriatr Soc* 1994;42(6):643-647.
68. Nelson M, et al. Rationale for a trial of low-dose aspirin for the primary prevention of major adverse cardiovascular events and vascular dementia in the elderly: Aspirin in Reducing Events in the Elderly (ASPREE). *Drugs Aging*, 2003; 20(12):897-903.
69. Thrift AG, et al. Risk of primary intracerebral haemorrhage associated with aspirin and non-steroidal anti-inflammatory drugs:case-control study. *Brit Med J* 1999;318(7186):759-764.
70. He J, et al. Aspirin and risk of hemorrhagic stroke: a meta-analysis of randomized controlled trials. *J Am Med Assoc* 1998;280(22):1930-1935.
71. Australian Institute of Health and Welfare. Heart, stroke and vascular disease - Australian facts 2001. *AIHW cat. no. CVD13*. Canberra: National Heart Foundation of Australia, National Stroke Foundation of Australia, 2001.
72. National Center for Health Statistics, United States, 2007. *Chartbook on Trends in the Health of Americans*; Table 29 Hyattsville, MD: 2007. Library of Congress Catalog Number 76-641496.
73. Australian Institute of Health and Welfare. Mortality over the twentieth century in Australia. Trends and patterns in major causes of death. *AIHW cat. no.* PHE 73, 2006.
74. National Projections Program, Population Division, U.S. Census Bureau, Washington, D.C. 20233.
75. Katz S, Apkon CA. A measure of primary sociobiological functions. *Int J Health Serv* 1976;6:493-507.
76. Teng EL, Chui HC. The Modified Mini-Mental State (3MS) examination. *J Clin Psychiatry* 1987;48(8):314-318.
77. Radloff LS. The CES-D scale: A self report depression scale for research in the general population. *Appl Psychol Meas* 1977;1:385-401.
78. Smith A. Symbol Digit modalities Test (SDMT). Manual (Revised). Western Psychological Services, Los Angeles, 1982.
79. Brandt J, Benedict RHB. Hopkins Verbal Learning Test - Revised. Ed: PAR., Odessa, Fla., 2001.
80. Ross TP. The reliability of cluster and switch scores for the Controlled Oral Word Association Test. *Arch Clin Neuropsychol* 2003;18(2):153-164.
81. Pahor M, et al. Effects of a physical activity intervention on measures of physical performance: results of the Lifestyle Interventions and Independence for Elders Pilot (LIFE-P) Study. *J Gerontol A Biol Sci Med Sci* 2006;61(11):1157-1165.
82. Ware J, et al. A 12-Item Short-Form Health Survey: construction of scales and preliminary tests of reliability and validity. *Med Care* 1996;34(3):220-233.
83. Reid CM, et al. General practitioner participation in the second Australian National Blood Pressure Study (ANBP2). *Clin Exp Pharmacol Physiol* 2001;28(8):663-667.
84. Reid CM, et al. A new paradigm for funding cardiovascular-outcome research in general practice. The Second Australian National Blood Pressure Study. ANBP2 Management Committee. *Med J Aust* 1998;169(7):349-350.
85. Wing LM, et al. Second Australian National Blood Pressure Study (ANBP2). Australian Comparative Outcome Trial of ACE inhibitor- and diuretic-based treatment of hypertension in the elderly. Management Committee on behalf of the High Blood Pressure Research Council of Australia. *Clin Exp Hypertens* 1997;19(5-6):779-791.
86. Wing LM, et al. comparison of outcomes with angiotensin-converting--enzyme inhibitors and diuretics for hypertension in the elderly. *N Engl J Med* 2003;348(7):583-592.
87. Guralnik J, et al. Lower extremity function and subsequent disability: consistency across studies, predictive models, and value of gait speed alone compared with the short physical

performance batter. *J Gerontol A Biol Sci Med Sci* 2000;55A:M221-M231.

88. Onder G, et al. Change in physical performance over time in older women: the Women's Health and Aging Study. *J Gerontol A Biol Sci Med Sci* 2002;57(5):M289-M293.

89. Grimm RH, Jr, et al. Baseline Characteristics of Participants in the Antihypertensive and Lipid Lowering Treatment to Prevent Heart Attack Trial (ALLHAT). *Hypertension* 2001;37(1):19-27.

90. ALLHAT Group. Major outcomes in high-risk hypertensive patients randomized to angiotensin-converting enzyme inhibitor or calcium channel blocker vs diuretic: The Antihypertensive and Lipid-Lowering Treatment to Prevent Heart Attack Trial (ALLHAT). *J Am Med Assoc* 2002;288(23):2981-2997.

91. Furberg CD, et al. Major cardiovascular events in hypertensive patients randomized to doxazosin versus chlorthalidone: in Antihypertensive and Lipid Lowering Treatment to Prevent Heart Attack Trial (ALLHAT). *J Am Med Assoc* 2000;283(15):1967-1975.

92. ALLHAT Study Group. The Antihypertensive and Lipid-Lowering Treatment to Prevent Heart Attack Trial (ALLHAT). Major outcomes in moderately hypercholesterolemic, hypertensive patients randomized to pravastatin vs usual care. *J Am Med Assoc* 2002;288(23):2998-3007.

93. Piller LB, et al. Validation of heart failure events in the Antihypertensive and Lipid Lowering Treatment to Prevent Heart Attack Trial (ALLHAT) participants assigned to doxazosin and chlorthalidone. *Curr Cont Trials Cardiovasc Med* 2002;3(1):10.

94. Schulz KF, Grimes DA. Multiplicity in randomized trials I: endpoints and treatments. *Lancet* 2005;365:1348-1353.

95. Sankoh A, et al. Efficacy endpoint selection and multiplicity adjustment methods in clinical trials with inherent multiple endpoint issues. *Stat Med* 2003;22(20):3133-3150.

96. Molenberghs G, Kenward MG. *Missing Data in Clinical Studies*. Chichester, 2007.

97. StataCorp. Survival Analysis and Epidemiological Tables Manual for Stata Statistical Software: Release 9.0. . TX: College Station, Stata Corporation, 2006.

98. Kawas CH, Katzman R. *Epidemiology of Dementia and Alzheimer Disease. In:Alzheimer Disease 2nd Ed, pp.95-116*. 2nd ed. Philadelphia: Lippincott Williams & Wilkins, 1999.

99. Tang MX, et al. Incidence of AD in African-Americans, Caribbean Hispanics, and Caucasians in northern Manhattan. *Neurology* 2001;56(1):49-56.

100. Hendrie HC, et al. Incidence of Dementia and Alzheimer Disease in 2 Communities: Yoruba Residing in Ibadan, Nigeria, and African Americans Residing in Indianapolis, Indiana. *J Am Med Assoc* 2001;285(6):739-747.

101. Moher D, et al. The CONSORT statement: revised recommendations for improving the quality of reports of parallel-group randomised trials. *Clin Oral Investig* 2003;7(1):2-7.

102. Ronel RK, *Clinical Data Management* ed. RK Ronel, SA Varley and CF Webb. 2000; John Wiley & Sons Ltd.

103. McFadden E, *Management of Data in Clinical Trials* 1998; New York: John Wiley & Sons Ltd.

104. Spilker B *Guide to Clinical Trials* 1991; New York: Raven Press.

105. Taylor DM, McNeil JJ *A Guide to Good Research Practice,* Monash University; 2003.

106. Tombaugh TN. Test-retest reliable coefficients and 5-year change scores for the MMSE and 3MS. *Arch Clin Neuropsychol* 2005;20(4):485-503.

107. Bland RC, Newman SC. Mild dementia or cognitive impairment: the Modified Mini-Mental State examination (3MS) as a screen for dementia. *Can J Psychiatry* 2001;46(6):506-510.

108. Shumaker SA, et al. Estrogen plus progestin and the incidence of dementia and mild cognitive impairment in postmenopausal women: The Women's Health Initiative Memory Study: a randomized controlled trial. *J Am Med Assoc* 2003;289(20):2651-2662.

109. *Diagnostic and statistical manual of mental disorders.* 4th ed. 1994, Washington, DC: American Psychiatric Association.

110. Rosen WG, et al. A new rating scale for Alzheimer's disease. *Am J Psych* 1984;141(11):1356-1364.

111. Graham DP, et al. The Alzheimer's Disease Assessment Scale - Cognitive Subscale: Normative Data for Older Adult Controls. *Alzheim Dis Assoc Dis* 2004;18(4):236-240.

112. Doraiswamy PM, et al. Memory, language, and praxis in Alzheimer's disease: norms for outpatient clinical trial populations*. Psychopharmacol Bull* 1997;33(1):123-128.

113. Reid W, et al. Age at onset and pattern of neuropsychological impairment in mild early-stage Alzheimer disease. A study of a community-based population. *Arch Neurol* 1996;53(10):1056-1061.

114. Maj M, et al. *Evaluation of two new neuropsychological tests designed to minimize cultural bias in the assessment of HIV-1 seropositive persons: A WHO study. Arch Clin Neuropsych* 1993;8(2):123-135.

115. D'Elia, L., *Color Trails Test: professional manual*. 1996, Odessa, FL: Psychological Assessment Resources.

116. Inouye SK, et al. Clarifying confusion: the confusion assessment method. A new method for detection of delirium. *Ann Int Med* 1990;113(12):941-948.

117. Galasko D, et al. ADCS Prevention Instrument Project: Assessment of Instrumental Activities of Daily Living for Community-dwelling Elderly Individuals in Dementia Prevention Clinical Trials. *Alzheim Dis Assoc Dis* 2006;20(4 Suppl 3):S152-S169.

118. Ettinger WHJ, et al. Self-reported causes of physical disability in older people: the Cardiovascular Health Study. CHS Collaborative Research Group. *J Am Geriatr Soc* 1994;42(10):1035-1044.

119. Katula J, et al. Lifestyle Interventions and Independence for Elders pilot study: recruitment and baseline characteristics. *J Am Geriatr Soc* 2007;55(5):674-683.

120. Stewart A, et al. Physical activity outcomes of CHAMPS II: a physical activity promotion program. *J Gerontol A Biol Sci Med Sci* 2001;56(8):465-470.

121. Mangani I, et al. Physical exercise and comorbidity. Results from the Fitness and Arthritis in Seniors Trial (FAST). *Aging Clin Exp Res* 2006;18(5):374-380.

122. Pitkala KH, et al. Secular trends in self-reported functioning, need for assistance and attitudes towards life: 10-year differences of three older cohorts. *J Am Geriatr Soc* 2001;49(5):596-600.

123. Davin B, et al. [Demographic and socioeconomic factors associated with needs for home assistance among community-dwelling elderly: a study from the French Home Survey Handicaps-Disabilities-Dependence]. *Revue d Epidemiologie et de Sante Publique* 2005;53(5):509-524.

124. Alexander N, et al. Self-reported walking ability predicts functional mobility performance in frail older adults. *J Am Geriatr Soc* 2000;48(11):1408-1413.

125. Penninx BW, et al. Inflammatory markers and incident mobility limitation in the elderly. *J Am Geriatr Soc*, 2004;52:1105-1113.

126. Visser M, et al. Muscle mass, muscle strength, and muscle fat infiltration as predictors of incident mobility limitations in well-functioning older persons. *J Gerontol A Biol Sci Med Sci* 2005; 60:324-333.

127. Antman E, et al. Myocardial infarction redefined--a consensus document of The Joint European Society of Cardiology/American College of Cardiology committee for the redefinition of myocardial infarction. *J Am Coll Cardiol* 2000;36(3):959-969.

128. WHO. Stroke--1989. Recommendations on stroke prevention, diagnosis, and therapy. Report of the WHO Task Force on Stroke and other Cerebrovascular Disorders. *Stroke*

1989;20(10):1407-1431.

129. DeKosky ST, et al. The Ginkgo Evaluation of Memory (GEM) study: design and baseline data of a randomized trial of Ginkgo biloba extract in prevention of dementia. *Contemp Clin Trials* 2006;27(3):238-253.

130. Nahin RL, et al. Use of herbal medicine and other dietary supplements in community-dwelling older people: Baseline data from the Ginkgo Evaluation of Memory Study. *J Am Geriatr Soc* 2006;54(11):1634-1808.

131. Fitzpatrick A, et al. Recruitment of the elderly into a pharmacologic prevention trial: The Ginkgo Evaluation of Memory Study experience. *Contemp Clin Trials* 2006;27(6):541-553.

132. Rodriguez GJ, et al. Neuroimaging in stroke and seizure as neurological emergencies (NISSAN) study. *J Neuroimaging* 2008;18(1):9-14.

133. Narayan K. *personal communication.* 2008.

134. Adams HP, Jr., Bendixen BH, Kappelle LJ, Biller J, Love BB, Gordon DL, et al. Classification of subtype of acute ischemic stroke. Definitions for use in a multicenter clinical trial. TOAST. Trial of Org 10172 in Acute Stroke Treatment. *Stroke* 1993;24(1):35-41.

135. Kothari RU, et al. The ABCs of Measuring Intracerebral Hemorrhage Volumes. *Stroke* 1996;27(8):1304-1305.

136. Kozora E and Cullum CM. Generative naming in normal aging: Total output and qualitative changes using phonemic and semantic constraints. *Clin Neuropsychol* 1995;9(4):313-320.

137. Cargin JW, et al. Decline in verbal memory in non-demented older adults. *J Clin Exp Neuropsych* 2007;29(7):706-718.

138. Steffens DC, et al. Cerebrovascular Disease and Evolution of Depressive Symptoms in the Cardiovascular Health Study. *Stroke* 2002; 33(6):1636-1644.

139. Wilson RS, et al. Change in depressive symptoms during the prodromal phase of Alzheimer Disease. *Arch Gen Psych* 2008;65(4):439-446.

140. Sachdev P. Use of medications with anticholinergic properties and cognitive function in a young-old community sample. *Int J Geriatr Psychiatry* 2009;24: 578-84.

141. Tombaugh TN, et al. Normative Data Stratified by Age and Education for Two Measures of Verbal Fluency: FAS and Animal Naming. *Arch Clin Neuropsych* 1999;14(2):167-177.95.

142. Ruff RM, et al. Benton controlled oral word association test: Reliability and updated norms. *Arch Clin Neuropsych* 1996;11(4):329-338.

143. Fried L, et al. Reported preclinical disability identifies older women with early declines in performance and early disease. *J Clin Epidemiol* 2001;54(9):889-901.

144. Onder G, et al. Change in physical performance over time in older women: the Women's Health and Aging Study. *J Gerontol A Biol Sci Med Sci* 2002;57(5):M289-M293.

145. Tinetti M, et al. Shared risk factors for falls, incontinence and functional dependence. Unifying the approach to geriatric syndromes. *J Am Med Assoc* 1995;273:1348-1353.

146. Rantanen T, et al. Co-impairments as predictors of severe walking disability in older women. *J Am Geriatr Soc* 2001;49:21-27.

147. Fried L, et al. on behalf of the Cardiovascular Health Study Collaborative Research Group. Frailty in Older Adults. Evidence for a Phenotype. *J Gerontol A Biol Sci Med Sci* 2001;56:M146-M157.

148. Neuhaus JM, Kalbfleisch JD. Between-and within-cluster covariate effects in the analysis of clustered data. *Biometrics* 1998;54:638-645.

149. Vermeer SE, et al. Silent Brain Infarcts and the Risk of Dementia and Cognitive Decline. *N Engl J Med* 2003;348(13):1215-1222.

150. Baigent C. personal communication. 2007.

151. http://www.cancervic.org.au/downloads/cec/CanStats/50-Cancer-in-Victoria-2009.pdf

152. Thielke, et al. Prevalence, incidence, and persistence of major depressive symptoms in the Cardiovascular Health Study. *Aging & Mental Health* 2010;14:168-176.).

APPENDIX 1. ENDPOINTS AND OTHER MEASURES

Death from any cause or incident dementia or permanent physical disability.

These categories of disability are defined, respectively, as a) the assessment of dementia by DSM-IV criteria or b) the onset of 'a lot of difficulty' or 'inability' to perform any one of 6 Katz [75] ADLs.

1.1 All-cause mortality

Death certification and post mortem report.

1.2 Incidence of all-cause dementia

The trigger for a dementia assessment (assessment process is illustrated in Figure 1, Appendix 1) is a 3MS test score of ≤ 77, or a reliable minimum change score of 10 points or more from baseline on the last test will trigger referral for dementia assessment [76, 106, 107] with the cut-off being based on a regression model developed from the referenced community-based studies. This is necessary because as a predictor of dementia the change in an individual's 3MS is not linear across the range of scores (i.e. a change from 100 to 90 is not equal to a change from 90 to 80 or 80 to 70). Based on WHIMS data [108] participants who score below education-specific cut points of <88 (>8 yrs education) and <80 (<8 yrs education) provide positive prediction probability of >0.90 for dementia. Figures from the Edmonton community study of Bland and Newman [107], however, suggest a positive predictive value of about 0.33 for scores below 78. A clinical diagnosis of dementia noted in the medical records of any participant not already referred for dementia assessment will also trigger the same dementia assessment.

The diagnosis of incident dementia in ASPREE will be made on the basis of Diagnostic and Statistical Manual for Mental Disorders, American Psychiatric Association (DSM-IV) criteria [109], based on a standardized further assessment at least 6 weeks after the trigger event (tick-a-box DSM-IV assessment form). Diagnostic features include: memory impairment and at least one of the following: aphasia, apraxia, agnosia, disturbances in executive functioning. In addition, the cognitive impairments must be severe enough to cause impairment in social and occupational functioning. Importantly, the decline must represent a decline from a previously higher level of functioning. Finally, the diagnosis of dementia should NOT be made if the cognitive deficits occur exclusively during the course of a delirium. Classification of vascular dementia *versus* Alzheimer's disease or mixed dementia will not be requested, due to the lack of validated, clearly defined diagnostic criteria for vascular dementia. This triggered assessment will purposively utilize a different set of cognitive measures from those used in the routine visits to ascertain whether the cognitive aspects of the DSM-IV diagnosis of dementia are met:

- anterograde episodic memory impairment, language disturbance and ideational and constructional praxis will be assessed on the ADAS-cog [110] for which subscale normative (and AD) values are available [111, 112]
- visual agnosia on a Lurian figure-ground discrimination task normalized in a population of AD patients and elderly control subjects [113], and
- executive functioning on the Color Trails test [114] with normative data from the professional manual by D'Elia *et al*. [115].

Of these cognitive tests, all but the Color Trails are in the public domain. In addition to laboratory test results, delirium will be excluded on the Confusion Assessment Method proforma, short form [116] and by the deliberate minimum 6 week delay in the assessment after the trigger, which

should give time for unrecognized transient deliria to resolve. Medication records, with duration of use, will also be collected. Functional impairment will be assessed on the ADCS IADL scale [117], based on surrogate information. If the participant refuses the clinical assessment, staff are instructed to ask the participant to complete the 3MS, Hopkins and SDMT which will assist in providing valuable data in determining the endpoint. All cases will be then reviewed by one U.S. and one Australian member of a five member expert endpoint adjudication committee using DSM-IV criteria based on the uniformly applied tests, to determine whether the dementia endpoint has been reached. Any disagreement will trigger a consensus conference amongst the 5-member endpoint adjudication committee.

As is the case for the routine cognitive function measures for all ASPREE participants, study staff will be trained to administer the additional tests for dementia assessment. Where necessary, a surrogate will be asked to accompany the participant to complete certain scales (for IADL and delirium). Otherwise, this collateral information will be gathered by telephone interview from the most appropriate surrogate (e.g. partner, adult offspring, family physician). A number of home visits have been budgeted for in the cases where this is necessary to complete the testing.

In Australia, the physician performing the dementia assessment will usually be their general practitioner, although a neurologist, geriatrician or psychiatrist could also perform the assessment. In the U.S., the dementia assessment will be performed by a designated neurologist, geriatrician or psychiatrist located convenient to the ASPREE clinical site. The ASPREE site PI will send a letter (template) to the specialist informing them of the patient's abnormal cognitive test results and most recent laboratory test results, requesting that they follow-up with a dementia assessment.

The physician performing the dementia assessment will also complete a tick-box dementia evaluation form to classify the severity of the subject's cognitive impairment. The clinical summary or consultation letter of the dementia evaluation and the tick-box form will be returned to the site PI office. These results will be recorded in the web-based database and filed in the participant's file.

In both countries, the laboratory tests and essential CTs or MRIs will usually be covered by Medicare because they will be ordered as part of a dementia assessment. We have, however, also included costs to cover these laboratory tests and scans for those patients in the U.S. whose total costs are not covered by Medicare (such as those with co-pays, or those in the U.S. Medicare Advantage HMOs).

The laboratory tests anticipated as part of a standard US Medicare dementia assessment include the following: TSH (thyroid stimulating hormone), Vitamin B12, RBC (red blood cell), Folate, Electrolyte panel (includes sodium, potassium, chloride, CO_2, BUN [blood urea nitrogen] and creatinine), calcium, phosphate, glucose and CBC.

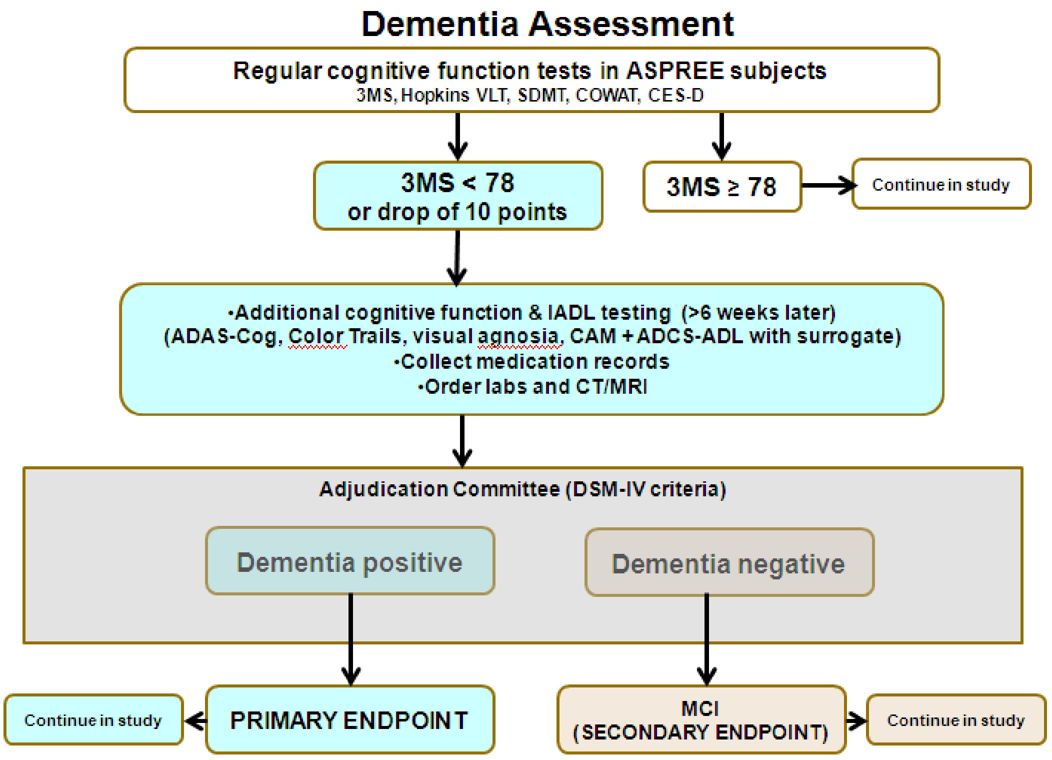

Appendix 1. Figure 1.
Flowchart for dementia assessment to reach a primary endpoint.

1.3 Loss of physical ability for activities of daily living

We will define activities of daily living disability on the basis of inability or severe difficulty with performance of one or more of the six Katz ADLs [75]. The instrument will be administered by interview at baseline and every six months. This outcome definition of disability has a long tradition in the geriatric literature, and is salient to both the physician community and the public. The rate of ADL disability increases with age, but it not often reported for age, sex and race strata. In a sub-group of the Cardiovascular Health Study participants matched for age and inclusion criteria (Newman A., personal communication, 8/2008) the rate of ADL disability was about 2% per year, higher in women than men.

The Katz ADLs include walking, bathing, dressing, transferring from a bed or chair, using the toilet, and eating. For ASPREE, we will use questions adapted from Katz for the Established Populations for the Epidemiologic Study of the Elderly (EPESE). The original Katz items [37] were classified by a health professional based on observation. These were adapted for self-report in the EPESE and are used in clinical trials and observational studies. These ADL items can be used alone but have also often been combined with items addressing IADLs and higher level mobility such as in the EPESE, CHS [118], LIFE [81] and other studies [119, 120, 121]. The questionnaire queries perceived difficulty in activities of daily living during the previous month. The following response options will be used, as were adapted from EPESE for the CHS study: (1) no difficulty, (2) a little difficulty, (3) some difficulty, (4) a lot of difficulty, or (5) unable to perform [118]. Use of these same items allows comparability of assessments for rates comparisons for many NIA-supported studies over many years.

We will also ask about “need for assistance” but will not define the outcome as dependency (‘need for assistance’) because this aspect of disability has been shown to be determined in part by available assistance and socioeconomic status [122, 123], while reported difficulty is more directly related to actual performance [124]. Self-report of ‘inability’ is rare in healthier older adults, such as would be selected for the ASPREE study, while severe difficulty is more commonly endorsed. Rates of this combined degree of difficulty were about 2% per year in the CHS study for a sub-cohort matched for age and other inclusion criteria to the ASPREE sub-cohort. Rates of ADL inability alone in this simulation were <0.5% per year, thus too infrequent an outcome for clinical trial. Therefore, for the primary endpoint ascertainment, a loss of function will be deemed to be a report of ‘a lot of difficulty’ or ‘unable to perform’ the ADL by self-report. For participants who are unable to answer due to illness, a proxy will be asked to answer these same items on behalf of the participant. For participants who miss a 6 month interview before death, a proxy decedent interview will be conducted to address the time frame of the onset of disability. This method has allowed classification of disability before death in the Health ABC study [125]. If all information is missing, the date of death will be used in the primary outcome as the first event and for disability analyses follow-up will be censored at last contact.

We will evaluate persistence of ADL disability as a primary endpoint. This will be done by reassessing level of disability using the same items at a subsequent 6 month telephone call or annual visit after the initial report. If the subsequent contact is missing, a proxy will be contacted for this information. If death precedes this follow-up contact, the death will be counted as the endpoint. If the subsequent contact is missed, but followed by death, persistent disability will be presumed. This method has worked well in determining persistent disability in the Health ABC study. [38, 125, 126]

2. Secondary Endpoints:

1. All-cause mortality
2. Fatal and non-fatal cardiovascular events including a) coronary heart disease death, b) non-fatal myocardial infarction, c) fatal and non-fatal stroke and d) hospitalization for heart failure.
3. Fatal and non-fatal cancer, excluding non-melanoma skin cancer
4. Dementia
5. Mild cognitive impairment (MCI)
6. Physical ADL disability
7. Depression
8. Major hemorrhagic events

2.1 All-cause mortality

As described above in 1.1

2.2 Fatal and non-fatal cardiovascular events including a) Coronary heart disease death, b) non-fatal myocardial infarction, c) fatal and non-fatal stroke and d) hospitalization for heart failure.

a) Coronary heart disease death - Myocardial infarction, sudden cardiac death, rapid cardiac death (death after possible myocardial infarction), cardiac failure death (with coronary cause) and other coronary death.

- *Myocardial infarction* - Autopsy or death certificate diagnosis, with definitive or suspected diagnosis of myocardial infarction within 4 weeks of death.
- *Sudden cardiac death* - Death occurring within one hour of the onset of new cardiac symptoms (ischemic chest symptoms or sudden collapse) or unwitnessed death after last

being seen without new cardiac symptoms, and in each case, without any coronary disease (clinically or at autopsy) that could have been rapidly fatal.

- *Rapid cardiac death (death after possible myocardial infarction)* - Death within 1-24 hours of the onset of severe cardiac symptoms unrelated to other known causes. Death in hospital with possible myocardial infarction (i.e. participants who have had typical ischemic pain and whose ECG and enzyme results fulfill the criteria for definitive MI and in whom there is no good evidence for another diagnosis for the event).
- *Cardiac failure (with coronary cause)* - Death due to heart failure (prior NYHA Class III-IV dyspnea), without any defined non-coronary cause.
- *Other coronary death* - Any death where the underlying cause is certified as coronary (and where there is no evidence of non-coronary cause of death, clinically or at autopsy).

b) Non-fatal myocardial infarction - (American College of Cardiology & European Society of Cardiology definition) [127]

Criteria for acute, evolving or recent MI. Either one of the following criteria satisfies the diagnosis for an acute, evolving or recent MI:

(1) Typical rise in troponin or CK-MB as biochemical markers of myocardial necrosis with at least one of the following:
   - ischemic symptoms;
   - development of pathologic Q waves on the ECG;
   - ECG changes indicative of ischemia (ST segment elevation or depression) OR coronary artery intervention (e.g. coronary angioplasty).

(2) Pathologic findings of an acute MI.

Criteria for established MI. Any one of the following criteria satisfies the diagnosis for established MI:

- Development of new pathologic Q waves on serial ECGs. The patient may or may not remember previous symptoms. Biochemical markers of myocardial necrosis may have normalized, depending on the length of time that has passed since the infarct developed.
- Pathologic findings of a healed or healing MI.

c) Fatal and non-fatal stroke - Fatal stroke will be defined as any death due to the rapid onset of a new neurological deficit attributed to obstruction or rupture in the intra-cranial or extra-cranial cerebral arterial system. Stroke will be defined according to the World Health Organization (WHO) definition as ‘rapidly developing clinical signs of focal (or global) disturbance of cerebral function lasting more than 24 hours (unless interrupted by surgery or death) with no apparent cause other than of vascular origin' [128]. This definition excludes cases of primary cerebral tumor, cerebral metastasis, subdural hematoma, post seizure palsy, brain trauma, and transient ischemic attack.

In Australia and the U.S., virtually all symptomatic strokes occurring in non-institutionalized patients are investigated by imaging with CT and/or MRI scanning. A National Stroke Audit carried out on a sample of patients throughout Australia in 2007 (http://www.strokefoundation.com.au/images/stories/healthprofessionals/national%20stroke%20 audit%20clinical%20report%20acute%20services.pdf), including remote areas, found that 91% of all patients received a CT scan or MRI within the first 24 hours after stroke. Given that people in remote areas in Australia are likely to be those not receiving CT scans or MRI, we expect all Australian ASPREE stroke cases will have accompanying CT or MRI scans as part of their usual care. In the U.S., 78 - 98% of patients who present to the hospital with acute symptoms of stroke have brain imaging at or shortly after the time of presentation [129-133]. CT scans, MRI reports and

ECG reports of rhythm disturbances (e.g., atrial fibrillation) will be included for consideration in the endpoint determination.

Because hemorrhagic stroke is both an endpoint and an adverse event, sub-classification will be used for strokes. Ischemic stroke is defined as a stroke for which a CT scan performed within 28 days of the onset of symptoms showed an area of low attenuation or an abnormal appearance in the vascular territory that corresponded to the recent symptoms and signs; or MRI showed a clinically relevant area of increased signal on diffusion weighted imaging, a slight hypointensity with or without mass effect on T1-weighted images, a bright area of hyper intensity with or without mass effect on T2-weighted images, or evidence of recent infarction on diffusion weighted MRI imaging. Alternatively cerebral infarction may be confirmed by autopsy. The TOAST classification for subtype of acute ischemic stroke will be utilized [134], in which both clinical features and ancillary tests (laboratory, radiology, and ultrasonography) are used to categorize five subtypes:

1. large artery atherosclerosis (embolus/thrombosis)
2. cardio embolism (high risk/medium risk)
3. small-vessel occlusion (lacunae)
4. stroke of other determined etiology
5. stroke of undetermined etiology
    a. two or more causes identified
    b. negative evaluation
    c. incomplete evaluation

**Features**	**Subtype**			
	**Large-artery atherosclerosis**	**Cardio embolism**	**Small-artery occlusion (lacunae)**	**Other causes**
**Clinical**				
Cortical or cerebellar dysfunction	+	+	-	+/-
Lacunar syndrome	-	-	+	+/-
**Imaging**				
Cortical, cerebellar, brain stem, or subcortical infarct >1.5 cm	+	+	-	+/-
Brain stem, or subcortical infarct <1.5cm	-	-	+/-	+/-
**Tests**				
Stenosis of extracranial internal carotid artery	+	-	-	-
Cardiac source of emboli	-	+	-	-
Other abnormality on tests	-	-	-	+

Table 1. A summary of the classification for ischemic stroke. Features of Trial of Org 10172 in Acute Stroke Treatment (TOAST) classification of subtypes of ischemic stroke [134].

<u>*Hemorrhagic stroke*</u> - is defined as a stroke in which a CT scan demonstrates an area of hyperdensity within the brain parenchyma with or without extension into the ventricles or subarachnoid space or, for scans performed beyond 1 week, an area of attenuation with ring enhancement after injection of contrast; MRI showing an area of hypointensity or isointensity on T1-weighted images or an area of marked hypointensity on gradient echo and T2-weighted images, or by autopsy demonstrating the origin of the hemorrhage as the cerebral parenchyma. Rarer causes and sites of intracerebral hemorrhage such as underlying arteriovenous malformation and spinal cord hemorrhage will be documented. Distinction between ischemic and hemorrhagic stroke can only be made with appropriate imaging. As noted above, CT or

MRI scanning is now undertaken as part of the investigation of virtually all cases of stroke in both the U.S. and Australia. Results of such imaging will be sought routinely by the ASPREE investigators for presentation to the endpoint committee. To complement our use of the TOAST classification for thrombo-embolic stroke, we will quantify the extent of intracerebral hemorrhage by assessing hemorrhage site and volume by CT or MRI. Volume will assessed by utilizing the ABC/2 formula [135] with hemorrhage sites as lobar, basal ganglionic or brain stem.

d) Hospitalization due to cardiac failure - Hospital discharge diagnosis of cardiac failure will trigger an assessment by the endpoint committee. Hospitalization for heart failure is defined as an unplanned overnight stay, or longer, in a hospital environment (emergency room, observation unit or inpatient care) or similar facility. Heart failure is defined as a patient having typical symptoms (e.g., dyspnea, fatigue) that can occur at rest or on effort that is characterized by objective evidence of an underlying structural abnormality or cardiac dysfunction that impairs the ability of the ventricle to fill with or eject blood (particularly during exercise). The diagnosis of heart failure may be further strengthened by a beneficial clinical response to treatment(s) directed towards amelioration of symptoms associated with this condition.

2.3 Incidence of fatal and non-fatal cancer (excluding non-melanoma skin cancer)

Morbidity and mortality from all-incident cancers (excluding non-melanoma skin cancers) will constitute a secondary endpoint. ASPREE cancers require histopathological confirmation unless metastasis is present on imaging or there is strong clinical evidence of metastasis. Mandatory reporting of all new cancers to state-based tumour registries in Australia, along with multiple mechanisms which are in place to ensure this data is complete (including routine surveillance of hospital discharge records and death certificates) will ensure robust cancer incidence data. In Australian and U.S. aged populations colon cancer will be a predominant incident cancer, being the second-leading cause of cancer related death. Because a common presentation of colon cancer is with rectal bleeding it is possible that the use of aspirin may cause ascertainment bias. However, this has not been documented in any of the intervention studies to date. In particular in the Nurses' Health Study [54] the investigators controlled for use of screening endoscopy in all of the multivariate analyses. They also evaluated the influence of aspirin among women who did not report having a positive fecal occult blood test result or did not undergo screening endoscopy. Among such women, the influence of aspirin was not materially altered.

2.4 Dementia

Assessment as described above in 1.2 and in flow chart (Figure 1, Appendix 1).

2.5 Mild cognitive impairment

To assess the presence of Mild cognitive impairment (MCI) the routine visit battery of cognitive function tests (3MS, SDMT, Hopkins, COWAT) will be utilized. Figure 2, Appendix 1 illustrates the steps in assessment for MCI. Participants who have normal 3MS scores but whose Hopkins delayed recall and/or SDMT and/or COWAT test scores fall by more than 1 SD from their own baseline value will be provisionally categorized as having MCI. Normative data for the Hopkins will be obtained from the Hopkins VLT-R Manual [79], for the SDMT from the SDMT Manual (Western Psychological Services, 1982) [78] followed by conversion from scaled scores, and for the COWAT from Kozora and Cullum [136]. The advantages of using within-subject decline rather than a pre-specified cut-off of 1.5 SD below age-adjusted mean are that i) about 7% of the population function below 1.5 SD anyway, leading to a reduction in specificity, ii) decline is more indicative of likely (further) progression to dementia than a single cross-sectional low score, and iii) decline-based criteria can detect individuals with a progressive course before they fall below a defined cut-off of -1.5 SD (e.g. [137]). For sub-classification of MCI, a decline in Hopkins delayed recall would be indicative of MCI-a (amnestic), a decline in either or both of the other tests indicates MCI-non-a (non-amnestic) and a decline in Hopkins delayed recall plus either of the

other tests indicates MCI-mixed.

In addition, those individuals who are triggered by a drop in 3MS score of > 10 (adjusted for education level attained) or a score of <78 to undergo dementia assessment (see section 1.2), but who are not subsequently classified as having dementia based on DSM-IV criteria, will be provisionally categorized as having MCI. Even without a diagnosis of dementia, a decrease in 3MS score of ≥ 10 points is a measure of clinically significant cognitive decline [39, 40, 87]. Assignment to MCI subcategories in these participants will be made by the endpoint adjudication committee on the basis of results from the routine cognitive exam and the triggered assessment. If no individual tests (other than the 3MS) have declined sufficiently to enable sub-categorization, the default category will be MCI-mixed.

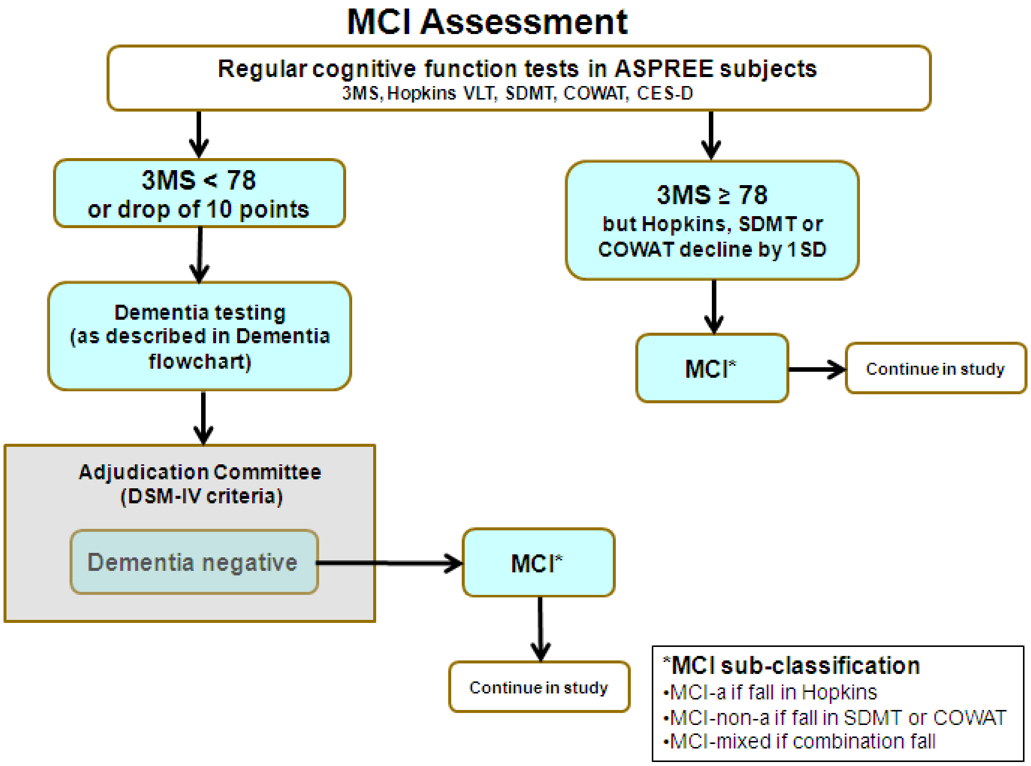

Appendix 1. Figure 2.
Flowchart for Mild Cognitive Impairment assessment to reach a secondary endpoint.
MCI-a (amnestic); MCI-non-a (non-amnestic)

In all cases, the provisional diagnosis of MCI will require confirmation of the decline (i.e. relevant result(s) still >1 SD below baseline, or 3MS still > 10 (adjusted) points below baseline, or still <78) at reassessment at 12 months. For those showing a decline indicative of MCI only at the last assessment, reassessment confirming the decline will be required 3 months later. (The 1 year timing allows reassessment to occur at the time of a scheduled routine visit, reducing assessment costs. The 3 month interval for those declining only at the final assessment avoids delaying analysis and publication unnecessarily.)

MCI, as traditionally defined by a deficit of -1.5 SD in a cognitive domain or domains in the presence of preserved general cognition (3MS >77), is not a study entry exclusion criterion. While those subjects ultimately destined to decline to dementia will be picked up at subsequent

assessments on the decline criteria used in ASPREE for MCI, a planned sub-group analysis will also be performed on the MCI category and sub-categories, after exclusion of any falling below -1.5 SD on the relevant measure on entry.

We wish to emphasize that MCI is a secondary endpoint and that one of ASPREE's strengths is that its test battery will allow several alternatives to identifying and defining MCI that may be useful for future large trials in this population.

2.6 Physical ADL disability

A secondary endpoint will be reached with a loss, confirmed at 6 months, of ability to perform one Katz ADL (as described above in section 1.3.).

2.7 Depression

A modified 10 questions version of the CES-D will be used. The modification omits questions related to lifestyle that are covered by the LIFE questionnaire. A score of 8 or higher on the 30 point CES-D 10 questions version is deemed clinically significant [138] and a secondary endpoint will have been reached.

Any such score will be communicated back to the family physician, and would typically result in a clinical assessment.

Anyone discharged from hospital with a principal diagnosis of depression will be deemed also to have reached a secondary endpoint for depression.

2.8 Major hemorrhagic events - *Clinically significant bleeding*

Gastrointestinal hemorrhages or hemorrhages at other sites that required transfusion, hospitalization, prolonged hospitalization, surgery, or are fatal will be considered as clinically significant bleeding events. Source information from clinical case notes and hospital medical records related to these events will be collected and sent to the ASPREE Data Management Center. A clinically significant bleed may lead to temporary or permanent trial medication cessation, according to the treating physician or surgeion's clinical judgment, but not exit from the trial. There is no standard accepted clinical trial definition of major GIT bleed in Australia. Our operating definition has been accepted by the Australian Gut Foundation.

3. Other Measures:

3.1 Cognitive function

In addition to the 3MS, the following depression and cognitive function tests will be administered at baseline and biennially, unless otherwise specified.

a) Depression - The CES-D 10 is administered in conjunction with the 3MS to ensure that a low score on the 3MS is not the result of depression. It will also be administered annually to ascertain new onset depression.

It is judged impractical to rely on clinical assessments of depression being sufficiently standardized across sites, countries and types of practice (family physicians *versus* psychiatrists) to enable substitution of clinical assessments for CES-D 10 scores.

In the context of a participant's 3MS score declining to the dementia assessment trigger range (as described in section 1.2), any participant also scoring 8 or more on the CES-D 10 will be referred to their usual physician for assessment and treatment, and will then be reassessed in ~3 months. If the CES-D 10 score has returned to below 8, or if the CES-D 10 was 8 or higher at baseline, but the 3MS score (adjusted according to Tombaugh [106]) is still in the trigger range, we will proceed with the additional dementia assessment as usual. If the

adjusted 3MS has returned to above the trigger, the participant continues in the study without going through the dementia assessment. If the CES-D 10 remains at or >8, their usual physician is advised for further management of the patient.

We will perform exploratory bivariate analyses to measure the association between performance on individual cognitive tests and depression. Depression in early Alzheimer's Disease has been found not to increase in severity, indicating that progressive cognitive changes sufficient to diagnose dementia are unlikely to be confounded by depression [139].

b) SDMT- An additional non-language based test of cognitive function, the Symbol-Digit Modalities Test (SDMT), is also included in cognitive function assessment as a measure of executive function, specifically of processing speed and activation, as well as of complex visual scanning [78]. The SDMT has been used longitudinally in other large-scale studies (e.g., [140]) and has been shown to be sensitive to both age-related cognitive decline and the increasing load of cerebrovascular disease. The SDMT does not use the alphabet. It is therefore not likely to penalize illiterate or semi-literate individuals, or those educated in languages not using the English alphabet. It is also easy to administer and score, and has both high 'ceiling' and low 'floor' effects, making it useful in assessment across a wide range of abilities.

c) Hopkins Verbal Learning Test- Revised (HVLT-R) - The HVLT-R [79] is a test of immediate and delayed recall, and delayed recognition. It has been widely used in previous studies of cognitive impairment. It is sensitive to change, has validated age and education-adjusted norms, and takes less time to administer than other word lists frequently used to test memory (such as the Rey Auditory Verbal Learning Test or the California Verbal Learning Test).

d) Controlled Oral Word Association Test (COWAT) - The COWAT [80] is a measure of executive function and verbal fluency. Patients are asked to generate as many words as possible beginning with the letter F. This is a shortened version of the full COWAT, which uses FAS, CFL, or PRW. Normative data for individual letters is available for this age group (e.g. [136]), and there has been shown to be high internal consistency between F, A and S, and C, F, and L, with coefficient alphas of 0.83 in each case. [141, 142] It should also be noted that a measure of verbal fluency by semantic category is included within the 3MS.

3.2 Physical Performance

Both performance-based and self-report instruments will be used to measure physical function. These detailed assessments will be carried out every other year, alternating with the cognitive assessment measures. Performance- based measures are very sensitive to change and can detect early decline prior to the onset of disability in activities of daily living (ADL). They have less of a ceiling effect than other measures of function or self-reported function and, as continuous variables provide more power to assess treatment effect. Small differences in performance (0.1 m/sec in gait speed) [87] are related to important subsequent mortality risk, so that prevention of decline would represent an important treatment effect of aspirin. Self report of increased difficulty with an activity identifies early declines in performance measures, and incident mobility disability. [124, 143] Limitations in or inability to perform ADL and mobility tasks reflect the subject's perception of their ability to perform in their own environment, and correlate moderately with performance- based measures [124], and therefore are considered to be complementary. Self- report instrumental activities of daily living (IADL) disability measures assess ability to carry out functions such as cooking, shopping and taking medication, which are also important for maintenance of independent living.

a) Performance based measures

Two performance-based measures will be used: grip strength and gait speed for usual

walking. These two measures are each strong independent predictors of mortality and disability, and very sensitive to change [87, 144]. Both can be assessed within 10 minutes and in both the home or clinic settings.

Hand grip strength will be measured in kilograms using a handheld isometric dynamometer (Jaymar; JLW Instruments, Chicago, Illinois). Grip strength of the dominant hand will be measured as the best of three trials. In the Women's Health and Aging Study, the mean baseline grip strength was 19.7 (± 5.9) kg, and declined by 1.4 (± 4.3) kg, or 6.8% over 3 yrs [138].

Gait speed will be assessed as the time in seconds to walk 3 meters (8 feet) at the participant's usual walking pace from a standing start. Time on the faster of two walks will be used to define scores [87]. Gait speed will be examined as a continuous variable. A walking speed lower than 0.42 m/s is considered to represent severe walking disability [145, 146]. Speeds of less than 1.0 m/sec are predictive of mortality and hospitalization [43] and changes of >0.1 m/sec related to significant perceived decline [42].

b) Self- report measures (using ADL and IADL Questionnaires)

Mobility activities – walking one block, walking a quarter of a mile(about 2 or 3 blocks), walking one mile, climbing one flight of stairs, walking in the grocery aisle without sitting or leaning on the cart, getting in and out of a car.

Instrumental ADLs - doing light housework, preparing own meals, managing own money, using the telephone, doing errands, taking care of a family member, visiting relatives or friends, participating in community activities, taking own medications.

Upper extremity activities (tertiary) - lifting heavy groceries or 10 pounds, raising arms overhead, lifting heavy objects, gripping with hands.

c) Frailty measures

These will include a) measures of gait speed and grip strength (as measured in performance measures above) to assess weakness, b) a single item question (from the SF-12 below) on fatigue/energy level, c) annual clinic assessment of weight to assess weight loss, and d) a single item regarding physical activity, adapted from the LIFE study. These few items can be combined to create an assessment of frailty using the Fried criteria [147]. This will be assessed every other year in conjunction with the performance measures.

3.3 Maintaining quality of life

Quality of life will be assessed annually using the Short Form 12 (SF-12) item questionnaire, which includes physical functioning, mental health, role functioning (physical and emotional), health perceptions in the last year, energy level and pain level [82]. This will provide a basic assessment of functioning on a more frequent basis than the more detailed physical function assessments which, as stated above, will be conducted every other year.

3.4 Hemoglobin

Measurement of hemoglobin levels will be undertaken at baseline and annually.

3.5 Hospitalization

Total hospitalizations and hospitalization for reasons other than endpoints will be collected. Hospitalization is defined as an official admission that is for a duration greater than 24 hours or a minimum of 2 calendar days where exact time of stay is unavailable.

3.6 Urine albumin:creatinine ratio

Measurement of urinary albumin:creatinine ratio will be undertaken at baseline and at designated annual visits. A ratio higher than the normal value for gender (males: 2.5mg/mmol;

females: 3.5mg/mmol) will be reported to the participant’s usual treating doctor for follow up.

APPENDIX 2. SAMPLE SIZE & POWER CALCULATIONS

For the primary endpoint, a sample size can be calculated that gives the number of primary endpoint events required for ASPREE to have 90% power for their subsequent analysis by a univariate Cox proportional hazards regression model. To estimate the number of participants required to yield these events, assumptions are made for (i) the event rates that will be seen in ASPREE participants and (ii) the duration of participant follow-up during the study. Having obtained a sample size for the primary endpoint we examine the power or effect size that this number of participants gives us for each secondary endpoint. Hence, assumptions are also required for the event rates of secondary endpoints. In general, Australian event rates have been used as a basis for ASPREE participants. This is justified on the grounds that approximately two-thirds of participants will be recruited in Australia and using the lower Australian rates errs on the conservative side for sample size calculations. For example, Australian rates of all-cause mortality, cardiovascular events and cancer are lower than in the U.S. (see Table 2 of the Research Plan). For the primary outcome, population-specific rates are used where there is strong evidence that a minority group is at increased risk.

The study will have a 1:1 aspirin:placebo group allocation ratio.

All power and sample size calculations were performed with a two-sided alpha error rate of 0.05 for a univariate Cox proportional hazards regression analysis of time to first event using the 'stpower cox' command in Stata (Stata Statistical Software, Release 10, StataCorp, College Station, TX, 2007).

Assumptions used for the original sample size and power calculations include:

- Participants will be 50%, 30%, 15%, 5% in age groups 70-74 years, 75-79 years, 80-84 years, 85 years and over, respectively.
- Participants will be 45% male and 55% female.
- Based on experience with ANBP2, the effect of clustering due to recruitment by country and enrolling general physician or clinical research site will be minimal and no explicit adjustment to the number of participants needs to be made. In any case, since randomization is at the individual participant level, treatment effect is a within-cluster comparison and this protects against any concern over loss of power due to clustering [148].
- Accrual of participants is over approximately a 2 year period with an average of 5 years of follow-up per participant.
- Rates are assumed for participants based on their age at entry to the study. Further, the rates are assumed to stay constant throughout participants' follow-up.

An amendment to the recruitment strategy in the US in 2011 changed the configuration of the expected participant profile. Specifically the split of recruited participants among the four categories Australian, White US, Hispanic, African American was expected to change to 84.2%, 7.9%, 2.63%, 5.26% respectively. The age distribution in the US participants was expected to change to 20%, 47%, 19%, 8%, 6% in the age bands 65-69 years, 70-74 years, 75-79 years, 80-84 years, 85 years and over, respectively.

PRIMARY OUTCOME

We have designed ASPREE to have 90% power to detect an effect of aspirin as described by a hazard ratio of 0.90 in an intention-to-treat analysis. Aspirin has dual mechanisms of action likely to affect the primary endpoint. Antiprostaglandin effects are postulated to affect atherogenesis and carcinogenesis and acetylation within platelets is likely to affect atherothrombotic and hemorrhagic events [5]. We would propose that the latter mode of action is likely to have a dominant effect on the primary endpoint due to lag time in the former [54] and

vascular events being the major determinant of morbidity (including dementia [5, 149] and mortality in the aged [54]. The Anti-thrombotic Trialists Collaboration has provided us with a meta-analysis of all six primary prevention trials by age categories which shows that the point estimate of the benefit of aspirin over no aspirin treatment on all-cause mortality alone is 0.95 with wide confidence intervals (95% CI: 0.70 – 1.14) in those subjects aged 70 years and over [150]. The point estimate of aspirin's effect on adverse cardiovascular disease events from the same meta-analysis is 13% (95% CI: 0.65 – 1.16). This suggests that a 10% treatment effect for aspirin is plausible. These studies experienced cross-over of participants from one treatment group to the other for reasons which include the development of a non-fatal, non-disabling cardiovascular or cerebrovascular event necessitating aspirin therapy. Similarly, we expect 5% per annum of placebo-group participants to initiate aspirin use or *vice versa*.

The following assumptions have been made for event rates for the primary endpoint of death from any cause or incident dementia or physical disability (the latter two events defined respectively as an assessment of dementia by DSM-IV criteria and loss of any one of 6 Katz ADLs).

i. Dementia annual incidence rates will be 3 per 1000 for the 65-69y age group (US minorities only), 6/1000 (70-74y), 11/1000 (75-79y), 20/1000 (80-84y), 37/1000 (85y+) [98, 99] for Australian, U.S. Hispanic and U.S. White (non-Hispanic) participants. For U.S. African American the rates are doubled [99, 100].

ii. Loss of a Katz ADL; annual incidence rate for males = 19.6/1000, females = 26.5/1000 (these incidence rates are from the rates observed in the subset of ASPREE-like participants in the CHS (Newman, A. personal communication, August, 2008) which showed similar rates in U.S. Whites and U.S. minorities). To allow for 20% of the US minority participants being in the 65-69y age group the rates for this population are weighted away from the above rates and towards rates of 13.3 in males and 14.8 in females for this age group. This results in an expected rate of US minorities in the placebo group of 21.5 per 1000 person-years.

iii. Age specific annual mortality rates are from the 2004 Australian population census (source=ICD10 ALL, Australia, 1907–2004 - General Record of Incidence of Mortality, GRIM; http://www.aihw.gov.au/mortality/data) apply for the Australian and U.S. White (non-Hispanic) participants. For males these were 2666.1/100000 for the 70-74y age group, 4484.1/100000 (75-79y), 7643.3/100000 (80-84y), 15635.9/100000 (85y+), and for females the corresponding rates were 1467.6/100000 (70-74y), 2722.9/100000 (75-79y), 5107.5/100000 (80-84y), 12892.7/100000 (85y+).

iv. Age-specific mortality rates in U.S. African American and Hispanic participants are based on 2005 rates specific to each minority (U.S. CDC statistics, 2005; http://www.cdc.gov/nchs). For male African Americans these were 3227.81/100000 for the 65-69y age group, 4542.75/100000 (70-74y), 6393.35/100000 (75-79y), 8997.85/100000 (80-84y), 13809.8/100000 (85y+), and for female African Americans the corresponding rates were 1963.61/100000 (65-69y), 2901.85/100000 (70-74y), 4288.4/100000 (75-79y), 6337.46/100000 (80-84y), 12789.9/100000 (85y+). For male Hispanics these were 2541.17/100000 for the 70-74y age group, 3899.60/100000 (75-79y), 5984.21/100000 (80-84y), 10140.5/100000 (85y+), and for female Hispanics the corresponding rates were 1639.83/100000 (70-74y), 2649.49/100000 (75-79y), 4280.79/100000 (80-84y), 9068.4/100000 (85y+).

v. Participants will be healthy relative to their same-aged population counterparts and that mortality rates will be 50% lower than the general population rates given in the two assumptions immediately above

vi. To allow for the analysis to be time to first event, individual rates for death, ADL deterioration, and dementia can be summed and the sum reduced by 10% to allow for the potential for different events to occur in the same person [40].

vii. Using the assumptions above, the overall primary endpoint rate in the Australian and U.S. White participants will be 45.4 per 1000 person years, and the event rate in the U.S. minority groups will be 48.9 per 1000 person years.

The study aims for 5 years average follow-up per participant and for the primary endpoint this 'at risk' time for occurrence of a first primary endpoint event will be reduced to an average of 4.25 years per participant. The reasons for this reduction include censoring due to the occurrence of a primary endpoint, loss to follow-up for death (which is expected to be extremely low due to access to mortality statistics through National Death Index records in Australia and U.S), non-completion of dementia screen or diagnosis, and non-completion of ADL's. In total, we assume that 5% of participants per year will have an occurrence of the primary endpoint or have insufficient follow-up to enable assessment of their primary endpoint status.

On the basis of these assumptions, 311 primary endpoint events in 1500 mainly U.S. minority participants and 3374 primary endpoint events in 17,500 Australian and U.S. White participants will yield 89% power (two-sided alpha error rate of 0.05) to detect a 10% reduction (risk ratio of 0.90) in the primary endpoint with aspirin. The final recruitment numbers that were achieved are approximately 16,500 participants in Australia and 2,500 participants in the U.S. Note that this is a variation from the original protocol which specified a plan to recruit 12,500 and 6,500 participants (4,500 from minority populations all aged 70+y) in Australia and the US respectively which had a 90% power.

The sample size for the primary endpoint of death from any cause or incident dementia or physical disability will be 19,000 participants.

SECONDARY OUTCOMES

1. All-cause mortality

Anticipated rates of all-cause mortality are given above under the primary endpoint sample size calculation. A sample size of 19,000 provides 90% power to detect a hazard ratio of 0.842 comparing the aspirin group with placebo in an intention-to-treat analysis (all other assumptions as for primary endpoint).

2. Fatal and non-fatal cardiovascular events including a) coronary heart disease death, b) non-fatal myocardial infarction, c) fatal and non-fatal stroke and d) hospitalization for heart failure

- Based on the experience in other primary prevention trials in the elderly [150] it is assumed that the rate of CVD events, (including hemorrhagic stroke; see below) will be 17.0 per 1000 person-years (70-74y age group), 25.0 per 1000 person-years (75-79y), 38.0 per 1000 person-years (80y+).
- These event rates lead to an overall annual rate of 23.6/1000 from which an annual rate of 1.2/1000 for hemorrhagic stroke [70] is subtracted. Hence we expect this secondary endpoint to have an annual rate of 22.4/1000 in the placebo group.
- Assume that there will be an annual loss to follow-up of 3% of participants per annum for this endpoint due to other causes of mortality and, based on ANBP2, an expected very

low rate of non-assessment of non-fatal CVD status. This corresponds to an average of 4.7 years per participant at-risk time.

A sample size of 19,000 participants gives 90% power to detect a 14.2% reduction in CVD event risk (hazard ratio 0.858) in an intention-to-treat analysis based on the above assumptions.

3. Fatal and non-fatal cancer, excluding non-melanoma skin cancer

For a sample size of 19,000 subjects to 3% per annum drop out, the study will have 90% power to detect the following hazard ratios for the comparison of incidence rates between intervention and placebo in an intention to treat analysis (assuming rates in the placebo group as described in the table, and other assumptions as for primary endpoint);

	Rates with placebo	Hazard ratio
Colorectal cancer	3.09 per 1000 person years [151]	0.64
Prostate cancer	3.04 per 1000 person years [151]	0.64
Lung cancer	2.52 per 1000 person years [151]	0.61
Breast cancer	1.95 per 1000 person years [151]	0.57
Melanoma	1.47 per 1000 person years [151]	0.55
All cancer	21.35 per 1000 person years [151]	0.86

The major cancer endpoint will be the incidence (and mortality) of 'All cancers'. The effect of aspirin on colorectal, prostate, lung and breast cancer, and melanoma will be assessed individually as they are the most common cancer types in the population. It is anticipated that with further follow up above and beyond the 5 years currently planned for ASPREE, there would be sufficient power to detect more subtle aspirin effects.

The 'lethality' of cancers occurring in ASPREE will also be addressed. Cancer recurrence, in particular the development of metastatic disease, will be collected in order to see if aspirin may influence the metastatic potential of incident cancers.

4. Dementia

Anticipated rates of dementia are given above under the primary endpoint sample size calculation. A sample size of 19,000 provides 90% power to detect a hazard ratio of 0.807 comparing the aspirin group with placebo in an intention-to-treat analysis (all other assumptions as for primary endpoint).

5. Mild cognitive impairment

Our primary interest is the difference between placebo and aspirin groups in average 3MS rate-of-change from baseline to the end of 5 years of follow-up. Because this measurement is on a continuum, only 5200 participants are required to provide 90% power to detect a clinically relevant 1 point smaller decline over 5 years in the mean 3MS score of the aspirin group than the placebo group. Even assuming the worst-case scenario, for variability in 3MS measurements (for example SD=10) and the strength of correlation between baseline and end-of-study measurements, the sample size of 19,000 participants will be more than sufficient to have 90% power to detect a 1-point smaller decline in this secondary endpoint.

6. Physical disability

Anticipated rates of deterioration in physical ability to perform each Katz ADL are given above

under the primary endpoint sample size calculation. A sample size of 19,000 provides 90% power to detect a hazard ratio of 0.857 comparing the aspirin group with placebo in an intention-to-treat analysis (all other assumptions as for primary endpoint).

7. Depression

We anticipate incidence of new depression of 5% per year based on the estimated probability of healthy older males aged 65-75 years developing major depression in one year.[152] This estimate is globally conservative due to i) evidence that females develop depression at higher rates than males [152] (the ASPREE population is likely to be 45% male) ii) those aged over 75 have increased probability of depression [152], iii) incidence of major depression (CES-D >10 points) is lower than pre-morbid depression (CES-D score cut off of 8 points), and iv) the ASPREE study will average 4.25 years of follow up with points (ii-iii) increasing with time. With a sample size of 19,000 followed for an average of 4.25 years with a 3% per annum drop out due to deaths and other primary events, ASPREE has 90% power to detect a hazard ratio of 0.799 comparing the aspirin group with placebo.

8. Major hemorrhagic events

We anticipate rates of major bleeds of 11 events per 1000 person-years in the placebo group based on:

- Hemorrhagic stroke rate of 1.2 per 1000 person years [70].
- Serious upper GI complications: between 2 and 14 events per 1000 person-years for those over 70 years, increasing sharply with age [5, 70].

Given a total sample size of 19,000, we have 90% power to detect a hazard ratio of 1.23 comparing the aspirin group with placebo in an intention-to-treat analysis where compliance with medication and drop-out are assumed as for the primary endpoint.

APPENDIX 3 CONFLICT OF INTEREST

General Principles

1. This full policy is to be made public on our Website and in publications when possible.

2. The primary concerns are twofold. First, that the ASPREE investigators maintain the internal integrity of the study by which we mean the confidence among ourselves (investigators and staff) as we develop and modify the detailed protocol, that advice is being given and decisions are being made in as unbiased and fully informed manner as possible. Second, that we maintain the external integrity of the study by which we mean the acceptance of our process and results as having met public standards of conduct.

3. To meet these goals we will obtain full disclosure by all of the key members of the study (as defined below in item 4) of their, and their immediate family's, financial relationships with all pharmaceutical and biomedical companies judged to have an active or potential interest in the conduct and outcome of the study. These are to be reported annually on a standard form, each of which will be reviewed on at least an annual basis or more frequently if there is a significant change from the last report, by an Oversight Committee. The Oversight Committee will be comprised of the Chair of the Steering Committee and the Chair of the International Operations Committee. The information to be reported will not include specific dollar amounts, although the definitions below require that certain relationships be segregated by those above and below certain dollar thresholds.

4. All of the study PIs, Co-PIs, and the Steering Committee and its various subcommittees' members are covered by this policy.

5. A conflict of interest will not necessarily exclude any member of the study from participating in study discussions, unless required in individual cases by the Oversight Committee. However, full disclosure of all potential conflicts of interest will be made at each meeting to all attendees in an effective, but non-cumbersome manner. This includes the full Steering Committee as well as each of its subcommittees.

6. A significant financial conflict of interest, defined below, will cause a person to excuse himself or herself from voting on all issues related to the conflict. All such actions will be recorded and kept as part of the study record by the Oversight Committee.

7. All financially relevant relationships are to be reported. Only those relationships that are between the individual and the specific company (rather than between the individual's parent institution and the specific company, for example) present the potential for a significant financial conflict of interest, defined under paragraphs 9a and 9b below. Specifically, research funding for contracts or grants to the parent institution which provide support to the individual, his/her laboratory or his/her close scientific collaborators is not ordinarily judged to present the potential for a financial conflict of interest, although such awards are to be fully disclosed as a part of this policy.

8. Those financially relevant relationships that are to be reported include employment, consultancies, board memberships, honoraria, stock ownership or options, grants, contracts, patents received or pending, and royalties. The Oversight Committee will decide, with #9 below as a guideline, whether any of these and other relationships in each individual case is significant enough to warrant excuse from voting or discussions.

9. A significant financial relationship is defined to exist:

a) when the dollar amount awarded on an annual basis, or expected-to-be awarded on an annual basis, with regard to each related corporate relationship exceeds $10,000. The Oversight Committee may also judge lower dollar amounts as significant in specific/individual circumstances.

b) when there is any equity holding in a related company (excluding mutual funds and blind trusts). Again the Oversight Committee may decide in individual circumstances that the equity holdings are relatively minor enough to not present a real conflict of interest.

c) Significant financial relationships in existence since September, 2009 between ASPREE investigators and all pharmaceutical and biomedical companies, judged to have an active or potential interest in the conduct and outcome of the study, will be described in all study reports and publications. In addition we will meet or exceed the reporting standards of the journals publishing our manuscripts.

APPENDIX 4. DSMB CHARTER

ASPirin in Reducing Events in the Elderly
1RO1AG029824-01A2
Richard Grimm, MD
John McNeil, MD
Minneapolis Medical Research Foundation

The Data and Safety Monitoring Board (DSMB) will act in an advisory capacity to the National Institute of Aging (NIA) Director to monitor participant safety, data quality and evaluate the progress of the study. Dr. Richard Grimm, The Berman Center for Outcomes and Clinical Research, Minneapolis, MN and Dr. John McNeil, Monash University, Melbourne, Australia are conducting the ASPirin in Reducing Events in the Elderly study funded by the National Institute on Aging.

DSMB Responsibilities

The DSMB responsibilities are to:

- review the research protocol, informed consent documents and plans for data safety and monitoring;
- advise the NIA on the readiness of the study staff to initiate recruitment;
- evaluate the progress of the trial, including periodic assessments of data quality and timeliness, recruitment, accrual and retention, participant risk versus benefit, performance of the trial sites, and other factors that can affect study outcome;
- consider factors external to the study when relevant information becomes available, such as scientific or therapeutic developments that may have an impact on the safety of the participants or the ethics of the trial;
- review study performance, make recommendations and assist in the resolution of problems reported by the Principal Investigators;
- protect the safety of the study participants;
- report to NIA on the safety and progress of the trial;
- make recommendations to the NIA, the Principal Investigators, and, if required, to the Food and Drug Administration (FDA) concerning continuation, termination or other modifications of the trial based on the observed beneficial or adverse effects of the treatment under study;
- if appropriate, review interim analyses in accordance with stopping rules, which are clearly defined in advance of data analysis and have the approval of the DSMB;
- ensure the confidentiality of the study data and the results of monitoring; and,
- assist the NIA by commenting on any problems with study conduct, enrollment, sample size and/or data collection.

The DSMB will discharge itself from its duties when the study is complete.

Membership

The DSMB will consist of 7 members and four will constitute a quorum. When the DSMB consists of more than five individuals, at least 50% of members must be present at the meeting to constitute the quorum. Members are usually recommended by Dr. Richard Grimm and Dr. John McNeil and /or NIA Program Official, and the NIA Director approves the composition of the DSMB and its membership. Membership consists of persons completely independent of the investigators who have no financial, scientific, or other conflict of interest with the trial. Collaborators or associates of Dr. Richard Grimm or Dr. John McNeil are not eligible to serve on the DSMB. Written documentation attesting to absence of conflict of interest is required.

Dr. JP Mohr, Columbia University has been selected by NIA in consultation with the Principal Investigators to serve as the Chairperson and is responsible for overseeing the meetings, developing the agenda in consultation with the NIA Program Official and the Principal Investigators. The Chair is the contact person for the DSMB. The Berman Center and Monash University shall provide the logistical management and support of the DSMB.

Board Process

At the first meeting the DSMB will discuss the protocol, suggested modifications, and establish guidelines to study monitoring by the Board. The DSMB Chairperson in consultation with the Principal Investigators and the NIA Program Official as needed, will prepare the agenda to address the review of study materials, modifications to the study protocol and informed consent document, initiation of the trial, appointment of a safety officer, as needed, reporting of adverse events, statistical analysis plan including interim analysis and stopping rules, etc.

Meetings of the DSMB will be held at least two times a year at the call of the Chairperson and / or NIA Program Official. The NIA Program Official or designee will be present at every meeting. An emergency meeting of the DSMB may be called at any time by the Chair or by the NIA should participant safety questions or other unanticipated problems arise.

Meetings shall be closed to the public because discussions may address confidential participant data. Meetings are attended by at least one of the Principal Investigators and members of his/her staff. Meetings may be convened as conference calls as well as in-person.

Meeting Format

DSMB meetings will consist of open and closed sessions. Discussion held in all sessions is confidential. At least one of the Principal Investigators and key members of the study team will attend the **open sessions.** Open session discussion will focus on the conduct and progress of the study, including participant accrual, protocol compliance, and problems encountered. Unblinded data are not presented in the open session.

The **closed session** will be attended by the DSMB members and the NIA representative(s). The study statistician may be present, at the request of the DSMB. Any data by blinded study group and, as necessary, unblinded data, are presented during the closed session. Either the DSMB or the NIA can request review of unblinded data in the closed session. Investigators will still remain blinded.

If necessary, an **executive session** will be attended by voting DSMB members and the NIA staff and their representatives. The executive session will be held to identify and discuss the

DSMB's recommendations to the NIA. The study staff may be present, at the request of the DSMB, during the executive session.

Each meeting must include a recommendation to continue or to terminate the study made by a formal DSMB majority or unanimous vote. Should the DSMB decide to issue a termination recommendation, the full vote of the DSMB is required. In the event of a split vote, majority vote will rule. The DSMB Chair provides the tiebreaking vote in the event of a 50-50 split vote.

A recommendation to terminate the study may be made by the DSMB at any time by majority vote. The Chair should provide such a recommendation to the NIA immediately by telephone and email. After the NIA Director makes a decision about whether to accept or decline the DSMB recommendation to terminate the study, the PI is immediately informed about his decision.

Meeting Materials

DSMB interim report templates will be prepared by the study staff, typically the statistician, to be reviewed by the DSMB members at the first meeting. Interim data reports generally consist of two parts:

- Part 1 - Open Session Report and
- Part 2 - Closed Session Report

Format and content of the reports for both the open and closed sessions and plans for interim analyses should be finalized and approved by the DSMB, although changes throughout the trial may be requested by the Board.

The reports will list and summarize safety data and describe the status of the study. All meeting materials should be sent to the DSMB and the NIA at least 10 to 14 days prior to the meeting. The reports are numbered and provided in sealed envelopes within an express mailing package or by secure email as the DSMB prefers.

1. **Part 1 - Open Session Reports:** Open session reports generally include administrative reports by site that describe participants screened, enrolled, completed, and discontinued, as well as baseline characteristics of the study population. Other general information on study status may also be presented. Listings of adverse events and serious adverse events as well as any other information requested by the DSMB may also be in the open session report, but none of the data should be presented in an unblinded manner. The DSMB may direct additions and other modifications to the reports on a one-time or continuing basis.
2. **Part 2 – Closed Session Report:** Closed session reports generally present the same information as presented in the open session but by blinded treatment group (e.g. A/B, etc.). The reports may also contain data on study outcomes, including safety data, and depending on the study, perhaps efficacy data. The Closed Session reports should be destroyed at the conclusion of the meeting. If the meetings are held by telephone, printed copies of the closed reports should be destroyed immediately following the meeting. If a study has an interim analysis, it is also discussed in the closed session.

Additional Reports

1. **Mailings to the DSMB:** On a scheduled basis (as agreed upon by the DSMB) blinded safety data could be communicated to all or select DSMB members.

2. **Access to Interim Data:** Access to the accumulating endpoint data should be limited to as small a group as possible, such as the statistician. Limiting the access to interim data to the DSMB members relieves the investigator of the burden of deciding whether it

is ethical to continue to randomize individuals and helps protect the study from bias in recruitment and/or evaluation.

3. Interim Analyses: In studies in which an interim analysis is planned, the DSMB will need to decide when the analysis will take place (e.g. after n participants have been accrued). The interim analysis will be reviewed in closed session and may be presented in blinded group A/B format, however the DSMB may request review of the unblinded data.

Interim analyses of efficacy data are performed only if they are planned and approved in advance and criteria for possible stopping are clearly defined.

Reports from the DSMB

A formal report containing the recommendations for continuation or modifications of the study will be prepared by the KAI Research, Inc. and reviewed and approved by DSMB Chairperson. The draft report will be sent to the DSMB members not later than four weeks after the meeting. Once approved by the DSMB members, the NIA Program Official will forward the formal DSMB recommendation to the Principal Investigators. It is the responsibility of the Principal Investigators to distribute the DSMB recommendation to all co-investigators and to ensure that copies are submitted to all the IRBs associated with the study.

As previously stated, the formal DSMB report must include a recommendation to continue or to terminate the study. This recommendation should be made by formal majority vote. A termination recommendation may be made by the DSMB at any time by majority vote. The NIA is responsible for notifying the Principal Investigators of a decision to terminate the study. The report should not include unblinded data or discussion of the unblinded data.

Confidentiality

All materials, discussions and proceedings of the DSMB are completely confidential. Members and other participants in DSMB meetings are expected to maintain confidentiality.

APPENDIX 5. ACES (ASPREE CANCER ENDPOINTS STUDY) TUMOR TISSUE BANKING

From 2013, the National Cancer Institute in the U.S. (one of the Institutes within the NIH) funded the ASPREE Cancer Endpoints Study (ACES) as a subsidiary of the main ASPREE study. ACES will allow for the exploration of DNA related molecular mechanisms of aspirin's protective effect against cancer and cancer-associated mortality and metastases using tumor tissue. A key component of this study is to establish a biologic specimen repository for tumor tissue from the ASPREE healthy ageing population in the U.S. and Australia for future use by ASPREE, NIA and NCI investigators, and academicians from the broader research community. Participants in the ASPREE clinical trial will be asked to agree to allow ASPREE to be provided with a small specimen of tumor tissue collected at the time of diagnosis or treatment in those diagnosed with cancer.

At a time in the future and under separate application, the stored tumor tissue will be analyzed to address specific questions regarding the association of biomarkers and major health outcomes. Future applications for ethical approval will be made regarding projects that address disease outcomes of interest observed during the 5 year period of the study. The tumor tissue collection in both countries would improve our ability to measure the longitudinal effects of aspirin on incident and recurrent cancer and metastases, and in turn, potentially lead to the development of preventive and therapeutic targets for these outcomes.

Participant Consent: At the time of the original consent to the ASPREE study, or at an annual visit if after June 2013, each ASPREE participant will be asked to consent to ASPREE investigators obtaining a small sample of tumor tissue collected in the process of future cancer diagnosis and surgical removal of tumor tissue. Consent will require the ticking of a box at the end of the consent form (see below). Wording to be included in the ASPREE PICF, page 4 of 6:

'Cancer Biopsy:

During the course of the study, some people may have biopsies taken for cancer diagnosis. We ask you for permission to access a small sample of this biopsy, if available. Please tick the box at the end of this consent form to indicate permission for this access.'

Tumor Tissue Processing, Shipping and Storage (Tumor Specimen Biorepository): ASPREE staff will follow the appropriate procedures for tissue procurement, as dictated by institution from which the tissue is to be sourced (hospital or registry). Procured tumor specimens will be sent to the ASPREE Centre / ASPREE Healthy Ageing Biobank, Monash University located at the Alfred Hospital for storage, with potential subsequent shipment to a US-Based Tissue Procurement Facility (pending future funding). Upon arrival at the ASPREE Healthy Ageing Biobank, the tumor tissue will be barcoded, and this code scanned and entered into the Laboratory Information Management System (LIMS) prior to storage. At the time of tumor tissue collection, all relevant information relating to the tissue (e.g. pathology reports, tissue handling and patient case reports) will be copied or scanned, and electronically entered into the ASPREE clinical trial database. A Tumor Tissue Biospecimen Information Sheet will be completed by the study staff member, identified by only the participant's ASPREE study number and acrostic, and the information contained therein entered into the ASPREE Healthy Ageing Biobank LIMS. All hard copy Tumor Tissue Biospecimen Information Sheets will be stored in a secure location at the ASPREE Healthy Ageing Biobank.
