# Supplementary material for: Low-Dose Aspirin and the Risk of Stroke and Intracerebral Bleeding in Healthy Older People: Secondary Analysis of a Randomized Clinical Trial
Source: JAMA Netw Open. 2023 Jul 26;6(7):e2325803. doi: 10.1001/jamanetworkopen.2023.25803 (PMC10372701; doi:10.1001/jamanetworkopen.2023.25803)
Supplement: Supplement 2. — eFigure. Forest Plot for First Ischaemic Stroke by Subgroup eTable. Baseline Characteristics of Participants Randomized to Aspirin or Placebo [file jamanetwopen-e2325803-s002.pdf]

Supplemental Online Content

Cloud GC, Williamson JD, Thao LTP, et al. Low-dose aspirin and the risk of stroke and intracerebral bleeding in healthy older people: secondary analysis of a randomized clinical trial. *JAMA Netw Open*. 2023;6(7):e2325803. doi:10.1001/jamanetworkopen.2023.25803

eFigure. Forest Plot for First Ischaemic Stroke by Subgroup
eTable. Baseline Characteristics of Participants Randomized to Aspirin or Placebo

This supplemental material has been provided by the authors to give readers additional information about their work.

© 2023 Cloud GC et al. *JAMA Network Open.*

Figure S1: Forest plot for first ischaemic stroke by subgroups

Subgroup	No. of Participants	Placebo events (rate per 1000 person-years)	Aspirin	Comparison HR(95%CI)	Test for heterogeneity p-value
**Age group**					**0.15**
– 65-74y	9569	62(2.9)	50(2.4)	0.82(0.57-1.20)	
– 75-84y	8555	80(4.1)	82(4.2)	1.03(0.76-1.40)	
– 85+y	990	24(12.2)	14(6.2)	0.51(0.26-0.99)	
**Gender**					**0.40**
– Male	8332	90(4.9)	72(3.9)	0.81(0.59-1.10)	
– Female	10782	76(3.1)	74(3.1)	0.98(0.71-1.35)	
**Smoking**					**0.97**
– Current	735	11(7.1)	9(6.2)	0.87(0.36-2.11)	
– Former/Never	18379	155(3.8)	137(3.3)	0.89(0.71-1.12)	
**Frailty**					**0.47**
– Frail	421	4(4.8)	6(6.8)	1.41(0.40-5.00)	
– Prefrail	7447	84(5.1)	65(3.9)	0.78(0.56-1.07)	
– Not frail	11246	78(3.1)	75(3.0)	0.97(0.71-1.34)	
**Diabetes mellitus**					**0.68**
– No	17069	139(3.6)	125(3.3)	0.90(0.71-1.15)	
– Yes	2045	27(6.2)	21(4.9)	0.79(0.45-1.41)	
**Hypertension**					**0.95**
– No	4919	33(3.0)	29(2.6)	0.87(0.53-1.44)	
– Yes	14195	133(4.2)	117(3.7)	0.89(0.69-1.14)	
**Dyslipidemia**					**0.37**
– No	6647	59(4.0)	57(3.9)	1.01(0.70-1.45)	
– Yes	12467	87(3.2)	109(3.9)	0.82(0.62-1.08)	
**Country**					**0.47**
– Australia	16703	144(3.9)	122(3.3)	0.86(0.67-1.09)	
– US	2411	22(4.1)	24(4.4)	1.08(0.60-1.92)	
**Overall**	**19114**	**166(3.9)**	**146(3.4)**	**0.89(0.71-1.11)**	

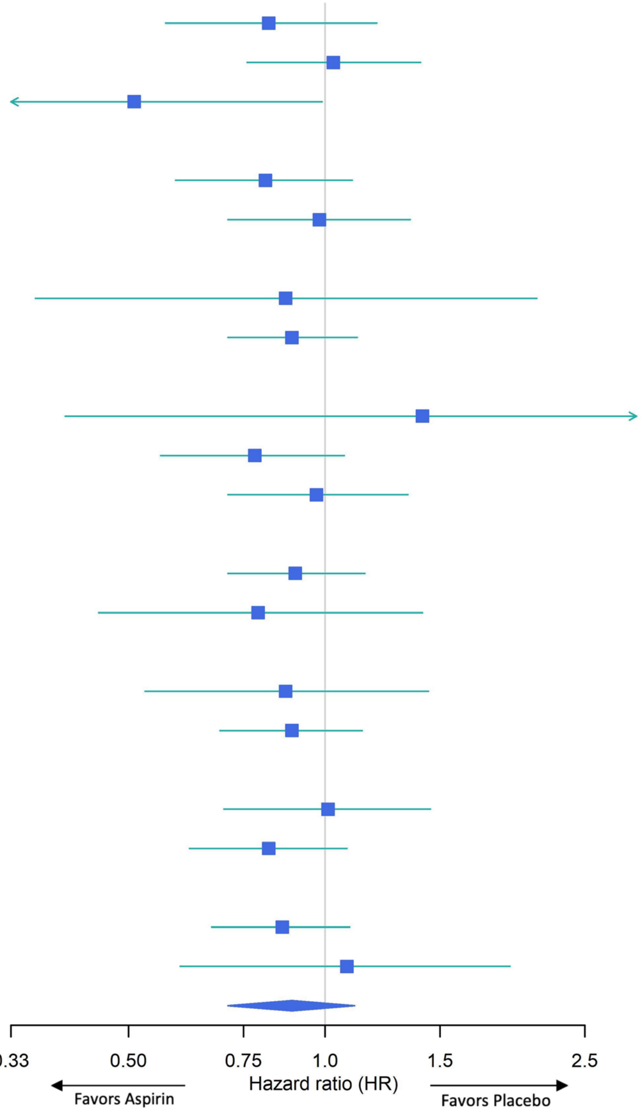

CI, confidence interval; HR, hazard ratio

© 2023 Cloud GC et al. *JAMA Network Open.*

Table S1: Baseline characteristics of ASPREE participants randomized to Aspirin or Placebo

Characteristics	Aspirin (n=9525) No. (%)	Placebo (n=9589) No. (%)
Country		
Australia	8322 (49.8)	8381 (50.2)
US	1203 (49.9)	1208 (50.1)
Age, y		
65-69	284 (3.0)	280 (2.9)
70-74	5243 (55.0)	5356 (55.9)
75-79	2533 (26.6)	2490 (26.0)
80-84	1085 (11.4)	1111 (11.6)
≥85	380 (4.0)	352 (3.7)
Male sex	4152 (43.6)	4180 (43.6)
BMI ≥25, kg/m²	6981 (73.6)	7080 (74.2)
Smoking		
Current	352 (3.7)	383 (4.0)
Former	3909 (41.0)	3890(40.6)
Never	5264 (55.3)	5316 (55.4)
Alcohol use		
Current	7309 (76.7)	7333 (76.5)
Former	566 (5.9)	570 (5.9)
Never	1650 (17.3)	1686 (17.6)
Previous regular aspirin use	1053 (11.1)	1040 (10.9)
Statin therapy	3632 (49.1)	3719 (50.1)
Hypertension	7055 (74.1)	7140 (74.5)
Dyslipidaemia	6161 (64.7)	6306 (65.8)
Diabetes	1024 (10.8)	1021 (10.7)
Frailty		
Frail	215 (2.3)	206 (2.2)
Prefrail	3707 (38.9)	3740 (39.0)
Not frail	5603 (58.8)	5643 (58.9)

© 2023 Cloud GC et al. *JAMA Network Open.*
