# Supplementary material for: Low-Dose Aspirin and the Risk of Stroke and Intracerebral Bleeding in Healthy Older People: Secondary Analysis of a Randomized Clinical Trial
Source: JAMA Netw Open. 2023 Jul 26;6(7):e2325803. doi: 10.1001/jamanetworkopen.2023.25803 (PMC10372701; doi:10.1001/jamanetworkopen.2023.25803)
Supplement: Supplement 3. — Data Sharing Statement [file jamanetwopen-e2325803-s003.pdf]

Data Sharing Statement

Cloud. Low-Dose Aspirin and the Risk of Stroke and Intracerebral Bleeding in Healthy Older People: Secondary Analysis of a Randomized Clinical Trial. *JAMA Netw Open*. Published online July 26, 2023. doi:10.1001/
jamanetworkopen.2023.25803

Data
Data available: Yes
Data types: Deidentified participant data, Data dictionary
How to access data: Requests for data access will be via the ASPREE Principal Investigators with details for applications provided through https://aspree.org/aus/for277/researchers/ or https://aspree.org/usa/for-researchers/
When available: With publication

Supporting Documents
Document types: None

Additional Information
Who can access the data: accredited researchers
Types of analyses: any purpose
Mechanisms of data availability: After approval of a proposal or with a signed data access agreement
